# Quantification Approaches in Non-Target LC/ESI/HRMS Analysis: An Interlaboratory Comparison

**DOI:** 10.1021/acs.analchem.4c02902

**Published:** 2024-10-01

**Authors:** Louise Malm, Jaanus Liigand, Reza Aalizadeh, Nikiforos Alygizakis, Kelsey Ng, Emil Egede Fro̷kjær, Mulatu Yohannes Nanusha, Martin Hansen, Merle Plassmann, Stefan Bieber, Thomas Letzel, Lydia Balest, Pier Paolo Abis, Michele Mazzetti, Barbara Kasprzyk-Hordern, Nicola Ceolotto, Sangeeta Kumari, Stephan Hann, Sven Kochmann, Teresa Steininger-Mairinger, Coralie Soulier, Giuseppe Mascolo, Sapia Murgolo, Manuel Garcia-Vara, Miren López de Alda, Juliane Hollender, Katarzyna Arturi, Gianluca Coppola, Massimo Peruzzo, Hanna Joerss, Carla van der Neut-Marchand, Eelco N. Pieke, Pablo Gago-Ferrero, Ruben Gil-Solsona, Viktória Licul-Kucera, Claudio Roscioli, Sara Valsecchi, Austeja Luckute, Jan H. Christensen, Selina Tisler, Dennis Vughs, Nienke Meekel, Begoña Talavera Andújar, Dagny Aurich, Emma L. Schymanski, Gianfranco Frigerio, André Macherius, Uwe Kunkel, Tobias Bader, Pawel Rostkowski, Hans Gundersen, Belinda Valdecanas, W. Clay Davis, Bastian Schulze, Sarit Kaserzon, Martijn Pijnappels, Mar Esperanza, Aurélie Fildier, Emmanuelle Vulliet, Laure Wiest, Adrian Covaci, Alicia Macan Schönleben, Lidia Belova, Alberto Celma, Lubertus Bijlsma, Emilie Caupos, Emmanuelle Mebold, Julien Le Roux, Eugenie Troia, Eva de Rijke, Rick Helmus, Gaëla Leroy, Niels Haelewyck, David Chrastina, Milan Verwoert, Nikolaos S. Thomaidis, Anneli Kruve

**Affiliations:** 1 Department of Materials and Environmental Chemistry, Stockholm University, Svante Arrhenius väg 16, 11418 Stockholm, Sweden; 2 Quantem Analytics, 51008 Tartu, Estonia; 3 Laboratory of Analytical Chemistry, Department of Chemistry, National and Kapodistrian University of Athens, Panepistimiopolis Zografou, 15771 Athens, Greece; 4 Department of Environmental Health Sciences, Yale School of Public Health, Yale University, New Haven, Connecticut 06510, United States; 5 Environmental Institute, Okružná 784/42, 97241 Koš, Slovak Republic; 6 RECETOX, Faculty of Science, Masaryk University, Kamenice 753/5, Building D29, 62500 Brno, Czech Republic; 7 Environmental Metabolomics Lab, Aarhus University, Frederiksborgsvej 399, 4000 Roskilde, Denmark; 8 Department of Environmental Science, Stockholm University, Svante Arrhenius väg 8, 11418 Stockholm, Sweden; 9 Analytisches Forschungsinstitut für Non-Target Screening GmbH (AFIN-TS), Am Mittleren Moos 48, 86167 Augsburg, Germany; 10 Acquedotto Pugliese SpA - Direzione Laboratori e Controllo Igienico Sanitario (DIRLC), 70123 Bari, Italy; 11 Agenzia Regionale per l’Ambiente Toscana, Via G. Marradi 114, 57126 Livorno, Italy; 12 Department of Chemistry, University of Bath, Bath BA2 7AY, U.K.; 13 Institute for Sustainability, Bath BA2 7AY, U.K.; 14 Department of Chemistry, Vienna, BOKU University, Muthgasse 18, 1190 Vienna, Austria; 15 BRGM, 3 avenue Claude Guillemin, BP36009, 45060 Orléans Cedex 2, France; 16 Water Research Institute (IRSA), National Research Council (CNR), Via F. De Blasio, 5, 70132 Bari, Italy; 17 Research Institute for Geo-Hydrological Protection (IRPI), National Research Council (CNR), Via Amendola, 122/I, 70126 Bari, Italy; 18 Water, Environmental and Food Chemistry Unit, Institute of Environmental Assessment and Water Research, C/Jordi Girona 18-26, ES 08034 Barcelona, Spain; 19 Eawag, Swiss Federal Institute of Aquatic Science and Technology, Überlandstrasse 133, 8600 Dübendorf, Switzerland; 20 White Lab Srl, Via Mons. Rodolfi 22, 36022 San Giuseppe de Cassola (VI), Italy; 21 Department for Organic Environmental Chemistry, Helmholtz Centre Hereon, Max-Planck-Str. 1, 21502 Geesthacht, Germany; 22 Het Waterlaboratorium, J.W. Lucasweg 2, 2031 BE Haarlem, The Netherlands; 23 Human Exposure to Organic Pollutants Unit, Institute of Environmental Assessment and Water Research, C/Jordi Girona 18-26, ES 08034 Barcelona, Spain; 24 Institute for Analytical Research, Hochschulen Fresenius gem. Trägergesellschaft mbH, 65510 Idstein, Germany; 25 Institute for Biodiversity and Ecosystem Dynamics, University of Amsterdam, 1012 WP Amsterdam, Netherlands; 26 Water Research Institute (IRSA), National Research Council of Italy (CNR), via del Mulino, 19, 20861 Brugherio, MB, Italy; 27 Analytical Chemistry Group, Department of Plant and Environmental Sciences, University of Copenhagen, Thorvaldsenvej 40, 1871 Frederiksberg, Denmark; 28 KWR Water Research Institute, Groningenhaven 7, 3433 PE Nieuwegein, The Netherlands; 29 Luxembourg Centre for Systems Biomedicine (LCSB), University of Luxembourg, 6, Avenue du Swing, L-4367 Belvaux, Luxembourg; 30 Center for Omics Sciences (COSR), IRCCS San Raffaele Scientific Institute, 20132 Milan, Italy; 31 Bavarian Environment Agency, Bürgermeister-Ulrich-Str. 160, 86179 Augsburg, Germany; 32 Laboratory for Operation Control and Research, Zweckverband Landeswasserversorgung, Am Spitzigen Berg 1, 89129 Langenau, Germany; 33 NILU, Instituttveien 18, 2007 Kjeller, Norway; 34 US National Institute of Standards and Technology, 331 Fort Johnson Rd, 29412 Charleston, South Carolina, United States; 35 Queensland Alliance for Environmental Health Sciences, The University of Queensland, Woolloongabba, Queensland 4102, Australia; 36 Ministry of Infrastructure and Water Management, Rijkswaterstaat Laboratory, Zuiderwagenplein 2, 8224 AD Lelystad, The Netherlands; 37 SUEZ-CIRSEE, 38 rue du president Wilson, 78230 Le Pecq, France; 38 Universite Claude Bernard Lyon 1, CNRS, ISA, UMR5280, 5 rue de la Doua, F-69100 Villeurbanne, France; 39 Toxicological Centre, University of Antwerp, Universiteitsplein 1, 2610 Antwerp, Belgium; 40 Environmental and Public Health Analytical Chemistry, Research Institute for Pesticides and Water, University Jaume I, 12006 Castelló, Spain; 41 Department of Aquatic Sciences and Assessment, Swedish University of Agricultural Sciences, 75007 Uppsala, Sweden; 42 LEESU, Univ Paris Est Creteil, Ecole des Ponts, F-94010 Creteil, France; 43 Univ Paris Est Creteil, CNRS, OSU-EFLUVE, F-94010 Creteil, France; 44 IBED Environmental Chemistry and Mass Spectrometry Laboratories, University of Amsterdam, Science Park 904, 1098 XH Amsterdam, The Netherlands; 45 VEOLIA Recherche et Innovation, Chemin de la Digue, 78600 Maisons-Laffitte, France; 46 Vlaamse Milieumaatschappij, Raymonde de Larochelaan 1, 9051 Gent, Sint-Denijs-Westerem, Belgium; 47 T. G. Masaryk Water Research Institute, p. r. i., Macharova 5, 70200 Ostrava, Czech Republic; 48 WLN, Rijksstraatweg 85, 9756 AD Glimmen, Groningen, The Netherlands

## Abstract

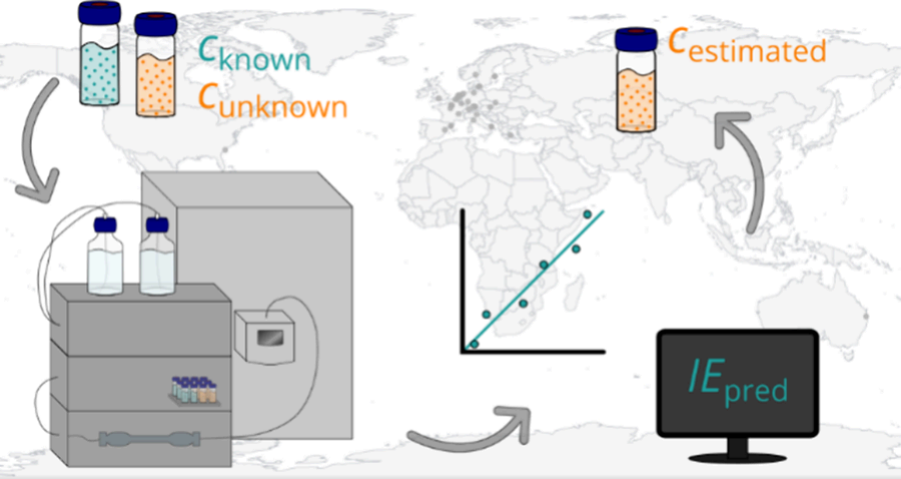

Nontargeted screening (NTS) utilizing liquid chromatography electrospray ionization high-resolution mass spectrometry (LC/ESI/HRMS) is increasingly used to identify environmental contaminants. Major differences in the ionization efficiency of compounds in ESI/HRMS result in widely varying responses and complicate quantitative analysis. Despite an increasing number of methods for quantification without authentic standards in NTS, the approaches are evaluated on limited and diverse data sets with varying chemical coverage collected on different instruments, complicating an unbiased comparison. In this interlaboratory comparison, organized by the NORMAN Network, we evaluated the accuracy and performance variability of five quantification approaches across 41 NTS methods from 37 laboratories. Three approaches are based on surrogate standard quantification (parent-transformation product, structurally similar or close eluting) and two on predicted ionization efficiencies (RandFor-*IE* and MLR-*IE*). Shortly, HPLC grade water, tap water, and surface water spiked with 45 compounds at 2 concentration levels were analyzed together with 41 calibrants at 6 known concentrations by the laboratories using in-house NTS workflows. The accuracy of the approaches was evaluated by comparing the estimated and spiked concentrations across quantification approaches, instrumentation, and laboratories. The RandFor-*IE* approach performed best with a reported mean prediction error of 15× and over 83% of compounds quantified within 10× error. Despite different instrumentation and workflows, the performance was stable across laboratories and did not depend on the complexity of water matrices.

## Introduction

Access to clean water for drinking, health, and sanitation purposes is a fundamental human right,^1,2^ and ground- and surface water play an essential role in providing safe water for such purposes.^3−5^ At the same time, thousands of compounds are used daily worldwide, e.g., in agriculture, industry, and for personal use, and many end up in the aquatic environment via e.g., insufficient wastewater treatment or stormwater runoff.^5−11^ The situation becomes more complex when considering metabolites, transformation products (TPs, which include metabolites), and disinfection byproducts. These compounds can form both naturally in the environment and during water purification processes.^12−20^ To ensure access to high quality drinking water, it is important to minimize environmental contaminants. Most countries and/or jurisdictions have some water monitoring legislation in place, which requires systematic monitoring. In the EU, this is implemented by several directives for the protection of groundwater^21^ and surface water,^22^ and to ensure the quality of water for human consumption.^23^ Currently, these regulations cover only a fraction of the compounds that may enter the aquatic environment.^9,24^ However, for regulatory decisions quantitative information on detected compounds is essential to assess their environmental and health risks.

One of the most commonly used techniques for water analysis is liquid chromatography electrospray ionization high-resolution mass spectrometry (LC/ESI/HRMS).^20,25−27^ The increased sensitivity, accuracy, and resolving power of HRMS has enabled the identification of a large number of polar and semipolar organic micropollutants.^26,28^ Due to the large number of contaminants in water samples, analysis has shifted from targeted to suspect and nontargeted approaches.^29,30^ Instead of targeting specific compounds, the sample is screened for all detected mass-to-charge ratios (*m*/*z*). Tentative identification of either suspect list matched or all detected features (unique pair of retention time (RT) and accurate *m*/*z*) without the use of analytical standards are aimed for.^27,31^ Still, the purchase and analysis of the analytical standard is ultimately required for unambiguous verification.^32^ However, since the analysis of one sample can result in tens of thousands of detected features, it is unfeasible, if not impossible, to obtain reference standards for all tentatively identified compounds.^12,25,33^

Although LC/ESI/HRMS is currently the analysis technique of choice, quantification is inherently limited. The ionization efficiency (*IE*) in ESI is highly dependent on the physicochemical properties of the compound (e.g., polarity,^34−37^ acid–base properties,^35,36,38,39^ molecular volume^36−38^), the properties of the eluent used,^36,40,41^ and the ionization source geometry.^42,43^ Therefore, the *IE* of compounds can differ by several orders of magnitude.^31^ Consequently, the signals obtained from LC/ESI/HRMS analysis do not indicate the absolute concentration of the compound in the sample. Quantitative information can be obtained by the calibration curve method, which remains inaccessible before full identification. For this reason, several approaches to quantifying compounds detected with LC/ESI/HRMS NTS without analytical standards have been developed in the past decade. Some approaches use a surrogate standard (structurally similar or with similar chromatographic behavior) for quantification,^14,44−49^ while others rely on machine learning to predict the *IE* of the detected compounds and then apply the predicted *IE* for quantification.^9,36,44,50−56^ Recently, these approaches were evaluated on pesticides, pharmaceuticals, and their TPs, finding that *IE*-based prediction models provide the most accurate results.^44^ This comparison was based on samples analyzed on one instrument in one laboratory. Therefore, it is unclear how much the accuracy of the quantification approaches relies on the instrument and/or used processing software, or the analyst’s experience.

In this NORMAN interlaboratory study of 37 laboratories in Europe, North America, and Australia, five quantification approaches without analytical standards were tested and evaluated. Specifically, three approaches with surrogate standard quantification and two approaches based on predicted ionization efficiencies were compared. An overview of the quantification approaches is given in Supporting Information SI 1. For more information including their strengths and limitations, see reviews by Malm et al.,^57^ Sepman et al.,^58^ and Hollender et al.^59^ Each participating laboratory analyzed 15 samples using their standard nontargeted LC/ESI/HRMS workflow. However, a suspect list of spiked compounds was provided. The samples consisted of three water matrices spiked with 45 compounds, including industrial, agrochemicals, food additives, drugs, personal care products, and natural products at two concentration levels. The concentrations of the spiked compounds were unknown to the participants. Furthermore, standard solutions of 41 compounds in ultrapure water at six known concentrations and three blank matrices were shipped to all participants. The study aimed to (1) compare the variability of performance and accuracy of the five quantification approaches across laboratories; and (2) evaluate the instrumental effects on their performances.

## Experimental Section

### Chemicals and Solvents

The chemicals and solvents used in this study can be found in Table S1. All chemicals were of analytical standard quality and were bought from Sigma-Aldrich, Merck, Riedel-de-Haën, Honeywell Fluka, or Dr. Ehrenstorfer GmbH. All solvents used for dissolving the chemicals were from Honeywell Riedel-de-Haën, except hydrochloric acid, formic acid, and phosphoric acid, which were from VWR chemicals.

### Samples

Stock solutions of all chemicals, as well as three mixes (calibration mix, suspect mix, and isotope-labeled internal standard (ILIS) mix), were prepared by weighing. Stock solutions were prepared from fourth of October to 14th of October 2021, and the mixes were prepared on 28th of October 2021. Surface water from Drevviken lake in Stockholm, Sweden (coordinates N59.2484796, E18.1252966), obtained on 26th of October 2021, was filtered using a Munktell Filter Paper (Ahlstrom Munksjö) and stored at +4 °C until spiking. HPLC grade water (samples s1), tap water (samples s2), and filtered surface water (samples s3) were spiked with the suspect mix to a concentration range from 6.70 × 10^–8^ to 5.89 × 10^–6^ M (14–780 μg/L, samples a). Additionally, each sample was diluted 10× (samples b). The calibration mix was prepared in HPLC grade water at six concentration levels ranging from 8.49 × 10^–10^ to 8.90 × 10^–6^ M (0.6–1000 μg/L). Both suspect samples and calibration mixes were spiked with ILIS mix at a constant concentration of 1.30 × 10^–7^ to 2.12 × 10^–7^ M (40–50 μg/L). All samples were prepared on the 28th of October 2021. The final concentration of the chemicals in the samples was determined via a calibration curve and can be found in Tables S2 and S3.

1 mL aliquots of samples and calibration mixes, as well as blanks of each water matrix, were transferred to transparent HPLC vials directly after preparation and were stored at −20 °C before shipping to participating laboratories (see Table S4 and Figure S1 for laboratories and geographical position). The frozen samples, totaling 15 per laboratory, were sent to participants within Europe on November first, 2021, and to participants outside Europe on November fifth, 2021. Within Europe, samples arrived within 3 days, while for participants outside Europe ranged from 4 to 7 days. All samples were analyzed within 3 months from shipping, and all participants but one stored the samples at −20 °C until analysis.

### Instrumental

For instrumental details, see SI 2 and Table S5 for an easier overview.

All samples were analyzed with LC/HRMS from various vendors in positive ESI mode, following the NTS workflow of individual laboratories/institutes. The samples were analyzed either as single measurements (*n* = 2), duplicates (*n* = 9) or triplicates (*n* = 30).

### Stability Tests

Suspect samples (undiluted and 10× diluted) and two calibration mixes (high and one concentration), were stored under different conditions: (1) freeze–thaw cycles (stored in the freezer and thawed for each analysis), (2) in the fridge (4 °C), (3) at room temperature (20–25 °C), and (4) in the freezer (−20 °C). These, alongside freshly made calibration solutions (six concentrations), were analyzed once a week for 8 weeks, then once every other week for an additional 6 weeks. Concentrations were calculated from the calibration curve made each week. Analysis was performed using a Dionex UltiMate 3000 UHPLC - Q Exactive Orbitrap HRMS (Thermo Fisher Scientific). See SI 2 - DS_QDF for further analysis details and SI 4, Figures S2–S5 and Table S6 for the results.

### Data Treatment

Approximately 50% of the participants received the samples as frozen or below room temperature, and all except one reported that samples were stored in a freezer until analysis. Compounds showing signs of degradation when stored in the freezer (ampicillin, dazomet, and simvastatin) were removed from all data sets for all samples and were not considered in the following statistics. In addition, one laboratory indicated that the samples were stored in a fridge before analysis. Therefore, compounds showing significant degradation in the stability experiments when stored in the fridge were removed from this data set (SI 4, Figures S2–S5 and Table S6b).

#### Reported Data from Participants

All detected compounds were quantified using the five approaches described in SI 1 (parent–TP approach (see Table S7a for parent-TP pairs), structurally similar approach (see Table S7b for most similar assignment, note that for reported results, only top 1 most similar compound was used), close eluting approach, RandFor-*IE* approach, and MLR-*IE* approach). The first three approaches were calculated automatically in Excel workbooks provided to all participants together with the samples (see Supporting Workbook File SWF1) while the two latter approaches were calculated using online platforms: Quantem software version 0.3^60^ for RandFor-*IE* and Semi-Quantification of Emerging Pollutants application version 1.0.0^61^ for MLR-*IE* approaches. Results from participating laboratories were received as Excel workbooks with calculated concentrations for all approaches and their raw data. All automatic calculations were based only on the peak area of the detected peak, without accounting for ILIS signal variation, unless implemented by individual participants. These results are reported as received from the participants. The data was evaluated using R v. 4.2.1.^62^ In total, 41 data sets with corresponding raw data were received from 37 laboratories. For three laboratories, the raw data was either inaccessible or missing, and for one laboratory, only raw LC/HRMS data files were submitted. All raw files are available in the NORMAN Digital Sample Freezing Platform.^63,64^

#### Reprocessed Data

All raw data were reprocessed using *patRoon* package v. 2.2.0 in R.^65^ Due to the variation in instrumentation used, the parameters for peak picking and filtering were optimized individually for all data sets using in-house scripts. Mainly, parameters regarding signal intensity were varied, and for a few laboratories, a wider *m*/*z* window was used, see SI 3 for further details and settings used for each data set. Obtained peak areas were normalized to atrazine-d_5_ (eq 1).
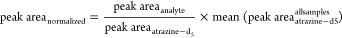
1

For quality assessment of the peaks, the normalized peak areas of calibration compounds were plotted against concentrations. The graphs were then manually inspected for linearity based on residual analyses. All nonlinear data points and graphs with fewer than three data points in the linear range were removed from further analysis. The expected dead time was estimated for each laboratory based on column dimensions and flow rate. Compounds (both calibrants and suspects) with RT shorter than the estimated dead time were removed. For suspect compounds, the peak area ratio of high to low concentration samples for each matrix were computed. Ratios below 5 or above 20 (the theoretical ratio being 10 for measurements in the linear range) were removed. These points are denoted as out-of-range ratios throughout the paper. The remaining normalized peak areas were used to estimate the concentrations according to the five quantification methods.

### Evaluation of Quantification Accuracy

The accuracy of the quantification approaches was evaluated mainly based on fold error (eq 2), while log error (eq 3) was used to detect trends of over- or underpredictions. Throughout this study, fold error up to 10× (corresponding to log error between −1 and 1) was considered sufficiently accurate for risk assessment in NTS.^66^
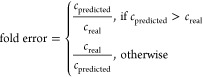
2
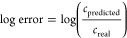
3

Comparison of prediction errors between data sets and quantification approaches were guided by visual inspection, and findings were supported with statistical tests. Due to the non-normally distributed errors and a varying number of replicates analyzed by participants, the Friedman test was used to evaluate the statistical significance of mean fold errors across data sets and quantification approaches. Since the Friedman test requires that the groups compared have the same size, incomplete data sets (i.e., where results from one or more quantification approaches were missing) were omitted from the test. For quantification approaches, significant Friedman tests were followed with Nemenyi’s all-pairs comparisons test. The Wilcoxon signed-rank test with Bonferroni adjusted *p*-values was used to evaluate the effects of the matrix. For this, the peak areas obtained in tap- or surface water were pairwise compared to peak areas obtained in HPLC-grade water at both concentration levels separately. HPLC water was considered a matrix-free medium. Peak areas were used instead of prediction error to exclude the influence of the quantification approach. The statistical significance was determined at the 95% confidence level.

Outliers were investigated by visual inspection of box-and-whisker plots of fold errors for each approach and data set. Outliers were compared across data sets to identify trends of compounds yielding poor estimates for certain approaches.

## Results and Discussion

### Compound Selection and Stability Evaluation

**Figure 1:**
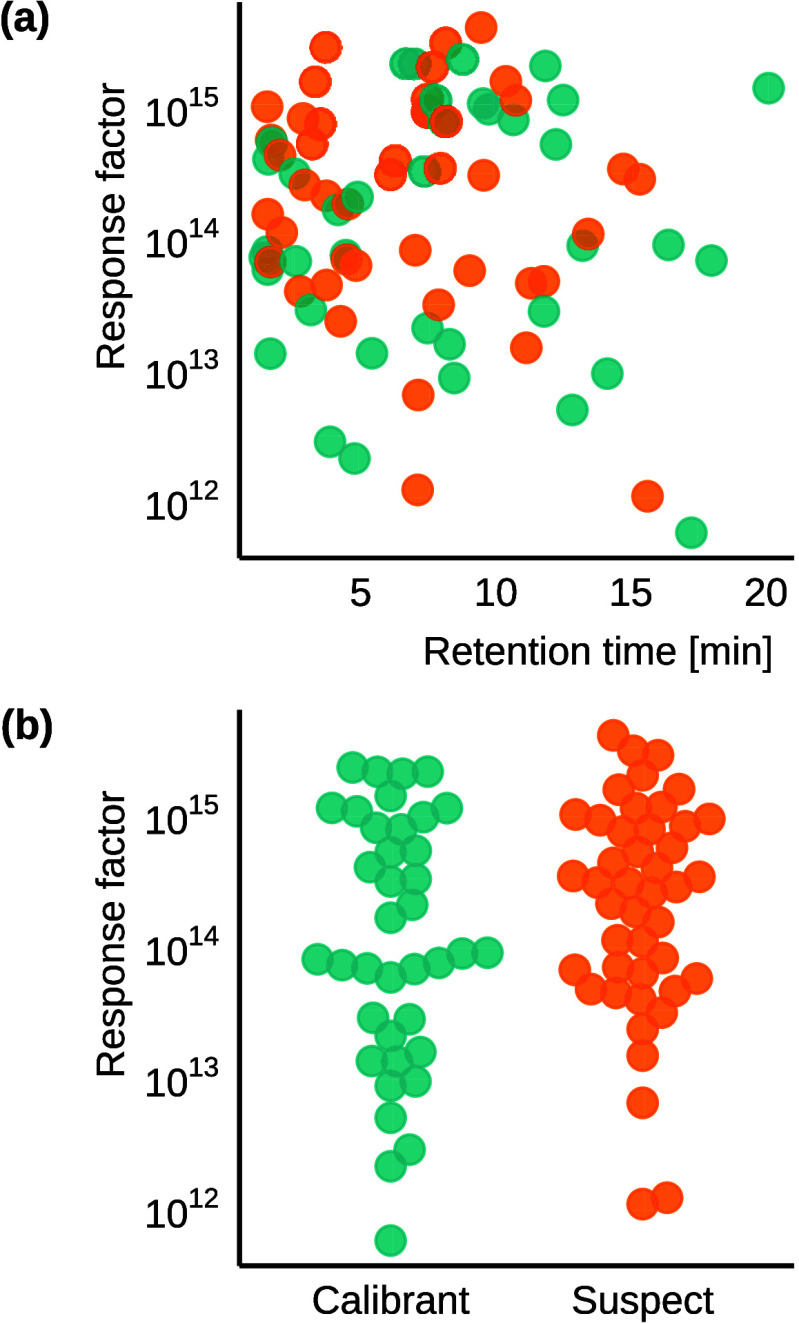
Distribution of the selected compounds spiked to the water samples: (a) range of retention times and the response factors of the compounds in a 25 min gradient (see SI 2 - DS_QDF for analysis details), and (b) distribution of the response factors of calibration and suspect compounds.

Compounds were selected based on environmental relevance, with a focus on water, aiming to cover a wide chemical space. This included compounds with varying polarity and ionization potential, as well as those with known TPs. Compounds were selected from NORMAN SusDat^67^ and based on information from the literature.^68^Figure 1a shows the distribution of the compounds based on response factor (RF) and retention time, while Figure 1b visualizes the RF distribution of calibration and suspect compounds for one lab. The RF for each compound was calculated as the ratio of peak area and concentration, see SI 2 - DS_QDF for analysis details.

### Concentration Estimates Reported by Participants

**Figure 2:**
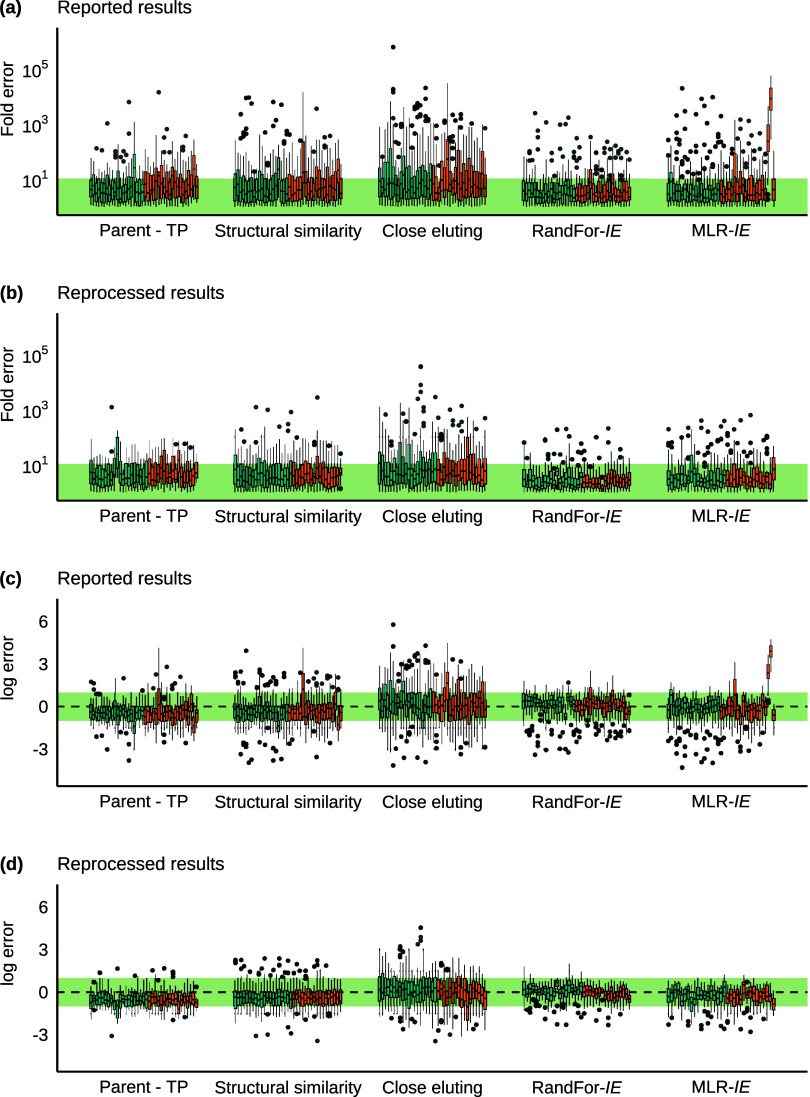
Prediction errors of each quantification approach across the data sets for sample s1a (high concentrated spike in HPLC water). The green area shows the 10× error and the equivalent log error. Blue boxes are from analysis on orbitrap HRMS, while orange boxes are from analysis on ToF HRMS. The fold errors, calculated according to eq 2, for reported data are displayed in (a), and corresponding log errors, calculated according to eq 3, are shown in (c). (b) and (d) show the corresponding graphs for the reprocessed data.

Analysis of the reported concentrations from all participants revealed some trends. The ionization efficiency-based approaches generally yielded better quantification accuracy than the surrogate standard-based approaches. For these two approaches, the majority of compounds across samples and data sets had errors within a factor of 10, with some exceptions. The results for the MLR-*IE* approach from two data sets deviated more than the other data sets (Figure 2a). However, the close eluting approach yielded the overall highest fold errors, with up to approximately 1,000,000×. Similar general trends could be seen across data sets despite the use of different instruments. In Table 1, the mean, median, and 95% quantile fold error over all data sets and samples, along with the percentage of estimations within 10× error for the quantification approaches, is shown (for each data set and sample, please see Table S8). Analysis of the log error (Figure 2c) revealed that most approaches were more prone to underprediction, especially parent-TP and structural similarity approaches. The ionization efficiency-based approaches and the close eluting approach were less prone to underpredictions. However, for the former approaches, almost all outliers were underpredicted. Underprediction is undesirable, as consistent underpredictions may result in overlooking compounds present at environmental/ecotoxicological relevant concentrations. Instead, slight overprediction is preferable for quantitative estimates to ensure that all possible hazardous compounds are included for further investigations, especially for environmental analysis and risk assessments.^69^

**Table 1:** Mean, Median, and 95% Quantile Fold Errors, along with the Percentage of Estimation within 10× Error for the Quantification Approaches over All Datasets and Samples

approach	mean fold error		median fold error		95% quantile fold error		% less than 10× error	
	reported	reprocessed	reported	reprocessed	reported	reprocessed	reported (%)	reprocessed (%)
parent-TP	140×	13×	3.9×	3.4×	79×	44×	71.8	75.5
structural similarity	100×	17×	4.0×	3.4×	110×	53×	70.4	75.5
close eluting	1 200×	150×	6.0×	5.0×	570×	180×	60.3	65.1
randFor-*IE*	15×	5.5×	3.0×	2.4×	28×	17×	83.9	91.2
MLR-*IE*	3 000×	11×	3.6×	2.8×	500×	32×	75.5	83.4

Statistically significant differences between the peak areas obtained in different matrices and concentration levels (adjusted *p*-values ≪0.05, see Table S9) were observed. However, only a minor effect on the overall results was observed, as the prediction errors were mostly coherent across the samples (Figures 2 and S6–S15). The significant but nonsystematic differences indicate that matrix effects largely depend on the specific combination of sample and chromatography. Moreover, for the water samples in this study, the impact of the matrix effect appears to be smaller than the inaccuracies of the quantification approaches. The average fold errors were compared across the data sets using the Friedman test, and statistically significant differences were observed for all samples (*p*-value ≪0.05, see Table S9a). Similarly, the average fold errors across the quantification approaches were also statistically different in all samples (Friedman test followed by Nemenyi’s all-pairs comparisons test, *p*-values ≪0.05, see Table S9b–h).

#### Outlier Analysis

To evaluate trends of compounds frequently associated with high errors, the outliers (points outside the whiskers, i.e., more than 1.5 times the length of the box (50th percentile)) from the box and whisker plot (Figure 2) were investigated. However, due to the variations between data sets, compounds that were outliers in one data set might yield high but nonoutlying errors in another data set. On the other hand, only a few outliers were within 10× error (corresponding −1 to 1 log error), and these were not considered for the outlier analysis. Since the analysis involved two error metrics (fold error and log error), five quantification approaches, six samples, and 40 data sets, each compound could be determined as an outlier with a maximum of 2 400 occurrences, depending on the detection frequency in each data set. Although no clear trends were seen, some compounds appeared as outliers across multiple data sets or occurred with higher frequency but across fewer data sets. For example, methomyl, atrazine-desethyl-desisopropyl, sudan I, chlorpyrifos, and benzothiazole occurred as outliers in the largest number of data sets; 22 (55%), 22 (55%), 20 (50%), 19 (47.5%), and 18 (45%) data sets, respectively. Similarly, methomyl, butylamine, clotrimazole, naproxen, and chlorpyrifos were the most frequent outliers, with 98, 41, 57, 49, and 58 occurrences, respectively, corresponding to outlier rates (eq 4) of 7.8, 5.9, 5.4, 5.3, and 4.2% when considering the detection frequency.
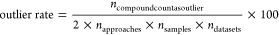
4

These outliers all belong to different compound classes, with ion masses ranging from 74 to 350 Da and RTs spanning from very early to late eluting (Table S12). Therefore, no general conclusions could be drawn for why these specific compounds were frequently occurring outliers. Instead, the peak area ratios between high and low spikes in each sample were inspected to see if compounds with deviating ratios also yielded the highest errors and thus would be considered outliers. The compounds with out-of-range ratios were generally not the most frequent outliers, and vice versa (see Figure S16). Sometimes compounds with out-of-range ratios were not even considered outliers nor had particularly high prediction errors. For example, reserpine was found with a ratio above 20 in the majority of data sets independent of the matrix but was only found as an outlier in five data sets. The highest ratio was found in HPLC water, and the corresponding prediction errors for this data set ranged from 1.2× to 33× depending on the quantification approach, which is relatively narrow compared to e.g., simazine-2-hydroxy where the error in one data set and matrix ranged over 6 orders of magnitude.

#### The Impact of Quantification Approaches on Prediction Error

There can be several reasons for the high prediction errors observed for the quantification approaches, and they might differ depending on the approach. For all approaches except the close eluting approach, a tentative structure is required for the quantification. Therefore, the errors may partly be attributed to the uncertainty of compound annotation, which should be clearly communicated with the quantitative estimates.^32,70^ Here, this error source was limited due to the provided suspect list, aiming for a fairer evaluation of the approaches themselves. However, in reality this is a real issue, likely to increase the errors, especially for incorrect or uncertain annotation.

Since the RF of a surrogate standard is used to quantify the suspect/unknown in surrogate standard quantification, wide differences in the responses between surrogate and suspect/unknown are likely to cause higher errors. For example, TPs are generally less hydrophobic than their parent compounds resulting in reduced ionization efficiency^44,57^ and consequently lower response. This can explain the general underprediction seen in the parent–TP approach. This also affects the prediction error for the structural similarity approach in this study, as a large portion of the spiked compounds were TPs and their most similar surrogate standards were predominantly the respective parent chemicals. However, sometimes another calibration compound was more structurally similar than the parent compound, see Table 2. For the two benzothiazoles, the most similar compound was benzotriazole, while for the atrazine TPs, simazine was more similar than their parent and for metformin, caffeine was more structurally similar than guanylurea. The RFs (from analysis according to SI 2 - DS_QDF) for these TPs were generally more similar to those of the structurally most similar compound than to their respective parent compounds’ response factors. Still, differences up to nearly 2 orders of magnitude were observed (Table 2). For these TPs, the average prediction error was improved in the structural similarity approach compared to the parent-TP approach, indicating that structurally similar chemicals might be better suited for quantification if TPs have very different structures than their parent compounds.^44^

**Table 2:** TPs Which Had Another Structurally More Similar Calibration Compound than Their Parent with the RFs of TP, Parent and Most Similar Compound^a^

		parent–TP approach			structural similarity approach		
suspect	RF^b^	parent	RF^b^	mean fold error	most similar	RF^b^	mean fold error
2-aminobenzothiazole	5.2 × 10^14^	TCMTB	2.7 × 10^13^	29×	benzotriazole	2.1 × 10^14^	9.1×
benzothiazole	3.1 × 10^13^	TCMTB	2.7 × 10^13^	44×	benzotriazole	2.1 × 10^14^	30×
atrazine-desethyl-desisopropyl	4.6 × 10^13^	atrazine	1.1 × 10^15^	1 800×	simazine	7.7 × 10^14^	1 500×
atrazine-desethyl-desisopropyl-2-hydroxy	3.9 × 10^13^	atrazine	1.1 × 10^15^	110×	simazine	7.7 × 10^14^	70×
atrazine-desisopropyl	6.2 × 10^13^	atrazine	1.1 × 10^15^	160×	simazine	7.7 × 10^14^	25×
atrazine-desisopropyl-2-hydroxy	5.6 × 10^14^	atrazine	1.1 × 10^15^	7.3×	simazine	7.7 × 10^14^	6.0×
metformin^c^	1.0 × 10^15^	guanylurea	7.9 × 10^13^	18×	caffeine	7.4 × 10^13^	15×

a The mean fold error, averaged over all datasets and samples for these two approaches are also shown.

b RFs calculated from analysis described in SI 2 - DS_QDF, and might differ between data sets.

c Metformin is actually the parent compound to guanylurea, but here they were switched due to initial model development requirements.

For the suspect compounds included in this study, the majority of them had lower response factors than their structurally most similar or parent compounds, with the exceptions of 2-aminobenzothiazole, metformin, methomyl, Monuron, naproxen, and thiabendazole. The largest difference in RF of 3 orders of magnitude was seen for chlorpyrifos and its most similar calibrant metolachlor. However, large differences in RF between suspect and calibration compounds alone could not explain the highest absolute errors. For example, as seen in Table 2, the RF difference between atrazine-desethyl-desisopropyl and atrazine is approximately the same as the difference between atrazine-desethyl-desisopropyl-2-hydroxy and atrazine, yet the average fold error is substantially different. Regarding the close eluting approach, the RTs can shift considerably depending on the chromatographic conditions used in the analysis.^71^ As a result, the assignment of the closest eluting compound varies across data sets with different LC conditions. In this study, although reversed-phase chromatography was mainly used, the column dimensions, particle sizes, and stationary phases, as well as the mobile phase compositions, additives, and pH varied across analyses. Consequently, different calibration compounds were assigned as the closest eluting to the same suspect compound across the data sets, demonstrating the instability of this approach. The close eluting assignments fluctuated from four calibration compounds for metformin to 16 different calibrants for metolachlor-ESA. The same chemical (benzotriazole) occurred as the close eluting compound most often (24 data sets) for atrazine-desisopropyl. For clotrimazole, the largest proportion of the same close-eluting compound across data sets was much smaller, with clarithromycin occurring as close-eluting in five data sets. However, the fluctuations in close eluting assignments across laboratories did not seem to have much influence on the prediction errors. For example, metolachlor-ESA, with its 16 different close eluting assignments, had lower average fold error and standard deviation than both metformin and atrazine-desisopropyl, as seen in Figure S17. Simazine-2-hydroxy, which had the highest average fold error, had in total 13 different closest eluting compounds across the data sets, compared to nine assignments for benzotriazole-5-carboxylic acid with the lowest average error.

**Figure 3:**
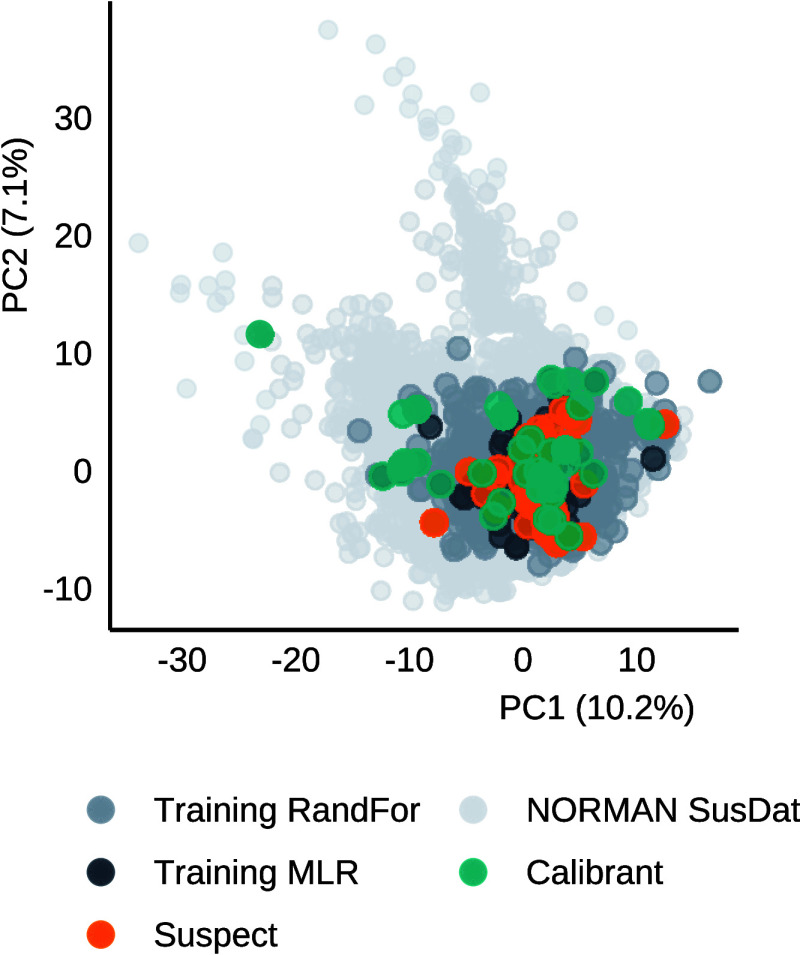
PCA (first two components) for calibrants and suspects in this study, along with the training compounds for *IE*-based models and the LC/ESI(+) amenable compounds from NORMAN SusDat (Prob. RPLC ≥ 0.5, Prob. + ESI ≥ 0.5. PCA based on Mordred descriptors.

For the ionization efficiency-based approaches higher errors are expected for compounds outside the chemical space for which the models were trained. While the online application used for the MLR-*IE* approach provides information about whether the suspect compound is covered by the model or not, the RandFor-*IE* approach does not, and neither gives this information about the calibration compounds used. Therefore, a principal component analysis (PCA) was performed on the suspect and calibration compounds used in this study, together with the training compounds used in the ionization efficiency models and the LC/ESI(+) amenable compounds in NORMAN SusDat (Prob. RPLC ≥ 0.5, Prob. + ESI ≥ 0.5, information available in the database). For this, Mordred descriptors^72^ were calculated for all compounds, and the four first PCs were used to assess how well the models’ training compounds cover the compounds used here, and how representative they are over the whole chemical space. However, the Mordred descriptors used in the PCA also included descriptors not used by the models for predicting the *IE* values. As seen by the first two components in Figure 3, most compounds used in this study seem to be covered by the chemical space of both models, except for a few calibration compounds. See also Figures S18 and S19 for the third and fourth component. The suspect compounds reserpine and butylamine appear to be poorly covered by the models, as they lie on the edge of the covered chemical space. Reserpine had neither the highest prediction error nor occurred as an outlier the most times for the *IE*-based approaches, while butylamine was one of the five compounds giving the highest error across several data sets for both approaches. In addition, imperfect transfer of log *IE* to log RF values may also cause higher errors, however, this was not investigated here.

The results from modeling approaches may be difficult to interpret, especially in terms of confidence, reliability and uncertainty. The NTS community is working toward including uncertainty estimates in quantitative analysis,^69^ and addition of confidence information to the estimations for the *IE* based models are under development by the research groups. For example, MLR-*IE* provides the predicted concentrations with a range,^61^ and RandFor-*IE* is now giving the confidence level of estimates in positive mode;^60^ however, this was not yet available at the time of the trial. These efforts allow for more confident interpretation of the results.

#### The Impact of Data Quality on Prediction Error

In addition to how the quantification approach influences the prediction error, the quality of the data may affect the magnitude of the errors. For example, incorrect integration of compounds, caused by e.g., misidentified compounds, noisy mass spectra, or other issues with the processing, can also lead to higher prediction errors, regardless of the quantification approach. Moreover, in-source fragmentation and adduct formation can significantly affect a compound’s RF, depending on its properties. Therefore, it has been suggested to use the sum of peaks from adducts and in-source fragments for quantification.^73^ In this study, some known adducts and in-source fragments were included in the suspect list provided to the participants; however, this was not extensively investigated before the study. Furthermore, the *IE*-based approaches included here can only be applied to protonated species, and thus, the inclusion of other adducts would not have affected these two approaches.

The two data sets with high errors for the MLR-*IE* approach were investigated to understand the source of deviating results. Originally, one of the data sets did not include reported results for the MLR-*IE* approach, but it was calculated at a later stage using reported peak areas without the quality assessment of calibration curves. This resulted in multiple negative slopes used in the harmonization step (transfer of log *IE* to log RF) of the MLR-*IE* approach, which may have had a negative influence on the results. For the second data set, no negative slopes were included in the harmonization step. However, it was found that for both data sets, over 60% of the normalized peak areas used in the final step of the MLR-*IE* approach were either larger or similar in the lower concentrated samples compared to the higher concentrated sample. Yet, the non-normalized peak areas for the two data sets showed the expected pattern, with higher peak areas for the more concentrated samples. This observation might suggest incorrect integration of atrazine-d_5_ peaks. In fact, in one of the data sets, the atrazine-d_5_ peak areas in the more concentrated samples were roughly a factor of 10 higher than in the low-concentrated samples. This would be the expected pattern for suspect compounds but not for the isotope-labeled standards, as they were spiked at the same concentration level in all samples. This discrepancy could be the cause of the outlier errors observed for the MLR-*IE* approach.

In addition, some compounds had very high errors in certain data sets and approaches; simazine-2-hydroxy and 2-aminobenzothiazole from one data set in the close eluting approach, and atrazine-desethyl-desisopropyl from another data set in the parent-TP and structural similarity approaches. Regarding simazine-2-hydroxy and 2-aminobenzothiazole, both were quantified using the same calibrant in the data set in question, namely butocarboxim (protonated species). In this data set, the ammonium and sodium adducts of butocarboxim were also reported. However, the reported RTs of the [M + H]^+^ ion and the [M + NH_4_]^+^ and [M + Na]^+^ adducts differ dramatically (4 min), with the adducts having the same retention time. Thus, it is reasonable to believe that the high errors for simazine-2-hydroxy and 2-aminobenzothiazole were due to false assignment issues in this specific data set. Regarding atrazine-desethyl-desisopropyl, for the concerned data set, it was only detected in one of the samples and only in one replicate. Therefore, the signal from atrazine-desethyl-desisopropyl from this data set is likely an artifact rather than the real signal, which could explain the high error. The examples from these data sets show the importance of data quality–including structure assignment–for accurate quantification.

### Concentration Estimates Based on Reprocessed Raw Data

The raw data from the participants were reprocessed and concentrations were recalculated to assess how well the trends observed in the reported results correlated with the reprocessed results using one consistent approach and operator. Since the reprocessing and final evaluation of peaks were done by one expert on the specific data, the workflow was more targeted than suspect or nontargeted screening. Still, the resulting prediction errors revealed similar trends as the reported results, with more accurate results for the ionization efficiency-based approaches, and the underprediction issue for most of the approaches present, as seen in Figure 2b,d. The mean fold errors were reduced the most from 3000× to 11× for the MLR-*IE* approach and the least from 15× to 5.4× for the RandFor-*IE* approach (Table 1). Although the improvements of median fold errors were less pronounced compared to the other metrics, the differences were still significant for the close eluting, RandFor-*IE*, and MLR-*IE* approaches, according to Wilcoxon signed rank test (Bonferroni adjusted *p*-values <0.05, Table S9c). For the parent-TP and structural similarity approaches, the results were not significantly improved. The dramatic changes in mean and 95% quantile error were likely due to the removal of the absolute largest errors, which have a lower impact on the median error. As seen in Figure 2b,d, the errors for the two data sets with the highest deviating results in the MLR-*IE* approach in the reported results were in the same range as other data sets in the reprocessed data, which contributed to the tremendous improvement of this approach.

The improved accuracy suggests that data processing and its quality control needs unification in suspect screening and NTS. It has been proposed to run the samples at different dilutions in NTS, for several reasons: to ensure that analysis is performed in the linear range as well as to evaluate matrix effects.^71^ Moreover, analysis of multiple dilutions may reduce the occurrences of instrumental artifacts, thanks to RT alignment across the dilutions. Similarly, the inclusion of more adducts could help further improve the identification confidence since all adducts in one analysis should have the same RT. For further recommendations regarding data processing and quality control in NTS, see guidelines by BP4NTA^74^ and reviews by Renner and Reuschenbach,^75^ and Hollender et al.^59^

Similar to the reported results, outliers were investigated in the reprocessed data. Interestingly, a few compounds were frequently found as outliers in both sets of results, namely atrazine-desethyl-desisopropyl, benzothiazole, clotrimazole, and methomyl. Atrazine-desethyl-desisopropyl was mostly found as an outlier in structural similarity- and parent-TP approaches and was quantified using the calibration curves of either atrazine or simazine. As seen in Table 2, differences in RF between the suspect and the calibrants were initially observed, which might partly explain the outlier frequency for this compound, even though the RFs will change depending on the instrument and settings used. Still, as discussed earlier, this cannot fully explain the large errors. Methomyl was predominantly found as an outlier in the structural similarity approach and had butocarboxim as the most similar compound. Both the [M + H]^+^ and [M + NH_4_]^+^ species were included for butocarboxim. However, only one of these peaks was used for quantification. In this case, it might have been advantageous to combine the peak areas of both species for RF calculation and quantification. Benzothiazole and clotrimazole were predominantly found as outliers in one or both of the two ionization efficiency-based approaches. Based on the PCA, the compounds in this study seemed to be included in the chemical space covered by the models. However, to fully assess the applicability domain of the models, and maybe reveal a reason for benzothiazole and clotrimazole being outliers in the *IE*-based approaches, further investigations outside the scope of this paper is warranted.

## Conclusions

The comparison of five commonly used quantification approaches revealed that approaches based on ionization efficiency modeling outperformed surrogate standard approaches in general, especially when considering the percentage of compounds quantified within 10× error. The RandFor-*IE* approach yielded the most accurate concentration estimates in both reported and reprocessed results on all evaluation points, while the close eluting approach yielded the highest prediction errors in both sets of results. The MLR-*IE* approach yielded the second most accurate concentration estimates in the reprocessed results after the RandFor-*IE* approach. For more than 83% of the compounds, both *IE*-based approaches provide estimated concentrations within 10 × , which is considered acceptable accuracy for quantitative nontarget screening here. The quantification approaches are, however, confined to the chemical space of calibration compounds or the compounds used when training the models used for *IE* predictions. While not a problem for the compounds used in this study, the results for compounds outside the applicability domain should be considered with caution regarding their accuracy. Moreover, out of the tested methods, only the close eluting approach can be used to quantify compounds without a tentative structure, which is an advantage compared to the other approaches here.

Similar trends were observed across the different data sets, even though different instruments and workflows were used. This suggests that while instrumental effects can affect the overall magnitude of the prediction error, the relative errors between quantification approaches remain consistent. In the reprocessed results, the prediction errors were smaller compared to the reported results; however, the same general trends were observed. This highlights the need for unified data processing and quality control across different workflows, but also the inherent inaccuracies of the quantification approaches, which will not be influenced by the quality of the data. Importantly, the errors presuppose that the correct structure is present in the sample. In reality, uncertainty related to the identification is likely to propagate to the quantification, increasing the prediction errors.

Based on the results presented in this study, the use of ionization efficiency-based quantification is recommended where possible, after first carefully assessing the quality of the obtained data. To minimize the errors related to quantification, we strongly encourage to follow recommended guidelines to NTS in general^59^ and data processing in particular.^74,75^

## Supporting Information Available

Chemicals, solvents, concentrations and participants information, results from statistical tests, error statistics, and additional outlier information (XLSX)
Example workbook used by participants for reporting the results (XLSX)
Supporting figures, detailed instrumental information for all participants, and reprocessing details (PDF)
