# Supplementary material for: Quantification Approaches in Non-Target LC/ESI/HRMS Analysis: An Interlaboratory Comparison
Source: Anal Chem. 2024 Oct 1;96(41):16215–26. doi: 10.1021/acs.analchem.4c02902 (PMC11483430; doi:10.1021/acs.analchem.4c02902)
Supplement: Supplementary file 3 — ac4c02902_si_003.pdf [file ac4c02902_si_003.pdf]

# Supporting Information

# Evaluation of Quantitative Approaches in Non-Target LC/ESI/HRMS Analysis

Louise Malm[1], Jaanus Liigand[2], Reza Aalizadeh[3,4], Nikiforos Alygizakis[3,5], Kelsey Ng[5,6], Emil Egede Frøkjær[7], Mulatu Yohannes Nanusha[7], Martin Hansen[7], Merle Plassmann[8], Stefan Bieber[9], Thomas Letzel[9], Lydia Balest[10], Pier Paolo Abis[10], Michele Mazzetti[11], Barbara Kasprzyk-Hordern[12,13], Nicola Ceolotto[12,13], Sangeeta Kumari[14], Stephan Hann[14], Sven Kochmann[14], Teresa Steininger-Mairinger[14], Coralie Soulier[15], Giuseppe Mascolo[16,17], Sapia Murgolo[16], Manuel Garcia-Vara[18], Miren López de Alda[18], Juliane Hollender[19], Katarzyna Arturi[19], Gianluca Coppola[20], Massimo Peruzzo[20], Hanna Joerss[21], Carla van der Neut-Marchand[22], Eelco N. Pieke[22], Pablo Gago-Ferrero[23], Ruben Gil-Solsona[23], Viktória Licul-Kucera[24,25], Claudio Roscioli[26], Sara Valsecchi[26], Austeja Luckute[27], Jan H. Christensen[27], Selina Tisler[27], Dennis Vughs[28], Nienke Meekel[28], Begoña Talavera Andújar[29], Dagny Aurich[29], Emma L. Schymanski[29], Gianfranco Frigerio[29,30], André Macherius[31], Uwe Kunkel[31], Tobias Bader[32], Pawel Rostkowski[33], Hans Gundersen[33], Belinda Valdecanas[33], W. Clay Davis[34], Bastian Schulze[35], Sarit Kaserzon[35], Martijn Pijnappels[36], Mar Esperanza[37], Aurélie Fildier[38], Emmanuelle Vulliet[38], Laure Wiest[38], Adrian Covaci[39], Alicia Macan Schönleben[39], Lidia Belova[39], Alberto Celma[40,41], Lubertus Bijlsma[40], Emilie Caupos[42,43], Emmanuelle Mebold[43], Julien Le Roux[42], Eugenie Troia[44], Eva de Rijke[44], Rick Helmus[44], Gaëla Leroy[45], Niels Haelewyck[46], David Chrastina[47], Milan Verwoert[48], Nikolaos S. Thomaidis[3], Anneli Kruve[1,8]*

[1] Department of Materials and Environmental Chemistry, Stockholm University, Svante Arrhenius väg 16, 11418 Stockholm, Sweden

[2] Quantem Analytics, 51008 Tartu, Estonia

[3] Laboratory of Analytical Chemistry, Department of Chemistry, National and Kapodistrian University of Athens, Panepistimiopolis Zografou, 15771, Athens, Greece

[4] Department of Environmental Health Sciences, Yale School of Public Health, Yale University, New Haven, CT, 06510 USA

[5] Environmental Institute, Okružná 784/42, 97241 Koš, Slovak Republic

[6] RECETOX, Faculty of Science, Masaryk University, Kamenice 753/5, Building D29, 62500 Brno, Czech Republic

[7] Environmental Metabolomics Lab, Aarhus University, Frederiksborgsvej 399, 4000 Roskilde, Denmark

[8] Department of Environmental Science, Stockholm University, Svante Arrhenius väg 8, 11418 Stockholm, Sweden

[9] Analytisches Forschungsinstitut für Non-Target Screening GmbH (AFIN-TS), Am Mittleren Moos 48, 86167 Augsburg, Germany

[10] Acquedotto Pugliese SpA - Area Controllo Qualitá Reti e Impianti, Viale Vittorio Emanuele Orlando, 70123 Bari, Italy

[11] Agenzia Regionale per l'Ambiente Toscana, Via G. Marradi 114, 57126 Livorno, Italy

[12] University of Bath, Department of Chemistry, Bath, BA2 7AY, UK

[13] Institute for Sustainability, Bath, BA2 7AY, UK

[14] BOKU University, Department of Chemistry, Vienna, Muthgasse 18,1190 Vienna, Austria

[15] BRGM, 3 avenue Claude Guillemin, BP36009, 45060 Orléans Cedex 2, France

[16] Water Research Institute (IRSA), National Research Council (CNR), Via F. De Blasio, 5, 70132 Bari, Italy

[17] Research Institute for Geo-Hydrological Protection (IRPI), National Research Council (CNR), Via Amendola, 122/I, 70126 Bari, Italy

[18] Water, Environmental and Food Chemistry Unit, Institute of Environmental Assessment and Water Research, C/ Jordi Girona 18-26, ES 08034 Barcelona, Spain

[19] Eawag, Swiss Federal Institute of Aquatic Science and Technology, Überlandstrasse 133, 8600 Dübendorf, Switzerland

[20] White Lab Srl, Via Mons. Rodolfi 22, 36022 San Giuseppe de Cassola (VI), Italy

[21] Helmholtz Centre Hereon, Department for Organic Environmental Chemistry, Max-Planck-Str. 1, 21502 Geesthacht, Germany

[22] Het Waterlaboratorium, J.W. Lucasweg 2, 2031 BE Haarlem, The Netherlands

[23] Human exposure to organic pollutants Unit, Institute of Environmental Assessment and Water Research, C/ Jordi Girona 18-26, ES 08034 Barcelona, Spain

[24] Institute for Analytical Research, Hochschulen Fresenius gem. Trägergesellschaft mbH, 65510 Idstein, Germany

[25] Institute for Biodiversity and Ecosystem Dynamics, University of Amsterdam, 1012 WP Amsterdam, Netherlands

[26] Water Research Institute (IRSA), National Research Council of Italy (CNR), via del Mulino, 19, 20861 Brugherio, MB, Italy

[27] Analytical Chemistry Group, Department of plant and environmental sciences, University of Copenhagen, Thorvaldsenvej 40, 1871 Frederiksberg, Denmark

[28] KWR Water Research Institute, Groningenhaven 7, 3433 PE Nieuwegein, The Netherlands

[29] Luxembourg Centre for Systems Biomedicine (LCSB), University of Luxembourg, 6, Avenue du Swing, L-4367 Belvaux, Luxembourg

[30] Center for Omics Sciences (COSR), IRCCS San Raffaele Scientific Institute, 20132 Milan, Italy

[31] Bavarian Environment Agency, Bürgermeister-Ulrich-Str. 160, 86179 Augsburg, Germany

[32] Laboratory for Operation Control and Research, Zweckverband Landeswasserversorgung, Am Spitzigen Berg 1, 89129 Langenau, Germany

[33] NILU, Instituttveien 18, 2007 Kjeller, Norway

[34] US National Institute of Standards and Technology, 331 Fort Johnson Rd, 29412 Charleston, South Carolina, USA

[35] Queensland Alliance for Environmental Health Sciences, The University of Queensland, Woolloongabba, Queensland 4102, Australia

[36] Ministry of Infrastructure and Water Management, Rijkswaterstaat Laboratory, Zuiderwagenplein 2, 8224 AD Lelystad, The Netherlands

[37] SUEZ-CIRSEE, 38 rue du president Wilson, 78230 Le Pecq, France

[38] Universite Claude Bernard Lyon 1, CNRS, ISA, UMR5280, 5 rue de la Doua, F-69100 Villeurbanne, France

[39] Toxicological Centre, University of Antwerp, Universiteitsplein 1, 2610 Antwerp, Belgium

[40] Environmental and Public Health Analytical Chemistry, Research Institute for Pesticides and Water, University Jaume I, 12006 Castelló, Spain

[41] Department of Aquatic Sciences and Assessment, Swedish University of Agricultural Sciences, 75007 Uppsala, Sweden

[42] LEESU, Univ Paris Est Creteil, Ecole des Ponts, F-94010 Creteil, France

[43] Univ Paris Est Creteil, CNRS, OSU-EFLUVE, F-94010, Creteil, France

[44] IBED Environmental Chemistry & Mass Spectrometry Laboratories, University of Amsterdam, Science Park 904, 1098 XH Amsterdam, The Netherlands

[45] VEOLIA Recherche et Innovation, Chemin de la Digue, 78600 Maisons-Laffitte, France

[46] Vlaamse Milieumaatschappij, Raymonde de Larochelaan 1, 9051 Gent, Sint-Denijs-Westerem, Belgium

[47] T. G. Masaryk Water Research Institute, p. r. i., Macharova 5, 70200 Ostrava, Czech Republic

[48] WLN, Rijksstraatweg 85, 9756 AD Glimmen, Groningen, The Netherlands

* Correspondence: anneli.kruve@su.se

TABLE OF CONTENTS


| | | |
|---|---|---|
| SI 1. | Quantification approaches | SI-4 |
| SI 2. | Reported instrumental settings for all participants (anonymized) | SI-5 |
| SI 3. | Reprocessing settings for all raw data (anonymized) | SI-24 |
| SI 4. | Stability | SI-26 |
| SI 5. | Insource degradation of atrazine TPs | SI-27 |
| SI 6. | Peak quality in reprocessed results | SI-28 |
| Fig. S1. | Map of participants geographical position | SI-29 |
| Fig. S2. | Stability in freeze-thaw cycles (-20 °C, thawed each week) | SI-30 |
| Fig. S3. | Stability in fridge (4 °C) | SI-31 |
| Fig. S4. | Stability in room temperature (20 - 25 °C) | SI-32 |
| Fig. S5. | Stability in freezer (-20 °C) | SI-33 |
| Fig. S6. | Prediction errors from sample s1b | SI-34 |
| Fig. S7. | Prediction errors from sample s2a | SI-35 |
| Fig. S8. | Prediction errors from sample s2b | SI-36 |
| Fig. S9. | Prediction errors from sample s3a | SI-37 |
| Fig. S10. | Prediction errors from sample s3b | SI-38 |
| Fig. S11. | Prediction errors for parent-TP approach | SI-39 |
| Fig. S12. | Prediction errors for structural similarity approach | SI-40 |
| Fig. S13. | Prediction errors for close eluting approach | SI-41 |
| Fig. S14. | Prediction errors for RandFor-*IE* approach | SI-42 |
| Fig. S15. | Prediction errors for MLR-*IE* approach | SI-43 |
| Fig. S11. | Occurrences of outliers | SI-44 |
| Fig. S12. | Proportion of close eluting assignments | SI-45 |
| Fig. S13. | Plot of PC1 vs PC3 | SI-46 |
| Fig. S14. | Plot of PC1 vs PC4 | SI-47 |
| | References | SI-48 |

# SI 1. QUANTIFICATION APPROACHES

The three surrogate standard-based quantification approaches use analytical standards that are either structurally similar to the suspect compounds or eluting close to the suspect compounds. Two approaches, the parent-TP approach and the structurally similar approach, are based on the assumption that structurally similar compounds also have similar response factors in ESI/HRMS. Therefore, the calibration curve of a parent compound may be used to quantify TPs[1–4] for which analytical standards can be difficult to obtain. However, the applicability domain for this approach is limited to compounds that may transform, e.g., via degradation or metabolization, and that can be analyzed with the same method. Therefore, in a similar approach, a structurally similar surrogate standard may be used.[5,6] Here, an online platform from the University of Athens[7] was used to find the structurally most similar surrogate standard.[8] The tool compares the SMILES of the suspect compound with the SMILES of compounds from NORMAN SusDat.[9] To identify the target compound with the greatest structural similarity, it calculates the 2D-linear fragment descriptors using the original definitions of atom pairs and atom sequences.[10] The Tanimoto coefficient is used as the measure of similarity distance. Alternatively, the calibration curve of a close eluting analytical standard is applied for the quantification of the suspect compound.[11,12] This approach, proposed by Pieke et al.,[13] assumes that compounds with close retention time will have similar responses in ESI. For these three approaches, the concentration of the suspect/unknown compounds is calculated using the response factor (RF) of the calibration compound according to:

$$c_{\text{suspect compound}} = \frac{\text{peak area}_{\text{suspect compound}}}{\text{RF}_{\text{calibration compound}}} \qquad \text{(Eq. 1)}$$

The two modelling approaches use different machine learning algorithms to predict the *IE* of the suspect compounds: random forest (RandFor) and multiple linear regression (MLR). The first model, developed by Liigand et al.[14] and hereafter called the RandFor-*IE* approach, is based on molecular 2D PaDEL descriptors and five empirical eluent descriptors. It was trained on *IE* values from 1158 compounds measured using different instruments and conditions. Due to the different instrumentation used, all ionization efficiency values were unified during the development of the model. As such, a set of calibration compounds with known concentrations is needed to transform the unified log*IE* values into instrument-specific response factors:

$$\log \text{RF}_{\text{predicted}} = \frac{\log IE_{\text{predicted}}\text{-intercept}}{\text{slope}} \qquad \text{(Eq. 2)}$$

The predicted ionization efficiency is further used to estimate the concentration of the suspect compounds:

$$c_{\text{suspect compound}} = \frac{\text{peak area}_{\text{suspect compound}}}{10^{\log \text{RF}_{\text{predicted}}}} \qquad \text{(Eq. 3)}$$

This approach has been validated in several studies, e.g., on pesticides and mycotoxins in cereals,[15] on micropollutants in ground- and surface waters,[2,16,17] on pharmaceuticals, pesticides, and TPs in river and wastewater,[18] and on per- and polyfluoroalkyl substances in marine mammals (after retraining of the model).[19]

An alternative model was developed by Aalizadeh et al.[20] and will be referred to as the MLR-*IE* approach in this work. The model was trained and tested with 103 emerging contaminants and uses seven molecular descriptors for log*IE* prediction. In addition, this approach normalizes the *IE* values of analytes by dividing them into the baseline *IE* value of a reference chemical (dichlorvos) (Eq. 4) and concentration estimation (Eq. 5) of the suspects.

$$\log IE = \left(\frac{\text{slope}_{\text{compound}} \times \text{MW}_{\text{dichlorvos}}}{\text{slope}_{\text{dichlorvos}} \times \text{MW}_{\text{compound}}}\right) \qquad \text{(Eq. 4)}$$

Moreover, this approach requires atrazine-d5 when establishing a calibration curve for the compounds, to normalize the peak areas from sequence to compensate for sensitivity loss over time as well as the matrix effect. Finally, the concentration of unknown compounds can be predicted via Eq. 5.

$$c_{\text{predicted}} = \frac{\left(\frac{\text{peak area}_{\text{compound}}}{\text{peak area}_{\text{atrazine-d5}}}\right) \times \text{MW}_{\text{dichlorvos}}}{\left[10^{\log IE_{\text{experimental/predicted}}}\right] \times \text{slope}_{\text{dichlorvos}} \times \text{MW}_{\text{compound}}} \qquad \text{(Eq. 5)}$$

## SI 2. REPORTED INSTRUMENTAL SETTINGS FOR ALL PARTICIPANTS (ANONYMIZED)

### Instrumental summary across laboratories/institutes

The samples were analyzed on various LC/ESI/HRMS instrumentation from five LC vendors (Agilent Technologies Inc. (Santa Clara, CA, USA), Sciex Pte. Ltd. (Framingham, MA, USA), Shimadzu Corporation (Tokyo, Japan), Thermo Fisher Scientific™ (Bremen, Germany), Waters Corporation (Wilmslow, UK)) and five MS vendors (Agilent Technologies Inc., Bruker Daltonics (Bremen, Germany), Sciex Pte. Ltd., Thermo Fisher Scientific™, Waters Corporation). All laboratories used reversed-phase chromatography, except one that used a hyphenated reversed phase – hydrophilic interaction liquid chromatography (HILIC) separation. Most laboratories used $C_{18}$ columns or equivalent; however, one laboratory used a $C_6$-phenyl column, one used a biphenyl column, and one combined a $C_{18}$ and ZIC-HILIC column.

Mostly, mobile phase A consisted of formic acid (0.01 – 0.1%) in $H_2O$, sometimes with ammonium acetate, ammonium formate, or ammonium fluoride additive (0.2 – 10 mM). In some cases, a small percentage of organic solvent (acetonitrile (MeCN) or methanol (MeOH), 0.1 – 10 %) was added. The pH of the aqueous mobile phases ranged from 2 to 6.75. In the case of HILIC separation, pure $H_2O$ was used as mobile phase A. Mobile phase B was usually formic acid (0.01 – 0.1%) in MeCN or MeOH, sometimes with ammonium formate or ammonium acetate additive (0.5 – 10 mM). A small percentage of $H_2O$ (2 – 10%) was added in some cases, and sometimes pure MeCN or MeOH was used. One laboratory also used 15% isopropanol in mobile phase B, and another had isopropanol as mobile phase C. Flow rates varied from 150 to 500 μL/min for most laboratories, except for one that used a nano-flow of 0.3 μL/min and three laboratories that varied the flow rate throughout the elution. Injection volumes varied between 1 and 250 μL, column oven temperatures varied between 25 and 45 °C, sampler temperatures were from 4 to 25 °C, and the length of the gradient program varied from 15 to 45 min.

All samples were measured in positive ESI mode. For laboratories using Agilent Technologies Inc. Time-of-Flight (ToF) HRMS, the $MS^1$ settings ranged as follows: scan rates were 2.5 – 6 spectra/s, gas temperatures were 250 – 300 °C, gas flow rates were 9 – 12 L/min, nebulizer pressures were 35 – 40 psi, sheath gas temperatures were 300 – 350 °C, nozzle voltages were 500 V, fragmentor voltages were 120 – 340 V, Vcap voltages were 3.0 – 3.5 kV, skimmer1 were set to 65, and octopole RF peaks were 750.

Laboratories using Bruker Daltonics ToF HRMS used the following $MS^1$ settings: capillary voltages were 3.6 – 4.5 kV, end plate offset voltages were 500 V, dry gas temperatures were 200 – 220 °C, dry gas flow rates were 9 – 11 L/min, nebulizer pressures were 3 bar, funnel RF was set to 200, and multipole RF was set to 50.

Laboratories that used Sciex Pte. Ltd. ToF HRMS had these $MS^1$ settings: spray voltages were 5 – 5.5 kV, capillary temperatures were 500 – 550 °C, ion source gas 1 pressures were 35 – 90 psi, ion source gas 2 pressures were 45 – 60 psi, curtain gas pressures were 20 – 35 psi, and CAD gas was set to 7.

For laboratories using Thermo Fisher Scientific™ Orbitrap HRMS, the $MS^1$ settings used were: resolutions were 30 000 – 240 000, AGC targets were $2 \times 10^5$ – $3 \times 10^6$, maximum IT were 50 – 500 ms, spray voltages were 1.6 – 4.0 kV, spray currents were 0.11 – 100 μA, capillary temperatures were 250 – 350 °C, aux gas heater temperatures were 250 – 400 °C, and S-lens RF-levels were 40 – 70%. The gas flow rates (unitless) ranged from 0 – 50 for sheath gas, 0.04 – 18 for aux gas, and 0 – 10 for sweep gas.

The laboratories that used Waters Corporation ToF HRMS had $MS^1$ settings ranging as follows: capillary voltages were 0.5 – 2.6 kV, source temperatures were 120 – 150 °C, desolvation temperatures were 250 – 600 °C, cone gas flows were 0 – 500 L/h, and desolvation flows were 500 – 1000 L/h.

The data was mostly processed using vendor software, e.g., MassHunter (Agilent Technologies Inc.), DataAnalysis or Target Analysis for Screening and Quantification (Bruker Daltonics), Sciex OS (Sciex Pte. Ltd.), Compound Discoverer, TraceFinder or Xcalibur (Thermo Fisher Scientific™), or MassLynx or UNIFI Scientific Information System (Waters Corporation). For non-vendor software, Skyline (University of Washington) was used. The peaks were mostly detected based on exact ion mass with tolerances spanning from 2 to 20 ppm or 0.0001 to 0.4 Da. Sometimes, RT tolerances (± 0.1 - 0.5 min) or other filters like isotopic fit were applied, followed by manual inspection in some cases.

### DS_AF

*Instrumental settings.* The samples were analyzed using an Acquity I-class UPLC system (Waters Corporation, Wilmslow, UK) connected to a Vion IMS Q-ToF HRMS (Waters Corporation).

The analytes were separated using a CORTECS $C_{18}$, 2.7 μm, 100 × 2.1 mm column (Waters Corporation). 0.01% formic acid in water with pH 3.2 was used as mobile phase A, and mobile phase B was MeOH with 0.01% formic acid. The gradient program started with 10% B and was increased to 90% B over 14 minutes, where it was held for 2 minutes before decreasing back to 10% B over 0.1 minutes. The column was equilibrated for 2 minutes between each injection. The flow rate was 0.3 mL/min, injection volume was 5 μL, sampler temperature was 10 °C and column oven temperature was 40 °C.

All samples were analyzed in positive ESI mode using a scan range of *m/z* 50.0000 – 1000.0000, the resolution was 24 000 at full width half maximum (FWHM) at *m/z* 556.27658, and scan time was 0.3 s. The capillary was set at 0.7 kV, reference capillary was set at 0.8 kV, the cone voltage was 40 V, source temperature was 120 °C, and desolvation temperature was 550 °C. Gas parameters were cone gas flow: 250 L/h and desolvation gas flow: 1000 L/h. MS data was acquired in $HRMS^e$ mode with $N_2$ as the drift gas, and IMS wave velocity of 250 m $s^{-1}$, and wave height of 20-50 V. Leucine enkephalin (*m/z* 556.27658) was used for mass correction. In $HRMS^e$, two independent scans with different collision energies were acquired: a collision energy of 6 eV for the low energy (LE) with a focus on protonated molecules, and a ramp of 28-59 eV for high energy (HE) function focusing on fragmented molecules. Nitrogen was used as a collision-induced dissociation gas.

*Processing details.* The data was processed using UNIFI™ Scientific Information System software (version 1.9.4, Waters Corporation). Peak detection was carried out based on 4-D data (retention time, drift time, *m/z* and intensity) with a threshold for intensity at 200 and 100 ion counts in LE and HE, respectively. Features were automatedly screened for suspected structures with target and fragment match tolerance 5 mDa. Retention time or collision cross section tolerances were not applied.

## DS_AW

*Instrumental settings.* The samples were analyzed on a Nexera LC system (Shimadzu Corporation, Tokyo, Japan) connected to an X500R Q-ToF HRMS (Sciex Pte. Ltd., Framingham, MA, USA.) equipped by an electrospray ionization (ESI) source and a HTS Pal autosampler (CTC Analytics).

The analytes were separated using a $C_{18}$, 100 Å, 5 μm, 100 × 3.0 mm column (Phenomenex®). 0.1% formic acid in water with pH 2.7 was used as mobile phase A, and mobile phase B was MeCN with 0.1% formic acid. The gradient program started with 5% B and increased to 100% B in 15 minutes before decreasing back to 5% B over 2 minutes. The flow rate was 0.4 mL/min, injection volume was 50 μL, sampler temperature was 10 °C and column oven temperature was 40 °C.

All samples were analyzed in positive ESI mode with an acquisition method based on double experiments, i.e. full-scan survey ToF-MS and IDA (Information Dependent Acquisition) experiment in a scan range of 50.0000 – 800.0000 *m/z*, either in ToF-MS with a resolution set to 35 595 at 237.10 *m/z*. Accumulation time was 0.250 s for ToF-MS and 0.07 s for IDA scan; declustering potential was 50 V, collision energy was 10 V, collision energy spread was 0 V, spray voltage was 5.5 kV, and capillary temperature was 550 °C. Ion source gas pressure 1 was 55 psi, ion source gas pressure 2 was 55 psi, curtain gas pressure was 20 psi, and CAD gas 7 psi. The maximum number of candidate ions to monitor per cycle during IDA experiments was set to 7, for ions exceeding a peak intensity threshold of 100 cps; the mass tolerance was set to 50 mDa and dynamic background subtraction was activated.

The mass spectrometer was calibrated automatically using standards recommended by AB SCIEX for calibrating the AB SCIEX X500R® Instrument by means of a Calibrant Delivery System (CDS), every 4 samples inserted into the queue of submitted samples.

*Processing details.* The data was processed using Sciex OS software (version 2.0.1, AB SCIEX™). The peaks were detected and integrated based on mass accuracy error (< 2 ppm), plausible retention time in the chromatogram, isotopic fit (>90%) and the presence of a fragmentation MS/MS pattern.

In detail, as for the peak integration the following parameters were used: XIC width 0.02 Da, RT half window 30.0 sec, minimum peak width 3 points, noise percentage 40%.

## DS_AWW

*Instrumental settings.* The samples were analyzed on a Nexera X2 LC system (Shimadzu Corporation) connected to an X500R Q-ToF HRMS (Sciex Pte. Ltd.).

The analytes were separated using an Acquity UPLC HSS T3 $C_{18}$, 1.8 µm, 50 × 2.1 mm column (Waters Corporation). Mobile phase A was water with 5% MeOH and 5 mM ammonium formate with pH 3, and mobile phase B was MeOH with 5% water and 5 mM ammonium formate. The gradient program started with 0% B for 2 minutes, and increased to 100% B in 12 minutes where it was held for 2 minutes before decreasing back to 0% B over 0.1 minute. The column was equilibrated for 4 minutes between each injection. The flow rate was 0.5 mL/min, injection volume was 25 µL, sampler temperature was 15 °C and column oven temperature was 40 °C.

All samples were analyzed in positive ESI mode with the scan range of 60.0000 – 1500.0000 *m/z* and resolution set to 40 000 at 400 *m/z*. Accumulation time was 0.25 s, declustering potential was 50 V, declustering potential spread was 30 V, collision energy was 10 V, collision energy spread was 0 V, spray voltage was 5.5 kV, and capillary temperature was 550 °C. Ion source gas pressure 1 was 45 psi, ion source gas pressure 2 was 45 psi, curtain gas pressure was 35 psi, and CAD gas was 7 AU.

For $MS^2$, SWATH-MS, a specific variant of data-independent acquisition methods was used. During these measurements, all ionized molecules in the sample that fall within the mass range of 70 and 1500 Da were fragmented using sequential isolated windows. During $MS^2$, declustering potential was 50 V, declustering potential spread was 30 V, collision energy was 50 V and collision energy spread was 30 V.

*Processing details.* The data was processed using Sciex OS software (version 1.6.10.40973, Sciex Pte. Ltd.). Exact ion masses were calculated based on the chemical formula of the substances. The peaks were identified based in accordance with the theoretical exact ion mass (within a mass tolerance range of 5 ppm). Stability of the retention times throughout the whole batch was checked in every single measurement for all substances. Extracted ion chromatogram extraction widths were set around 50-75 ppm in order to improve peak intensities and achieve better sensitivity.

## DS_BIJ

*Instrumental settings.* The samples were analyzed on an Acquity UPLC system (Waters Corporation) connected to a Q Exactive Orbitrap HRMS (Thermo Fisher Scientific™, Bremen, Germany).

The analytes were separated using a Purospher STAR RP-18 endcapped $C_{18}$, 2 µm, 150 × 2.1 mm column (Merck). Mobile phase A was water with 0.1% formic acid with pH 2.7, and mobile phase B was MeCN with 0.1% formic acid. The gradient program started with 5% B for 1 minute and increased to 97% B over 17 minutes, where it was held for 2.5 minutes before decreasing back to 5% B over 1 minute. The column was equilibrated at 5% B for 3.5 minutes between each injection. The flow rate was 0.3 mL/min, injection volume was 10 µL, sampler temperature was 10 °C and column temperature was 20 °C.

All samples were analyzed in positive ESI mode with the following settings: resolution was 70 000, AGC target was $1\times10^6$, maximum IT was 100 ms, and the scan range was 66.7000 – 1000.0000 *m/z*. Sheath gas flow rate was 40 AU, aux gas flow rate was 10 AU, sweep gas flow rate was 2 AU, spray voltage was 3.0 kV, capillary temperature was 350 °C, S-lens RF level was 60.0%, and the aux gas heater temperature was 300 °C.

The dd-$MS^2$ settings were as follows: resolution was 17 500, AGC target was $1\times10^5$, maximum IT was 50 ms, the loop count was 5 and TopN was 5, the isolation window was 2 *m/z*, and the normalized collision energy was 35 eV.

*Processing details.* The data was processed using Thermo Scientific Xcalibur (version 4.1.31.9). The peaks were detected and integrated based on the exact mass and retention time.

## DS_BQW

*Instrumental settings.* The samples were analyzed using an U3000 RSLC HPG system (Thermo Fisher Scientific™) connected to a Maxis Plus Q-ToF HRMS (Bruker Daltonics, Bremen, Germany).

The analytes were separated using an Intensity solo, $C_{18}$, 1.8 µm, 100 × 2.1 mm column (Bruker Daltonics). 0.1% formic acid in water with pH 2.5 was used as mobile phase A, and mobile phase B was MeCN. The gradient program started with 5% B and increased to 100% B over 15 minutes, where it was held for 2 minutes before decreasing back to 5% B over 0.1 minutes. The column was equilibrated for 5 minutes between each injection. The flow rate was 0.3 mL/min, injection volume was 5 µL, sampler temperature was 5 °C and column oven temperature was 40 °C.

All samples were analyzed in positive ESI mode with the following settings: spectra rate was 1 Hz, resolution was set to 38 000 (at 922 *m/z*), and the scan range was 50.0000 – 1000.0000 *m/z*. The capillary voltage was 3.60 kV, dry gas flow was 9 L/min, nebulizer pressure was 3 bar, end plate offset was 500 V, dry temperature was 200 °C, funnel RF was 200 Vpp and multipole RF was 50 Vpp. A solution of sodium formate acetate (10 mM) injected at the beginning of each run was used to automatically calibrate the data.

*Processing details.* The data was processed using Target Analysis for Screening and Quantification (TASQ) software (version 1.4, Bruker Daltonics). The peaks were detected and integrated based on retention time with a tolerance threshold of +/- 0.3 min, exact mass with a tolerance threshold (+/- 5 ppm) and isotope pattern (correlation between theoretical isotope pattern and experimental isotope pattern expressed by msigma (Bruker Daltonics)).

## DS_DBS

*Instrumental settings.* Samples were analyzed on a Vanquish LC system (Thermo Fisher Scientific™) connected to a Q Exactive Plus Orbitrap HRMS (Thermo Fisher Scientific™).

The analytes were separated on an Acquity UPLC HSS T3, 1.8 μm, 150 × 3.0 mm column (Waters Corporation). 0.1% formic acid in water with pH 2.66 was used as mobile phase A and mobile phase B was MeCN with 0.1% formic acid. The gradient program started with 5% B for 2 minutes and was increased to 70% B over 11 minutes, then to 95% B over 2 minutes and to 100% B over 3.25 minutes. The gradient was held at 100% B for 1.75 minutes before decreasing back to 5% B over 0.5 minutes. The column was equilibrated for 2.5 minutes between each injection. The flow rate was 0.4 mL/min, injection volume was 5 μL, sampler temperature was 8 °C and column oven temperature was 40 °C.

All samples were analyzed in positive ESI mode with the following settings: number of microscans were 1, resolution was set to 70 000, AGC target was $1{\times}10^6$, maximum IT was 100 ms, and the scan range was 70.0000 – 1050.0000 *m/z*. Sheath gas flow rate was 35 AU, aux gas flow rate was 10 AU, sweep gas flow rate was 1 AU, spray voltage was 3.4 kV, spray current was 0,41 μA, capillary temperature was 300 °C, S-lens RF level was 50.0%, and the aux gas heater temperature was 320 °C.

$MS^2$: Top 5 Full scan/dd with inclusion list (do not pick other). Full Scan R-70K- $MS^2$-17.5K

*Processing details.* The data was processed using TraceFinder (version 5.1, Thermo Fisher Scientific™).

## DS_DID

*Instrumental settings.* The samples were analyzed using an Agilent 1290 Infinity II UPLC (Agilent Technologies Inc., Santa Clara, CA, USA) connected to an Agilent 6560 LC-IM-QTOFMS system (Agilent Technologies Inc.).

The analytes were separated using an Acquity UPLC HSS T3, silica-based bonded phase, 1.8 μm, 150 × 2.1 mm column + guard column (Waters Corporation). 0.1% formic acid in water with pH 2.7 was used as mobile phase A, and mobile phase B was MeCN with 0.1% formic acid. The gradient program started with 5% B for 2 minutes, and increased first to 15% B over 8 minutes, then to 50% B for 7 minutes, to 60% B for 3 minutes, to 70% B over 5 min, and to 90% B over 3 min. The gradient was held at 90% B for 3 minutes before decreasing back to 5% B over 2 minutes. The column was equilibrated for 3 minutes between each injection. The flow rate was 0.48 mL/min, injection volume was 5 μL, sampler temperature was 7-8 °C, and column oven temperature was 45 °C.

The samples were analyzed in positive ESI mode with the following settings: scan rate was 2.5 spectra/s, resolving power was approximately 20 000 at *m/z* 322, and scan range was 50.0000 – 1700.0000 *m/z*. The gas temperature was 300 °C, gas flow was 10 L/min, nebulizer was 35 psi, VCap was 3.50 kV, sheath gas temperature was 300 °C, sheath gas flow was 11 L/min, nozzle voltage was 500 V, fragmentor was 340 V, skimmer 1 was 32 V, and Octopole RF Peak was 750 V.

*Processing details.* The data was processed using Agilent Technologies MassHunter Suite (MassHunter Qualitative Analysis 10.0 and MassHunter Quantitative Analysis 12.0). The peaks were detected and integrated based on accurate mass (detected: mass tolerance +/- 5 ppm; integration: +/-10 ppm) and retention time.

## DS_DP

*Instrumental settings.* Samples were analyzed on an Accela LC (Thermo Fisher Scientific™) connected to a Q Exactive HF Orbitrap HRMS (Thermo Fisher Scientific™).

Analytes were separated on Acquity Ultra Performance Liquid Chromatography BEH $C_{18}$ column, 1.7 µm, 150 × 2.1 mm (Waters Corporation). 0.1% formic acid in water with pH 2.7 was used as mobile phase A and mobile phase B was MeOH. The gradient program started with 10% B for 2 minutes and was increased to 100% B over 13 minutes, where it was held for 5 minutes, before decreasing back to 10% B over 0.1 minutes. The column was equilibrated at 10% B for 9.9 minutes between each injection. The flow rate was 0.2 mL/min, injection volume was 5 µL, sampler temperature was 4 °C and column oven temperature was 35 °C.

All samples were analyzed in positive ESI mode with the following settings: number of microscans were set to 1, resolution was set to 120 000 (at 200 *m/z*), AGC target was $5\times10^5$, maximum IT was 70 ms, and the scan range was 60.0000 – 900.0000 *m/z*. Sheath gas flow rate was 45 AU, auxiliary gas flow rate was 10 AU, sweep gas flow rate was 2 AU, spray voltage was 3.5 kV, capillary temperature was 250 °C, S-lens RF level was 70.0%, and the auxiliary gas heater temperature was 400 °C.

For $MS^2$, data-dependent acquisition was performed with the following settings: resolution ($MS^2$ = 30 000 at *m/z* 200), AGC target ($5.0 \times 10^5$), maximum IT 70 ms, loop count (10), TopN (10) and isolation window (1.0 Da).

*Processing details.* The data was processed using Xcalibur™ software (version 4.1, Thermo Fisher Scientific™) and R (version 4.1.2, The R Foundation for Statistical Computing). The peaks were detected and integrated based on the Xcalibur™ templates provided by the NORMAN network.

## DS_DX

*Instrumental settings.* The samples were analyzed on an Agilent 1260 Infinity LC system (Agilent Technologies Inc.) connected to an Orbitrap Exploris 120 HRMS (Thermo Fisher Scientific™).

The analytes were separated using a serial coupling of RP and HILIC. As stationary phases, a Poroshell 120 EC $C_{18}$, 2.7 µm, 50 × 3 mm column (Agilent Technologies Inc.) and a ZIC-HILIC, 5 µm, 150 × 2.1 mm column (Merck) were used. The columns were coupled by a T-piece (Upchurch Scientific) and a second binary pump. The reversed phase mobile phases were A: 10 mM ammonium acetate in $H_2O$:MeCN 90:10 v/v, and B: MeCN: 10 mM ammonium acetate in $H_2O$ 90:10 v/v. The gradient program started with 0% B for 7 minutes, and increased first to 50% B in 5 minutes where it was held for 1 minute, and then increased to 100% B over 9 minutes. The gradient was held at 100% B for 10 minutes before decreasing back to 0% B in 1 minute. Together with the mobile phase gradient, the flow rate was adjusted as follows: 0.05 mL/min for 12 minutes, then increased to 0.1 mL/min for the rest of the gradient.

For HILIC, the mobile phases were C: $H_2O$, and D: MeCN. The gradient started with 100% D for 6 minutes, and was decreased to 60% D over 7 minutes where it was held for 19 minutes before increasing back to 100% D in 1 minute. The gradient was held at 100% D for 21 minutes. The flow rate was also adjusted during the gradient, starting at 0.4 mL/min for 32 minutes and then increased to 0.8 mL/min until the end of gradient.

The injection volume was 10 µL, sampler temperature was 25 °C and column oven temperature was 25 °C.

All samples were analyzed in positive ESI mode with the following settings: number of microscans were 1, the scan range was 70.0000 – 1000.0000 *m/z*, the resolution was set to 60 000, AQC target was set to standard and maximum IT to auto. Sheath gas flow rate was 50 AU, aux gas flow rate was 8 AU, sweep gas flow rate was 0 AU, spray voltage was 3.50 kV, capillary temperature was 320 °C, S-lens RF level was 70.0%, and the aux gas heater temperature was 400 °C.

$MS^2$ experiments were performed with a resolution of 30 000. In each cycle up to four $MS^2$ experiments were conducted, with a dynamic exclusion of seven seconds for a mass after an obtained spectrum.

*Processing details.* The data was processed using TraceFinder 5.1 (Thermo Fisher Scientific™). The peaks were detected and integrated based on the sum formulae.

## DS_EF

*Instrumental settings.* Samples were analyzed on an Accela LC (Thermo Fisher Scientific™) connected to a Q Exactive HF Orbitrap HRMS (Thermo Fisher Scientific™).

Analytes were separated on Acquity Ultra Performance Liquid Chromatography BEH $C_{18}$ column, 1.7 µm, 150 × 2.1 mm (Waters Corporation). 0.1% formic acid in water with pH 2.7 was used as mobile phase A and mobile phase B was MeOH. The gradient program started with 10% B for 2 minutes and was increased to 100% B over 13 minutes, where it was held for 5 minutes, before decreasing back to 10% B over 0.1 minutes. The column was equilibrated at 10% B for 9.9 minutes between each injection. The flow rate was 0.2 mL/min, injection volume was 5 µL, sampler temperature was 4 °C and column oven temperature was 35 °C.

All samples were analyzed in positive ESI mode with the following settings: number of microscans were set to 1, resolution was set to 120 000 (at 200 *m/z*), AGC target was $5\times10^5$, maximum IT was 70 ms, and the scan range was 60.0000 – 900.0000 *m/z*. Sheath gas flow rate was 45 AU, auxiliary gas flow rate was 10 AU, sweep gas flow rate was 2 AU, spray voltage was 3.5 kV, capillary temperature was 250 °C, S-lens RF level was 70.0%, and the auxiliary gas heater temperature was 400 °C.

For $MS^2$, data-dependent acquisition was performed with the following settings: resolution ($MS^2$ = 30,000 at *m/z* 200), AGC target ($5.0 \times 10^5$), maximum IT 70 ms, loop count (10), TopN (10) and isolation window (1.0 Da).

*Processing details.* The data was processed using Xcalibur™ software (version 4.1, Thermo Fisher Scientific™) and R (version 4.1.2, The R Foundation for Statistical Computing). The peaks were detected and integrated based on the Xcalibur™ templates provided by the NORMAN network.

## DS_GJT

*Instrumental settings.* The samples were analyzed on a Exion LC system (Shimadzu Corporation) connected to an X500R Q-ToF HRMS (Sciex Pte. Ltd.).

The analytes were separated using an Eclipse Plus $C_8$, 1.8 µm, 100 × 2.1 mm column (Agilent Technologies Inc.). Mobile phase A was water with 2% MeOH, 0.1% formic acid, and 0.5 mM ammonium formate with pH 6, and mobile phase B was MeOH with 1.9% water, 0.1% formic acid, and 0.5 mM ammonium formate. The gradient program started with 0% B for 1 minute, and increased first to 30% B in 2 minutes, then to 50% B in 3 minutes, and then to 100% B in 10 minutes. The gradient was held at 100% B for 2 minutes before decreasing back to 0% B over 0.1 minute. The column was equilibrated for 2 minutes between each injection. The flow rate was 0.3 mL/min, injection volume was 20 µL, sampler temperature was 15 °C and column oven temperature was 45 °C.

All samples were analyzed in positive ESI mode with the scan range of 50.0000 – 1100.0000 m/z and resolution set of 33 829 at *m/z* 609.2807. Ion source gas pressure 1 was 60 psi, ion source gas pressure 2 was 60 psi, curtain gas pressure was 30 psi, and CAD gas was 7 AU. Ion spray voltage was 5.0 kV and capillary temperature was 550 °C.

For ToF/MS: Accumulation time was 0.05 s, declustering potential was 80 V, declustering potential spread was 0 V, collision energy was 10 V, collision energy spread was 0 V.

For ToF/MSMS: SWATH analysis was used with a total of 10 windows (*m/z* 50 – 150, 149 – 190, 189 – 225, 224 – 260, 259 – 295, 294 – 330, 329 – 365, 364 – 400, 399 – 600, 599 – 1100). Accumulation time was 0.025 s, declustering potential was 80 V, declustering potential spread was 0 V, collision energy was 35 V, collision energy spread was 5 V, spray voltage was 5.0 kV, and capillary temperature was 550 °C.

*Processing details.* The data was processed using SCIEX OS software (version 2.1.0, Sciex Pte. Ltd.). For non-targeted peak picking, the peaks were detected and integrated using 6 out of 7 peak detection sensitivity (with 1 being "fast" and 7 "exhaustive") and no minimum or maximum retention time. Integration parameters were set to RT half window of 9.0 s, minimum peak width of 3 points, minimum peak height of 500, S/N integration threshold of 3, Gaussian Smooth width of 0.8 points, noise percentage of 20 %, baseline subtract window of 0.5 min and peak splitting of 2 points.

For suspect peak picking the same integration parameters were used except for a lower minimum peak height of 200.

## DS_GS

*Instrumental settings.* Samples were analyzed in triplicate on a Vanquish UPLC system (Thermo Fisher Scientific™) connected to a Q Exactive HF Orbitrap (Thermo Fisher Scientific™).

The analytes were separated on a ZORBAX RR Eclipse Plus $C_{18}$, 3.5 µm, 150 × 2.1 mm column (Agilent Technologies Inc.). Mobile phase A was 0.1% formic acid in water with pH 2.7 and mobile phase B was 0.1% formic acid in MeOH. The gradient program started with 2% B for 1 minute and was increased to 98% B over 19 minutes, where it was held for 5 minutes, before decreasing back to 2% B over 0.1 minutes. The column was equilibrated for 5 minutes between each injection. The flow rate was 0.3 mL/min, injection volume was 100 µL, sampler temperature was 10 °C and column oven temperature was 40 °C.

All samples were analyzed in positive ESI mode only using Full scan-vDIA acquisition with these MS settings: number of microscans were 1, $MS^1$ resolution was set to 120 000, AGC target was $1\times10^6$, maximum IT was 240 ms, and the scan range was 70.0000 – 1050.0000 *m/z*. Sheath gas flow rate was 40 AU, aux gas flow rate was 10 AU, sweep gas flow rate was 10 AU, spray voltage was 3.60 kV, capillary temperature was 320 °C, the S-lens RF level was 50.0%, and the aux gas heater temperature was 350 °C.

$MS^2$ data was collected via data independent acquisition (vDIA, $MS^2$ resolution 30 000). However, within this project stage no $MS^2$ data has been used for additional ID or quantification purposes.

*Processing details.* The data was processed using Tracefinder (version 5.0.889.0, Thermo Fisher Scientific™, Bremen, Germany). The peaks were detected and integrated based on exact mass within 5 ppm mass tolerance of the main adduct, e.g. [M+H]+ , additional adducts in case these were visible and retention time. MS2 data based on variable data independent acquisition was collected in addition to full MS data but not used for additional ID or quantification purposes. Retention times, peak intensities (peak area) for given targets were exported for individual measurements and combined for 3 replicates in the project template.

## DS_GSB

*Instrumental settings.* Samples were analyzed on a Vanquish UPLC (Thermo Fisher Scientific™) connected to a Fusion Lumos Orbitrap (Thermo Fisher Scientific™).

Analytes were separated on an Acquity HSS T3 $C_{18}$, 1.8 µm, 150 × 2.1 mm column (Waters Corporation). 0.1% formic acid in water with pH 2.7 was used as mobile phase A and mobile phase B was MeOH with 0.1% formic acid. The gradient program started with 2% B for 1 minute and was increased to 98% B over 15 minutes, where it was held for 5 minutes, before decreasing back to 2% B over 1 minute. The column was equilibrated for 6 minutes between each injection. The flow rate was 0.3 mL/min, injection volume was 10 µL, sampler temperature was 10 °C and column oven temperature was 35 °C.

All samples were analyzed in positive ESI mode with the following settings: number of microscans were 1, resolution was set to 120 000, AGC target was $5\times10^5$, maximum IT was 50 ms, and the scan range was 70.0000 – 1450.0000 *m/z*. Sheath gas flow rate was 50 AU, aux gas flow rate was 10 AU, sweep gas flow rate was 1 AU, spray voltage was 3.50 kV, capillary temperature was 350 °C, ion transfer tube temperature 325 °C, and the S-lens RF level was 60.0%.

An additional data dependent analysis was performed using TopN, (1 s cycle time) with a dynamic exclusion of 10 s (with 10 ppm error). Full scan resolution using the orbitrap was set as described above. Data-dependent fragmentation was performed using higher-energy collisional dissociation (HCD) with a stepped collision energy of 20, 35, and 50 with quadrupole isolation at *m/z* 1.5 width. The fragment scan resolution using the orbitrap was set at 15 000, ion target value of $5.0 \times 10^3$ and 22 ms maximum injection time.

*Processing details.* The data was processed using Skyline software (21.2.0.369, University of Washington). The peaks were detected and integrated based on retention time (± 0.5 min), mass tolerance (0.0001 *m/z*), and $MS^1$ filtering of 120 000 @ 200 *m/z*.

## DS_GSW

*Instrumental settings.* The samples were analyzed on a 1290 Infinity II UHPLC system (Agilent Technologies Inc.) connected to an X500R Q-ToF HRMS (Sciex Pte. Ltd.).

The analytes were separated using an Arion Plus $C_{18}$, 3 µm, 100 × 2.1 mm column (Chromservis s.r.o.). Mobile phase A was water with 5 mM ammonium formate with pH 6.5, and mobile phase B was MeOH. The gradient program started with 5% B for 0.5 minute, and increased first to 40% B in 2.5 minutes, then to 95% B in 11 minutes. The gradient was held at 95% B for 4 minutes before decreasing back to 5% B over 0.1 minute. The column was equilibrated for 4 minutes between each injection. The flow rate was 0.2 mL/min, injection volume was 20 µL, sampler temperature was 20 °C and column oven temperature was 30 °C.

All samples were analyzed in positive ESI mode with the scan range of 80.0000 – 1500.0000 *m/z* and resolution set to 26 749 at 132.9049 *m/z* and 36 104 at 829.5397 *m/z*. Accumulation time was 0.15 s, declustering potential was 80 V, declustering potential spread was 0 V, collision energy was 10 V, collision energy spread was 0 V, spray voltage was 5.5 kV, and capillary temperature was 500 °C. Ion source gas pressure 1 was 40 psi, ion source gas pressure 2 was 50 psi, curtain gas pressure was 35 psi, and CAD gas was 7 (without a unit, range is 0 - 15).

Scan range for $MS^2$ was 40.0000 – 1500.0000 *m/z* and resolution set to 28 828 at 185.1286 *m/z*, 34 532 at 298.2124 *m/z*, 36 925 at 494,3336 *m/z* and 37 212 at 607.4183 *m/z*. Accumulation time was 0.05 s, declustering potential was 80 V, declustering potential spread was 0 V, collision energy was 35 V, collision energy spread was 15 V, spray voltage was 5.5 kV, and capillary temperature was 500 °C. Ion source gas pressure 1 was 40 psi, ion source gas pressure 2 was 50 psi, curtain gas pressure was 35 psi, and CAD gas was 7 (without a unit, range is 0 - 15).

*Processing details.* The data was processed using SCIEX OS software (version 1.7.0.36606, Sciex Pte. Ltd.). The peaks were detected and integrated based on the auto-peak algorithm (minimum peak height was 100.00, minimum signal/noise was 2.00, precursor mass tolerance was ± 0.4 Da).

## DS_HT

*Instrumental settings.* Samples were analyzed on a Dionex UltiMate™ 3000 UHPLC system (Thermo Fisher Scientific™) connected to a Q Exactive Orbitrap HRMS (Thermo Fisher Scientific™).

The analytes were separated on a ZORBAX Eclipse Plus $C_{18}$, 3.5 µm, 150 × 2.1 mm column (Agilent Technologies Inc.). 0.1% formic acid in water with pH 2.6 was used as mobile phase A and mobile phase B was MeCN with 0.1% formic acid. The gradient program started with 1% B for 1 minute and was increased to 20% B over 1 minute, then to 98% B over 14.5 minutes. The gradient was held at 98% B for 5.5 minutes, before decreasing back to 1% B over 0.1 minutes. The column was equilibrated at 1% B for 4.9 minutes between each injection. The flow rate was 0.3 mL/min, injection volume was 50 µL, sampler temperature was 5 °C and column oven temperature was 35 °C.

All samples were analyzed in positive ESI mode with the following settings: number of microscans were 1, resolution was set to 140 000, AGC target was $1\times10^6$, maximum IT was 400 ms, and the scan range was *m/z* 100.0000 – 1050.0000. Sheath gas flow rate was 42 AU, aux gas flow rate was 12 AU, sweep gas flow rate was 2 AU, spray voltage was 3.5 kV, spray current was 100 µA, capillary temperature was 300 °C, S-lens RF level was 60.0%, and the aux gas heater temperature was 350 °C.

Profile type product ion spectra were recorded in DDA-mode at a resolution of 17 500 of the TopN 5 precursor *m/z* applying stepped collision energies of 20, 40, 60. The AGC target was set to $2\times10^5$, the maximum IT to 50 ms, $MS^X$ count to 1 and the loop count to 5. The isolation window was 0.8 u without an isolation offset and the fixed first mass of *m/z* 50.

*Processing details.* The data was processed using TraceFinder 5.1 (Thermo Fisher Scientific). The peaks were detected and integrated based on retention time and exact ion mass of the precursor ion with a mass tolerance of 5 ppm. Peak detection/integration was carried out applying TraceFinders Genesis detection algorithm and a peak smoothing factor of 5.

## DS_ITG

*Instrumental settings.* Samples were analyzed on a Vanquish UPLC (Thermo Fisher Scientific™) connected to a Tribrid Orbitrap Fusion HRMS (Thermo Fisher Scientific™).

Analytes were separated on an XBridge BEH $C_{18}$ XP, 2.5 µm, 150 × 2.1 mm column (Waters Corporation). 0.05% formic acid in ultrapure water with pH 2.9 was used as mobile phase A and mobile phase B was MeCN with 0.05% formic acid. The gradient program started with 5% B for 1 minute and was increased to 95% B over 24 minutes, where it was held for 4 minutes, before decreasing back to 5% B over 0.5 minutes. The column was equilibrated for 4.5 minutes between each injection. The flow rate was 0.25 mL/min, injection volume was 10 µL, sampler temperature was 15 °C and column oven temperature was 25 °C.

All samples were analyzed in positive ESI mode with the following settings: number of microscans were 1, resolution was set to 120 000, AGC target was $2\times10^5$, maximum IT was 100 ms, and the scan range was 80.0000 – 1500.0000 *m/z*. Sheath gas flow rate was 40 AU, aux gas flow rate was 10 AU, sweep gas flow rate was 5 AU, spray voltage was 3.00 kV, spray current was circa 11 µA (at the start of the run), capillary temperature was 300 °C, S-lens RF level was 50.0%, and the aux gas heater temperature was 300 °C.

Data-dependent acquisition was performed using a high-collision dissociation (HCD) energy at 20, 35 and 50% (stepped). The resolution was set to 15 000, AGC target was $5\times10^4$, and maximum IT was 60 ms and scan range was set to automatic.

*Processing details.* The data was processed using Xcalibur (Thermo Fisher Scientific™) version 4.2.28.14. The peaks were detected and integrated based on peak area using exact ion mass with a mass tolerance of 15 ppm and a retention time window of 30 s was used (manual check afterwards). The ICIS peak integration settings were set at: smoothing points 1, baseline window 20, area noise factor 5 and peak noise factor 10.

## DS_JBB

*Instrumental settings.* The samples were analyzed using an Agilent 1290 Infinity II UHPLC system (Agilent Technologies Inc.) connected to an Agilent 6546 Q-ToF HRMS (Agilent Technologies Inc.). The Agilent UHPLC system was equipped with a 1290 multisampler (G7167B), a 1290 binary pump (G7120A), a 1260 isopump (G7110B) and a 1290 MCT column compartment (G7116B). For ionization, an Agilent jet stream electrospray ionization source was used (Dual AJS ESI, G1958-65271).

The analytes were separated using a ZORBAX Eclipse Plus $C_{18}$, 95 Å, 1.8 µm, 100 × 2.1 mm column (Agilent Technologies Inc.). 0.1% formic acid in water with pH 3.0 was used as mobile phase A, and mobile phase B was MeCN. The gradient program started with 5% B for 0.5 minutes, increased to 100% B over 15 minutes where it was held for 3 minutes before decreasing back to 5% B over 0.1 minutes. The column was equilibrated for 2 minutes between each injection. The flow rate was 0.3 mL/min, injection volume was 5 µL, sampler temperature was 7 °C and column oven temperature was 40 °C.

The samples were analyzed in positive ESI mode with the following settings: scan rate was 6 spectra/s, and scan range was 100.0000 – 1700.0000 *m/z*. The instrument mode was set to standard resolution (data acquisition at 10 GHz, resolution of ~ 40 000 at *m/z* 118, 50 000 at *m/z* 322 and greater than 60 000 at *m/z* 622, 922, 1221 and 1521), The gas temperature was 250 °C, gas flow was 12 L/min, nebulizer was 40 psig, VCap was 3.50 kV, sheath gas temperature was 350 °C, sheath gas flow was 11 L/min, nozzle voltage was 0 V, fragmentor was 120 V, skimmer 1 voltage was 65 V and the peak-to-peak octopole RF voltage was 750 V.

The Q-ToF HRMS instrument was operated in All Ions MS/MS acquisition mode with three discrete collision energies (0, 10, 40 V).

*Processing details.* The data was processed using MassHunter software (version 10.0, Agilent Technologies Inc.). Using the Personal Compound Database and Library (PCDL) manager software (version B.08.00, Agilent Technologies Inc.), a compound database containing the calibrants and suspects was tailored. In the MassHunter software (version 10.0, Agilent Technologies Inc.), this PCDL was taken as the source of compound formulas for the "Find by Formula" (FBF) data mining algorithm. As formula matching parameters, the mass match tolerance was set to +/- 10 ppm and the retention time tolerance to +/- 0.35 minutes. The extracted ion chromatograms (EICs) were integrated using the Agile 2 Integrator.

## DS_JBQ

*Instrumental settings.* The samples were analyzed using an U3000 RSLC HPG system (Thermo Fisher Scientific™) connected to a Maxis plus Q-ToF (Bruker Daltonics).

The analytes were separated using an Intensity solo, $C_{18}$, 1.8 µm, 100 × 2.1 mm column (Bruker Daltonics). Mobile phase A was 99:1 $H_2O$:MeOH mixture with 5 mM formate ammonium and 0.1% formic acid, pH 3.9, and mobile phase B was MeOH with 5mM formate ammonium and 0.1% formic acid. The gradient started at 4% B, and was first increased to 18.3% B over 1 minute, then to 50% B over 1.5 minutes, then to 99.9% B over 11.5 minutes. The gradient was held at 99.9% B for 2 minutes before decreasing back to 4% B over 0.1 minutes. The gradient was held at 4% B for 4 minutes in between injections. Together with the gradient, the flow rate was varied as follows: started at 0.2 mL/min for 1 minute, increased to 0.233 mL/min over 1.5 minutes, then increased to 0.4 mL/min over 11.5 minutes, then increased to 0.48 mL/min over 2 minutes where it was held for 3 minutes

before decreasing back to 0.2 mL/min over 1 minute. The injection volume was 5 μL, sampler temperature was 5 °C and column oven temperature was 40 °C.

All samples were analyzed in positive ESI mode with the following settings: spectra rate was 1 Hz, resolution was set to 38 000 (at 922 *m/z*), and the scan range was 50.0000 – 1000.0000 *m/z*. Capillary voltage was 3.60 kV, dry gas flow was 9 L/min, nebulizer pressure was 3 bar, end plate offset was 500 V, dry temperature was 200 °C, funnel RF was 200 Vpp and multipole RF was 50 Vpp. A solution of sodium formate acetate (10mM) injected at the beginning of each run was used to automatically calibrate the data.

*Processing details.* The data was processed using Target Analysis for Screening and Quantification (TASQ) software (version 1.4, Bruker Daltonics). The peaks were detected and integrated based on retention time with a tolerance threshold of +/- 0.3 min, exact mass with a tolerance threshold (+/- 5 ppm) and isotope pattern (correlation between theoretical isotope pattern and experimental isotope pattern expressed by msigma (Bruker Daltonics)).

## DS_JDF

*Instrumental settings.* Samples were analyzed on a Dionex UltiMate™ 3000 UHPLC system (Thermo Fisher Scientific™) connected to a Q Exactive Orbitrap (Thermo Fisher Scientific™).

The analytes were separated on a Hypersil GOLD aQ $C_{18}$, 1.9 μm, 100 × 2.1 mm column (Thermo Fisher Scientific™). Mobile phase A was 0.1% formic acid in water with pH 2.6 and mobile phase B was 0.1% formic acid in MeOH. The gradient program started with 0% B for 1 minute and was increased to 100% B over 7 minutes, was held at 100% B for 4 minutes, before decreasing back to 0% B over 1 minute. The column was equilibrated at 0% B for 6 minutes between each injection. The flow rate was 0.3 mL/min, injection volume was 20 μL, sampler temperature was 10 °C and column oven temperature was 30 °C.

All samples were analyzed in positive ESI mode and the detection mode used is full MS with data dependent $MS^2$ (full MS/dd$MS^2$) without inclusion and exclusion list. The settings of the Full MS were: number of microscans were 1, resolution was set to 70 000, AGC target was $1\times10^6$, maximum IT was 200 ms, and the scan range was 70.0000 – 1000.0000 *m/z*. Sheath gas flow rate was 35 AU, aux gas flow rate was 10 AU, sweep gas flow rate was 0 AU, spray voltage was 3.5 kV, capillary temperature was 320 °C, the S-lens RF level was 60.0%, and aux gas heater temperature was 350 °C.

The settings of the data dependent $MS^2$ were: number of microscans were 1, resolution was set to 17 500, AGC target was $1\times10^5$, maximum IT was 50 ms, the loop count was 5, the $MS^X$ count was 1 so the TopN was 5, the isolation window was 2 *m/z*, the fixed first mass was 50 *m/z*, the Normalized Collision Energy (NCE) was 35, 50, 65 and the minimum AGC target was $5.00e^3$.

*Processing details.* The data was processed using Xcalibur software (version 2.2, Thermo Fisher Scientific™). The peaks were detected and integrated based on the retention time criteria, and exact ion mass with a mass tolerance of 10 ppm.

## DS_JL

*Instrumental settings.* The samples were analyzed on a Vanquish LC system (Thermo Fisher Scientific™) connected to a Q Exactive Orbitrap HRMS (Thermo Fisher Scientific™).

The analytes were separated using a $C_6$-phenyl, 3 μm, 150 × 3.0 mm column (Phenomenex®). Mobile phase A was water with 0.025% formic acid and 5 mM ammonium formate with pH 3.0, and mobile phase B was 85% MeOH with 15% isopropanol, 0.025% formic acid and 5 mM ammonium formate. The gradient program started with 8% B for 2.5 minutes and increased to 98% B over 19 minutes, where it was held for 6.5 minutes before decreasing back to 8% B over 0.1 minutes. The column was equilibrated at 8% B for 5 minutes between each injection. The flow rate was 0.4 mL/min, injection volume was 70 μL, sampler temperature was 4 °C and column oven temperature was 35 °C.

All samples were analyzed in positive ESI mode with the following settings: number of microscans were 1, resolution was 70 000, AGC target was $3\times10^6$, maximum IT was 100 ms, and the scan range was 150.0000 – 1500.0000 *m/z*. Sheath gas flow rate was 35 AU, aux gas flow rate was 18 AU, sweep gas flow rate was 0 AU, spray voltage was 4.0 kV, spray current was approximately 6 μA, capillary temperature was 300 °C, S-lens RF level was 50.0%, and the aux gas heater temperature was 30 °C.

*Processing details.* Data were processed using Xcalibur™ software (2.6, Thermo Fisher Scientific™). The peaks were detected and integrated using the NORMAN processing method provided by the organizers of the trial (no parameters were changed),

adjusting the retention time considering the in-house chromatographic conditions. All peak areas were exported to an Excel file using the Xcalibur™ Quan file function and subsequently processed according to the indications provided in the trial.

## DS_JSG

*Instrumental settings.* The samples were analyzed on a Thermo Ultimate 3000 LC system (Thermo Fisher Scientific™) connected to a Bruker Impact II Q-ToF HRMS (Bruker Daltonics).

The analytes were separated using an Acclaim RSLC 120 $C_{18}$ 2.2 µm, 100 × 2.1 mm column (Thermo Fisher Scientific) after passing an Acquity UPLC BEH $C_{18}$ 1.7 µm, 5 × 2.1 mm VanGuard pre-column. Mobile phase A was ultrapure water/methanol (90/10 v/v-%) with 5 mmol/L ammonium formate buffer (pH is 4.3), and mobile phase B was methanol with 5 mmol/L ammonium formate buffer. The gradient program started with 1% B at 0.2 mL/min, and increased to 100% B at 0.48 mL/min at 14 minutes, where it was held for 1.5 minutes at 0.48 mL/min before decreasing back to 1% B and 0.2 mL/min in 0.1 minutes. The column was equilibrated at gradient starting composition for 3.5 minutes between each injection. The injection volume was 250 µL, sampler temperature was 6 °C and column oven temperature was 30 °C.

All samples were analyzed in positive ESI mode with the following settings: spectra rate was 8 spectra/s, resolution was 35 000 at 922 *m/z*, and the scan range was 20.0000 – 1 300.0000 *m/z*. The capillary voltage was 4.5 kV, spray current was 2.5 µA, dry gas flow was 8 L/min, nebulizer pressure was 2 bar, end plate offset was 500 V, dry temperature was 200 °C, funnel RF was 200 Vpp, and multipole RF was 50 Vpp.

$MS^2$ experiments were performed using Auto MS/MS with a cycle time of 1.0 s. Thresholds were set absolute at 1 500 counts, and selected precursors were excluded for 0.3 min after 3 spectra were obtained. MS/MS spectra were obtained at 6 spectra/s. Collision experiments were performed at 20V.

## DS_JWW

*Instrumental.* The samples were analyzed on a Dionex UltiMate™ 3000 UHPLC system (Thermo Fisher Scientific™) connected to a Q Exactive HF Orbitrap HRMS (Thermo Fisher Scientific™).

The analytes were separated using an Easy-spray PepMap RSLC $C_{18}$, 100 Å, 2 µm, 150 mm × 50 µm, column (Thermo Scientific™). Water with 2% MeCN and 0.1% formic acid with pH 2.7 was used as mobile phase A, and mobile phase B was 98% MeCN with 0.1% formic acid. The gradient program started with 10% B for 2 minutes and increased to 95% B over 15 minutes, where it was held for 10 minutes before decreasing back to 10% B over 0.5 minutes. The column was equilibrated for 12.5 minutes between each injection. The flow rate was 300 nL/min, injection volume was 1 µL, sampler temperature was 8 °C and column oven temperature was 40 °C.

All samples were analyzed in positive ESI mode with the following settings: number of microscans were 1, resolution was set to 240 000, AGC target was $1\times10^6$, maximum IT was 200 ms, and the scan range was 70.0000 – 1050.0000 *m/z*. Sheath gas flow rate was 0 AU, aux gas flow rate was 0.04 AU, sweep gas flow rate was 0.02 AU, spray voltage was 1.60 kV, spray current was 0.11 µA, capillary temperature was 250 °C, and the S-lens RF level was 50.0%.

*Processing details.* The data processing including peak detection, retention time alignment, peak integration, and peak picking was performed using Compound Discoverer 3.3 (Version 3.3.1.111, Thermo Scientific™). Peaks were extracted from raw data followed by retention time alignment with adaptive curve settings of 5 ppm and 0.5 min mass and retention time tolerance, respectively. Then the target compounds were detected with 5 ppm mass and 0.3 min retention time tolerance, intensity threshold of $10^6$ and then grouped with the same mass and retention time tolerances. All sample data were aligned with the blanks to link the same features across the samples. For blank correction, the field/procedural blank samples were used as system blanks. Then, peaks were excluded if the sample-to-blank intensity ratio was less than 10.

## DS_MT

*Instrumental settings.* The samples were analyzed using an Acquity UPLC system (Waters Corporation) connected to a Synapt Q-ToF HRMS (Waters Corporation).

The analytes were separated using a BEH $C_{18}$, 1.7 µm, 100 × 2.1 mm column (Waters Corporation). 0.1% formic acid in MilliQ water with pH 2.8 was used as mobile phase A, and mobile phase B was MeCN with 0.1% formic acid. The gradient program

started with 10% B and was increased to 99% B over 16 minutes, where it was held for 5 minutes before decreasing back to 10% B over 0.1 minutes. The column was equilibrated for 3 minutes between each injection. The flow rate was 0.3 mL/min, injection volume was 2 µL, sampler temperature was 8 °C and column oven temperature was 35 °C.

All samples were analyzed in positive ESI mode using a scan range of 50.0000 – 1200.0000 *m/z*, the resolution was 26 000 at 268.1912 *m/z*, and accumulation time was 0.35 s. The capillary was set at 0.5 kV, reference capillary was set at 1 kV, the cone voltage was 20 V, source temperature was 120 °C, and desolvation temperature was 500 °C. Gas parameters were cone gas flow: 100 L/h, desolvation gas flow: 500 L/h and nebulizer gas pressure: 5.0 bar. The samples were analyzed in data independent acquisition mode (DIA), applying ramp trap collision from 10 to 40 V.

*Processing details.* The data was processed using MassLynx (version 4.1, Waters Corporation). The peaks were detected and integrated based on exact mass (mass window ± 0.002 Da) and the same compound needed to be detected in a retention time window of ± 0.1 min. If there were several potential candidates, the $MS^2$ fragments were considered to identify the correct peak.

## DS_Q

*Instrumental settings.* The samples were analyzed on a Dionex UltiMate™ 3000 UHPLC system (Thermo Fisher Scientific™) connected to a Q Exactive Orbitrap HRMS (Thermo Fisher Scientific™).

The analytes were separated using a Hypersil GOLD aQ $C_{18}$, 1.9 µm, 100 × 2.1 mm column (Thermo Fisher Scientific™). 0.1% formic acid in MilliQ water with pH 2.7 was used as mobile phase A, and mobile phase B was MeCN with 0.1% formic acid. The gradient program started with 5% B and increased to 99% B over 9 minutes, where it was held for 4 minutes before decreasing back to 5% B over 0.1 minutes. The column was equilibrated for 2 minutes between each injection. The flow rate was 0.4 mL/min, injection volume was 5 µL, sampler temperature was 10 °C and column oven temperature was 40 °C.

All samples were analyzed in positive ESI mode with the following settings: number of microscans were 1, resolution was set to 120 000, AGC target was $3\times10^6$, maximum IT was 120 ms, number of scans were 2 (73.0000 – 800.0000 *m/z* and 150.0000 – 1500.0000 *m/z*). Sheath gas flow rate was 30 AU, aux gas flow rate was 5 AU, sweep gas flow rate was 0 AU, spray voltage was 3.70 kV, spray current was 8 µA, capillary temperature was 320 °C, S-lens RF level was 55.0%, and aux gas heater temperature was 320 °C.

*Processing details.* The data was processed using TraceFinder version 4.1 (Thermo Scientific™). The peaks were detected and integrated based on exact mass of the $[M+H]^+$ ions (or sometimes the ammonium adduct or in-source fragments, this was given in the instructions) with a mass deviation of 10 ppm.

## DS_QBD

*Instrumental settings.* The samples were analyzed using a Nexera Prominence HPLC (Shimadzu Corporation) connected to an X500R Q-ToF HRMS (Sciex Pte. Ltd.).

The analytes were separated using a ZORBAX Eclipse Plus $C_{18}$ column, 150 × 2.1 mm column (Agilent Technologies Inc.). 0.1% formic acid in water with pH 2.6 was used as mobile phase A, and mobile phase B was MeCN with 0.1% formic acid. The gradient program started with 2% B for 1 minute, was increased to 20% B over 1 minute, increased to 100% B over 14.5 minutes, where it was held for 10.5 minutes before decreasing back to 2% B over 0.1 minutes. The column was equilibrated for 10 minutes between each injection. The flow rate was 0.3 mL/min, injection volume was 95 µL, sampler temperature was 10 °C and column oven temperature was 40 °C.

All samples were analyzed in positive ESI mode with the $MS^1$ scan range of 100.0000 – 1200.0000 *m/z* and resolution set to 35 000 at 350 *m/z*. Accumulation time was 0.25 s, declustering potential was 60 V, collision energy was 10 V, spray voltage was 5.50 kV, and capillary temperature was 550 °C. Ion source gas pressure 1 was 35 psi, ion source gas pressure 2 was 45 psi, curtain gas pressure was 40 psi, and CAD gas was 7 AU.

For $MS^2$ both data dependent and data independent acquisition was used. Samples were analyzed in triplicates with the same $MS^1$ settings, while for $MS^2$, two injections were analyzed with data dependent and one with data independent mode. For data dependent $MS^2$, up to 12 precursor ions per cycle were selected for fragmentation. In data independent mode, 16 SWATH windows with flexible mass ranges were used. The accumulation time for each $MS^2$ experiment was 65 ms and 50 ms, respectively,

leading to a total cycle time of roughly 1.1 sec. The $MS^2$ mass range was 30.0000 – 1200.0000 *m/z*. A collision energy of 35 V and a collision energy spread of 20 V was used. Declustering potential and ion source parameters were taken from $MS^1$.

*Processing details.* The data was processed using Sciex OS software (version 2.1, Sciex Pte. Ltd.). Based on the elemental composition of the compounds, extracted ion chromatograms with a width of +/- 20 ppm were extracted for each compound. For known compounds, the retention time window was restricted to 0.2 min around the expected one. For compounds which were not available in the lab, the $MS^2$ data were compared with open spectral libraries to make sure the correct retention time was assigned. Afterwards, the XICs were manually integrated. For each compound, the accurate mass (< 10 ppm) and the isotope pattern match were verified. Finally, the peaks heights were exported for all compounds and samples.

## DS_QDF

*Instrumental settings.* The samples were analyzed on a Dionex UltiMate™ 3000 ultra-high performance (UHP)LC system (Thermo Fisher Scientific™) connected to a Q Exactive Orbitrap HRMS (Thermo Fisher Scientific™).

The analytes were separated using a Kinetex 2.6 µm, EVO $C_{18}$, 100 Å, 150 × 3.0 mm column (Phenomenex®). 0.1% formic acid in HPLC-grade water with pH 2.7 was used as mobile phase A, and mobile phase B was MeCN. The gradient program started with 5% B and increased to 100% B over 20 minutes, where it was held for 5 minutes before decreasing back to 5% B over 0.1 minutes. The column was equilibrated for 4.9 minutes between each injection. The flow rate was 0.35 mL/min, injection volume was 10 µL, sampler temperature was 4 °C and column oven temperature was 40 °C.

All samples were analyzed in positive ESI mode with the following settings: number of microscans were 1, resolution was set to 120 000, AGC target was $3\times10^6$, maximum IT was 200 ms, and number of scans were 2 (60.0000 – 900.0000 *m/z* and 100.0000 – 1500.0000 *m/z*). Sheath gas flow rate was 35 AU, aux gas flow rate was 3 AU, sweep gas flow rate was 0 AU, spray voltage was 3.50 kV, max spray current was 100 µA, capillary temperature was 320 °C, S-lens RF level was 50.0%, and aux gas heater temperature was 320 °C.

*Processing details.* The data was processed using Quan Browser application for Xcalibur™ software (Thermo Fisher Scientific™). Peaks were detected and integrated based on the *m/z* for the ion/ions for each compound, e.g., $[M+H]^+$, $[M+Na]^+$, $[M+NH_4]^+$, $[M+2H]^{2+}$, using a mass tolerance of 10 ppm. Peak areas and RTs were exported to Excel (Microsoft Corp., USA).

## DS_QDJ

*Instrumental settings.* The samples were analyzed using an Acquity UPLC system (Waters Corporation) connected to a Xevo G2-SQToF HRMS (Waters Corporation).

The analytes were separated using a BEH $C_{18}$, 1.7 µm, 150 × 2.1 mm column (Waters Corporation). 0.05% formic acid in water with pH 2.9 was used as mobile phase A, and mobile phase B was MeCN with 0.05% formic acid. The gradient program started with 2% B for 0.5 minute and was increased to 99% B over 18 minutes, where it was held for 4 minutes before decreasing back to 2% B over 2.5 minutes. The flow rate was 0.45 mL/min, injection volume was 5 µL, sampler temperature was 10 °C and column oven temperature was 35 °C.

All samples were analyzed in positive ESI mode using a scan range of 50.0000 – 1200.0000 *m/z*, the resolution was 31 000 at 556.2766 *m/z*, and accumulation time was 0.25 s. The capillary was set at 0.5 kV, reference capillary was set at 3 kV, the cone voltage was 30 V source temperature was 120 °C, and desolvation temperature was 600 °C. Gas parameters were cone gas flow: 50 L/h, desolvation gas flow: 900 L/h. $MS^1$ collision energy (CE) was 6 eV, $MS^e$ collision energy (CE) was a ramp between 6 eV to 45 eV.

For $MS^e$ experiments, two acquisition functions with different collision energies were created: the low energy function (LE), selecting a collision energy of 6 eV, and the high energy (HE) function, with a collision energy ramp ranging from 6 eV to 45 eV in order to promote fragmentation. This approach enables the simultaneous acquisition of both parent (deprotonated or protonated molecules) and fragment ions in a single injection.

*Processing details.* The data was processed using UNIFI™ Scientific Information System software (version 1.9.4, Waters Corporation). The peaks were detected and integrated based on homemade library created in UNIFI software. Each suspect entry in the library contained the name of the molecule, its exact mass, its retention time, its raw formula, experimental fragments and its molecular structure, allowing in silico fragmentation. Suspects were targeted by their exact mass with a tolerance of 10

ppm, the detected fragments were compared to the predicted or experimented ones using a tolerance window of 2 mDa and their retention time with a tolerance window of 0.2 min.

## DS_QJS

*Instrumental settings.* Samples were analyzed on a Dionex UltiMate™ 3000 UHPLC system (Thermo Fisher Scientific™) connected to a Maxis HD Q-ToF mass spectrometer (Bruker Daltonics).

The analytes were separated using a BEH $C_{18}$, 1.7 µm, 50 × 2.1 mm column (Waters Corporation). Mobile phase A was water with 1 mM ammonium fluoride at pH 5.6, and mobile phase B was MeOH. The gradient program started with 5% B for 3 minutes, and increased to 60% B in 1 minute, where it was held for 10 minutes. The gradient then increased to 98% B in 0.1 min, where it was held for 3 minutes before decreasing back to 5% B over 0.1 minutes. The column was equilibrated for 6 minutes at 5% B between each injection. The flow rate was 0.4 mL/min, injection volume was 90 µL, sampler temperature was 5 °C and column oven temperature was 40 °C.

All samples were analyzed in positive ESI mode with the following settings: spectra rate was 3.33 spectra/s, resolution was set to 45 000 (at 200 *m/z*), and the scan range was 80.0000 – 1000.0000 *m/z*. The capillary voltage was 4.5 kV, spray current was 2.506 µA, capillary temperature was 260 °C, dry gas flow was 11 L/min, nebulizer pressure was 3 bar, end plate offset was -500 V, dry temperature was 220 °C, funnel RF was 300 Vpp, and multipole RF was 300 Vpp. For $MS^2$, the bbCID was used with the following settings for ESI(+): 0.0 eV as CID energy for MS and MS/MS, while collision energies were 5.0 and 20.0 eV respectively.

Instrument mass calibration was conducted injecting 10 µL of a calibrant solution (3 parts of 1 M NaOH to 97 parts of 50:50 water:IPA with 2% FA) at the beginning of each run, before the sample injection. The resulting peak was taken as reference for internal mass calibration via DataAnalysis software (Compass DataAnalysis v. 4.3, Bruker).

*Processing details.* The data was processed using DataAnalysis software (Compass DataAnalysis v. 4.3, Bruker). In-house R scripts were loaded into the DataAnalysis environment for peaks identification and integration. For a given list of analytes of interest and their MS/MS spectra, each peak (identified by its *m/z* and retention time) was assigned to a specific analyte based on following criteria: (a) *m/z* tolerance (<5 ppm), (b) retention time, and (c) MS/MS spectra from bbCID.

## DS_QQG

*Instrumental settings.* The samples were analyzed on an Acquity UHPLC system (Waters Corporation) connected to a Q Exactive Orbitrap HRMS (Thermo Fisher Scientific™).

The analytes were separated using a CORTECS $C_{18}$, 2.7 µm, 100 × 2.1 mm column (Waters Corporation). Mobile phase A was 0.1% formic acid in water with pH 2.7, and mobile phase B was MeOH with 0.1% formic acid. The gradient program started with 5% B and increased to 75% B over 7 minutes, then increased to 100% B over 3 minutes where it was held for 5 minutes before decreasing to 5% B over 2 minutes. The column was equilibrated for 4 minutes between each injection. The flow rate was 0.3 mL/min, injection volume was 10 µL, sampler temperature was 10 °C and column oven temperature was 40 °C.

All samples were analyzed in positive ESI mode with the following settings: number of microscans were 3 160, resolution was 70 000, AGC target was $3\times10^6$, maximum IT was 150 ms, and the scan range was 66.7000 – 1000.0000 *m/z*. Sheath gas flow rate was 40 AU, aux gas flow rate was 10 AU, sweep gas flow rate was 2 AU, spray voltage was 4.0 kV, spray current was 15 µA, capillary temperature was 350 °C, S-lens RF level was 60.0%, and the aux gas heater temperature was 250 °C. Data was acquired in DIA mode, with a nominal collision energy of 35eV HCD.

*Processing details.* The data was processed using Xcalibur software (version 2.2, Thermo Fisher Scientific™). The peaks were detected and integrated based on their accurate mass (1 mDa error) and manual inspection. When available, compounds' HCD spectra was searched in mzCloud online database and the most intense fragment ion was searched in the HE function to confirm compounds' identity.

## DS_QQT

*Instrumental settings.* The samples were analyzed using an Acquity I UPLC system (Waters Corporation) connected to a Xevo G2-SQToF HRMS (Waters Corporation).

The analytes were separated using an Acquity BEH $C_{18}$, 1.7 µm, 100 × 2.1 mm column (Waters Corporation). Mobile phase A was water with 10 mM ammonium acetate with pH 6.75, and mobile phase B was MeOH with 10 mM ammonium acetate. The gradient program started with 2% B for 0.25 minutes and was increased to 99% B over 12 minutes, where it was held for 0.75 minutes before decreasing back to 2% B over 0.01 minutes. The column was equilibrated for 4 minutes between each injection. The flow rate was 0.45 mL/min, injection volume was 50 µL, sampler temperature was 10 °C and column oven temperature was 45 °C.

All samples were analyzed in positive ESI mode using a scan range of 50.0000 – 1200.0000 *m/z*, the resolution was 20 000 at 785.8 *m/z* and 10 000 at 175.1 *m/z*, and scan time was 0.250s. The capillary was set at 1.0 kV, reference capillary was set at 3 kV, the cone voltage was 20 V, source temperature was 120 °C, and desolvation temperature was 550 °C. Gas parameters were cone gas flow: 20 L/h, desolvation gas flow: 1000 L/h.

The $MS^e$ experiment was used with two acquisition functions; the low – energy function with a collision energy of 4eV, and the high -energy function with a collision energy ramp ranging from 10 to 40 eV.

*Processing details.* The data was processed using UNIFI™ Scientific Information System software (1.9.4.053, Waters Corporation). The known compounds were added to the screening database by mol file. First identification was done via 3D peak detection and structural data from mol file or data provided from the trial. From confirmed RT and expected *m/z*, a compound list was created for the quantitative processing. Processing the data for quantitative analysis was done with automatic 2D peak detection with a mass tolerance of 2.0 mDa.

## DS_QSB

*Instrumental settings.* The samples were analyzed using an Dionex UltiMate 3000 RSLC UHPLC (Thermo Fisher Scientific™) connected to a Maxis Impact QToF HRMS (Bruker Daltonics).

The analytes were separated using an Acclaim RSLC $C_{18}$, 2.2 µm, 100 × 2.1 mm column (Thermo Fisher Scientific™) preceded by an Acquity UPLC BEH $C_{18}$, 1.7 µm VanGuard pre-column (Waters Corporation, Ireland). Mobile phase A was H2O:MeOH 90:10 mixture with 5 mM ammonium formate and 0.01% formic acid, and mobile phase B was MeOH with 5 mM ammonium formate and 0.01% formic acid. The gradient program started with 1% B for 1 minute and was increased to 39% B over 2 minutes, then to 99.9% B over 11 minutes, where it was held for additionally 2 minutes before decreasing back to 1% B in 0.1 minutes. Then, the initial mobile phase composition of 1% B was held for 3.9 minutes between each injection. The flow rate varied together with the gradient, starting at 0.2 mL/min for 3 minutes, then increased to 0.4 mL/min over 11 minutes and finally increased to 0.48 mL/min over 2 minutes. The column was first equilibrated at 0.48 mL/min and 1% B for 3 minutes, then at a flow rate of 0.2 mL/min and 1% B for 1 minute between each injection. The injection volume was 5 µL, sampler temperature was 20-25 °C and column oven temperature was 30 °C.

All samples were analyzed in positive ESI mode using a scan range of *m/z* 50.0000 – 1000.0000, the resolution varied between 36 000-40 000 (39 274 at *m/z* 226.1593, 36 923 at *m/z* 430.9137, and 36 274 at *m/z* 702.8636), and the scan time was 2 Hz/cycle. The capillary voltage was 2.5 kV, end plate offset was 500 V, nebulizer pressure was 2 bar, drying gas flow rate was 8 L/min, and the dry temperature was 200 °C.

The QToF MS system operated in broadband collision-induced dissociation (bbCID) DIA mode, where two sequential full scan events were triggered; the first scan at low collision energy (4 eV) resulting in an $MS^1$ full scan and the second scan at high collision energy (25 eV) resulting in an $MS^2$ all ion fragment mode. MS data was acquired in $HRMS^e$ mode with ion mobility and $MS^e$ fragmentation. The HRMS system was calibrated externally daily, and internally at the beginning of each run using a $H_2O$:isopropanol (1:1) mixture with 10 mM sodium formate.

*Processing details.* Post-acquisition data treatment was implemented with Data Analysis 4.4 and TASQ 1.4 software (Bruker Daltonics, Bremen, Germany).

## DS_QV

*Instrumental settings.* The samples were analyzed on a Dionex UltiMate™ 3000 DGP 3600RS system (Thermo Fisher Scientific™) connected to an Orbitrap Exploris 240 HRMS (Thermo Fisher Scientific™).

The analytes were separated using an Atlantis T3 $C_{18}$, 3 µm, 150 × 3 mm column (Waters Corporation). 0.1% formic acid in water with pH 2.5 was used as mobile phase A, mobile phase B was MeOH with 0.1% formic acid, and mobile phase C was isopropanol. The gradient program started with 0% B for 1.5 minutes and increased to 95% B over 17 minutes, where it was held for 4.5 minutes. The mobile phase composition changed to 90% B and 5% C over 0.5 minutes, which was held for 5 minutes before decreasing back to 0% B and 0% C over 3.5 minutes. The column was equilibrated for 12 minutes between each injection. The flow rate was 0.3 mL/min, injection volume was 100 µL, sampler temperature was 5 °C and column oven temperature was 30 °C.

All samples were analyzed in positive ESI mode with the following settings: number of microscans were 1 and 2 scan ranges were used: 65.0000 – 110.0000 *m/z* and 100.0000 – 1000.0000 *m/z*. The resolution was set to 60 000 and 120 000 and maximum IT was set to auto and 100 ms, for the lower and higher scan range respectively. Sheath gas flow rate was 40 AU, aux gas flow rate was 10 AU, sweep gas flow rate was 0 AU, spray voltage was 3.50 kV, capillary temperature was 320 °C, S-lens RF level was 70.0%, and the aux gas heater temperature was turned off.

$MS^2$ was obtained at a resolution of 15 000 with an isolation window of 1 *m/z* and normalized HCD collision energy of 30%. Additional settings: Multiplex ions: false; Isolation Offset: off; Scan Range Mode: auto; AGS Target Standard; Maximum Injection Time: auto; Microscans: 1; Data Type: centroid.

*Processing details.* The data was processed using TraceFinder software (version 5.1, Thermo Fisher Scientific™). The peaks were detected automatically and afterwards inspected manually. Following the initial data processing the resulting lists of calibration compounds and standard compounds were processed as the following steps: (1) Calibration compounds that were not detected or deemed unsatisfactory were removed from the list, e.g., chlormequat (no peak) and histamine (bad peak shape). (2) For early eluting compounds (RT < 3 min), the first replicate (sample name ends with R1) was measured with an active divert valve, i.e., the sample was discarded for the first three minutes and thus this replicate did not produce meaningful values. (3) The calibration curves were examined manually and some points were removed (cells in the data sheet were left blank) based on: (a) lack of linearity (the highest concentration); (b) lack of reproducibility (low intensity and CV > 35 %); (c) decreasing the quality of calibration curve (R < 0.95). In each case, peaks were reintegrated manually in Xcalibur as a check. (4) Nothing was removed from the 'samples', despite e.g., high CV values between the replicates and low signals. (5) In the blanks the signals were corrected as in the calibration solutions. (6) Dichlorvos measurements resulted in a poor calibration curve (R = 0.96 with IS, 0.92 without IS). It was not removed because it was used as a standard compound in the MLR-*IE* approach. However, the low quality of the calibration curve probably affected the concentrations obtained with this method. The resulting tables were used as input to different concentration estimation methods.

## DS_STF

*Instrumental settings.* Samples were analyzed on a Vanquish LC system (Thermo Fisher Scientific™) connected to an Exploris 480 Orbitrap HRMS (Thermo Fisher Scientific™).

The analytes were separated on a Kinetex Biphenyl, 2.7 µm, 100 × 3.0 mm column (Phenomenex®). Mobile phase A was 0.02% formic acid and 0.2 mM ammonium formate in water with pH 3 and mobile phase B was MeOH. The gradient program started with 20% B and was increased to 100% B over 25 minutes, was held at 100% B for 12 minutes, before decreasing to 0% B over 0.01 minutes. The column was equilibrated at 0% B for 8 minutes between each injection. The flow rate was 0.15 mL/min, injection volume was 100 µL and sampler temperature was 5 °C.

All samples were analyzed in positive ESI mode with these settings: number of microscans were 1, resolution was set to 120 000, AGC target was $3\times10^6$, maximum IT was 100 ms, and the scan range was 100.0000 – 1000.0000 *m/z*. Sheath gas flow rate 30 AU, aux gas flow rate 6 AU, sweep gas flow rate 0 AU, spray voltage 3.1 kV, capillary temperature 250 °C, the S-lens RF level 40.0%, and aux gas heater temperature 300 °C.

For $MS^2$, a resolution of 15 000 was used. HCD energies were 15, 30, and 50 V.

*Processing details.* The data was processed using Xcalibur v. 4.1(Thermo Fisher Scientific™). The peaks were detected and integrated based on the exact mass of the components.

## DS_TBJ

*Instrumental settings.* The samples were analyzed using an Acquity UPLC I-Class system (Waters Corporation) connected to a Vion IMS Q-ToF HRMS (Waters Corporation).

The analytes were separated using an Acquity UPLC BEH $C_{18}$, 1.7 µm, 100 × 2.1 mm column (Waters Corporation). 0.1% formic acid in water with pH 3 was used as mobile phase A, and mobile phase B was MeCN with 0.1% formic acid. The gradient program started with 2% B for 1 minute and was increased to 98% B over 24 minutes, where it was held for 5 minutes before decreasing back to 2% B over 1.5 minutes. The column was equilibrated for 2.5 minutes between each injection. The flow rate was 0.45 mL/min, injection volume was 10 µL, sampler temperature was 10 °C and column oven temperature was 40 °C.

All samples were analyzed in positive ESI mode using a scan range of 50.0000 – 1000.0000 *m/z*, the resolution was 30 000 at 516 *m/z*, and accumulation time was 0.2 s. The capillary was set at 0.8 kV, reference capillary was set at 3 kV, the cone voltage was 40 V, source offset was 80 V, source temperature was 120 °C, and desolvation temperature was 250 °C. Gas parameters were cone gas flow: 50 L/h, desolvation gas flow: 1000 L/h. StepWave RF was 250 V and Trap/IMS RF was 250 V.

High Definition $MS^E$ was used for data-independent $MS^2$ acquisition, with 50.0000 – 1000.0000 m/z range, 0.2 s scan time, collision energy at low energy: 6 V and collision energy ramp at high energy: 20-56 V.

*Processing details.* The data was processed using UNIFI™ Scientific Information System software (version 1.9.4.053, Waters Corporation). The peaks were detected and integrated based on the following 4D detection parameters: RT range 0-30 min, mass range 50-1000 m/z, with intensity thresholds in high and low energy of 50 counts and 80 counts respectively, background filter was set on high, and the maximum number of peaks to keep per channel set to 300 000. Lock mass correction was applied.

## DS_TDF

*Instrumental settings.* Samples were analyzed on a Vanquish UPLC (Thermo Fisher Scientific™) connected to a Fusion Lumos Orbitrap (Thermo Fisher Scientific™).

Analytes were separated on an Acquity HSS T3 $C_{18}$, 1.8 µm, 150 × 2.1 mm column (Waters Corporation). 0.1% formic acid in water with pH 2.7 was used as mobile phase A and mobile phase B was MeOH with 0.1% formic acid. The gradient program started with 2% B for 1 minute and was increased to 98% B over 15 minutes, where it was held for 5 minutes, before decreasing back to 2% B over 1 minute. The column was equilibrated for 6 minutes between each injection. The flow rate was 0.3 mL/min, injection volume was 10 µL, sampler temperature was 10 °C and column oven temperature was 35 °C.

All samples were analyzed in positive ESI mode with the following settings: number of microscans were 1, resolution was set to 120 000, AGC target was $5\times10^5$, maximum IT was 50 ms, and the scan range was 70.0000 – 1450.0000 *m/z*. Sheath gas flow rate was 50 AU, aux gas flow rate was 10 AU, sweep gas flow rate was 1 AU, spray voltage was 3.50 kV, capillary temperature was 350 °C, ion transfer tube temperature 325 °C, and the S-lens RF level was 60.0%.

*Processing details.* The data was processed using Skyline software (21.2.0.369, University of Washington). The peaks were detected and integrated based on retention time (± 0.5 min), mass tolerance (0.0001 *m/z*), and MS1 filtering of 120 000 @ 200 *m/z*.

## DS_TJQ

*Instrumental settings.* The samples were analyzed on an LC30AD system (Shimadzu Corporation) connected to a Maxis HRMS (Bruker Daltonics).

The analytes were separated using a Kinetex EVO $C_{18}$, 2.6 µm, 100 × 2.1 mm column (Phenomenex®). Mobile phase A was water with 0.1% formic acid at pH 2.7, and mobile phase B was MeCN. The gradient program started with 5% B and increased to 100% B in 15 minutes, where it was held for 5 minutes before decreasing back to 5% B in 0.1 minutes. The column was equilibrated for 7 minutes at 5% B between each injection. The flow rate was 0.2 mL/min, injection volume was 20 µL, sampler temperature was 15 °C and column oven temperature was 40 °C.

All samples were analyzed in positive ESI mode with the following settings: spectra rate was 5 Hz and the scan range was 20.000 – 1000.0000 *m/z*. Capillary voltage was 3.5 kV, dry gas flow was 8.0 L/min, nebulizer pressure was 1.0 bar, end plate offset was

500 V, dry temperature was 200 °C, funnel RF was 200 Vpp and multipole RF was 200 Vpp. Pre-Pulse Storage was 8.0 µs, transfer time was set at 45.0 µs. Collision RF was stepped from 175.0 to 350.0 Vpp, with 30/70% timing.

*Processing details.* The data was processed using TASQ software (2021, Bruker). The peaks were detected and integrated based on retention time (<= 0.2 min) and accurate mass (<= 5 ppm).

## DS_TL

*Instrumental settings.* The samples were analyzed using an Agilent Infinity 1290 LC system (Agilent Technologies Inc.) connected to an Agilent 6530 Q-ToF HRMS (Agilent Technologies Inc.). The LC system was equipped with a high performance autosampler (model G4226A), column compartment (model G1316C), an isocratic (model G1310A) and binary (model G4220B) pump for constant infusion of the reference mass solution and gradient elution, respectively.

The analytes were separated using a Poroshell 120 EC-$C_{18}$, 2.7 µm, 150 × 3 mm column (Agilent Technologies Inc.). 0.1% formic acid in water with pH 2.74 was used as mobile phase A, and mobile phase B was MeCN with 0.1% formic acid. The gradient program started with 5% B and was increased to 99% B over 15 minutes, where it was held for 2 minutes before decreasing back to 5% B over 0.1 minutes. The column was equilibrated for 5 minutes between each injection. The flow rate was 0.35 mL/min, injection volume was 10 µL, sampler temperature was 6 °C and column oven temperature was 40 °C.

The samples were analyzed in positive ESI mode with the following settings: scan rate was 4 spectra/s, resolution was 8 500 at 922 *m/z* and scan range was 65.0000 – 1600.0000 *m/z*. The drying gas temperature was 300 °C, drying gas flow rate was 9 L/min, nebulizer was 35 psig, VCap was 3.00 kV, sheath gas temperature was 350 °C, sheath gas flow was 11 L/min, nozzle was 500V, fragmentor was 120 V, skimmer 1 was 65 V and OctopoleRFPeak was 750 V.

*Processing details.* The data was processed using the MassHunter Qualitative Analysis software (version B.07.00; Agilent Technologies Inc.). From the raw data files, extracted ion chromatograms (EIC) of the ion mass of the compound of interest (calibrant or suspect) were extracted using the "extract chromatogram" algorithm of the software (mass window: 20 ppm). The obtained peaks were checked for retention time consistency among the calibration/suspects' samples. The obtained chromatograms were smoothed using a Gaussian smoothing function (function width: 15 points; Gaussian width: 5 points). The smoothed chromatograms were integrated using the Agile 2 integrator incorporated in the software. The obtained peak area was reported.

## DS_TSF

*Instrumental settings.* The samples were analyzed on a Dionex UltiMate™ 3000 UHPLC system (Thermo Fisher Scientific™) connected to a 5600+ Triple ToF HRMS (Sciex Pte. Ltd.).

The analytes were separated using an RRHD Eclipse Plus $C_{18}$, 1.8 µm, 150 × 2.1 mm column (Agilent Technologies Inc.). 0.1% formic acid in water with pH 3 was used as mobile phase A, and mobile phase B was MeCN with 0.1% formic acid. The gradient program started with 2% B for 1 minute and increased first to 20% B in 1 minute and then to 100% B over 14 minutes. The gradient was held at 100% B for 8 minutes before decreasing back to 2% B over 1 minute. The column was equilibrated for 5 minutes between each injection. The flow rate was 0.3 mL/min, injection volume was 50 µL, sampler temperature was 10 °C and column oven temperature was 40 °C.

All samples were analyzed in positive ESI mode with an acquisition method based on double experiments, i.e., full-scan survey ToF-MS and IDA (Information Dependent Acquisition) experiment in a scan range of 50.0000 – 1000.0000 *m/z*, either in ToF-MS (resolution of 30 000 at 829.5393 *m/z*) or ToF-MS/MS experiments.

Accumulation time was 0.200 s for ToF-MS and 0.07 s for IDA scan; declustering potential was 80 V, collision energy was 35 V, collision energy spread was 15 V, spray voltage was 5.5 kV, and capillary temperature was 500 °C. Ion source gas pressure 1 was 35 psi, ion source gas pressure 2 was 45 psi, curtain gas pressure was 20 psi, and CAD gas 6 psi. The maximum number of candidate ions to monitor per cycle during IDA experiments was set to 10, for ions exceeding a peak intensity threshold of 200 cps; the mass tolerance was set to 50 mDa and dynamic background subtraction was activated.

The mass spectrometer was calibrated automatically using standards recommended by AB SCIEX for calibrating the AB SCIEX Triple ToF® 5600 Instrument by means of a Calibrant Delivery System (CDS), every 5 samples inserted into the queue of submitted samples.

*Processing details.* The data was processed using Sciex OS software (version 2.0.1, AB SCIEX™). The peaks were detected and integrated based on mass accuracy error (< 5 ppm), plausible retention time in the chromatogram, isotopic fit (> 90%) and the presence of a fragmentation MS/MS pattern.

In detail, as for the peak integration the following parameters were used: XIC width 0.02 Da, RT half window 30.0 s, minimum peak width 3 points, noise percentage 40%.

## DS_TSJ

*Instrumental settings.* Samples were analyzed on an Agilent 1100 LC system (Agilent Technologies Inc.) connected to an LTQ Orbitrap Discovery HRMS (Thermo Fisher Scientific™).

The analytes were separated on a Hypersil GOLD $C_{18}$, 3.0 µm, 100 × 2.1 mm column (Thermo Fisher Scientific™). Mobile phase A was 0.05% formic acid in water with pH 4 and mobile phase B was 0.05% formic acid in MeOH. The gradient program started with 5% B and was increased to 95% B over 25 minutes, was held at 95% B for 5 minutes, before decreasing back to 5% B over 0.1 minutes. The column was equilibrated at 5% B for 10 minutes between each injection. The flow rate was 0.2 mL/min, injection volume was 20 µL, sampler temperature was 4 °C and column oven temperature was 35 °C.

All samples were analyzed in positive ESI mode with these settings: number of microscans were 3, resolution was set to 30 000, AGC target was $2\times10^5$, maximum IT was 500 ms, and the scan range was 80.0000 – 2000.0000 *m/z*. Sheath gas flow rate 40 AU, aux gas flow rate 15 AU, sweep gas flow rate 0 AU, spray voltage 3.5 kV, spray current 100 µA, capillary temperature 275 °C.

$MS^2$ was performed with data dependent method in order to confirm the identification: threshold 1000 counts, top 3, dynamic exclusion 20 s, Activation type CID, Isolation width 2 m/z, Normalized collision energy 35, Activation Q 0.25, activation time 30 ms.

*Processing details.* The data was processed using TraceFinder EFS (version 3.2, Thermo Fisher Scientific Inc). The peaks were detected and integrated based on HRMS signal. The parameters are the following: (1) Mass tolerance: 5 ppm; (2) Detection: (a) Algorithm: ICIS; (b) Detection method: Nearest RT; (c) Smoothing: 1.

## DS_VQL

*Instrumental settings.* The samples were analyzed on an Acquity UPLC® system consisting of a Binary Solvent Manager, Sample Manager FTN-1 and Column Manager (Waters Corporation) coupled to a Select Series Cyclic IMS Q-ToF HRMS (Waters Corporation).

The analytes were separated using a Kinetex 2.6 µm, EVO $C_{18}$, 100 Å, 150 × 3.0 mm column (Phenomenex®). 0.1% formic acid in HPLC-grade water with pH 2.7 was used as mobile phase A, and mobile phase B was MeCN. The gradient program started with 5% B and increased to 100% B over 20 minutes, where it was held for 5 minutes before decreasing back to 5% B over 0.1 minutes. The column was equilibrated for 4.9 minutes between each injection. The flow rate was 0.35 mL/min, injection volume was 10 µL, sampler temperature was 4 °C and column oven temperature was 40 °C.

All samples were analyzed in positive ESI mode using a scan range of 50.0000 – 1200.0000 *m/z*. The capillary was set at 2.60 kV, reference capillary was set at 1.00 kV, the cone voltage was 40 V, source offset was 10 V, source temperature was 150 °C and desolvation temperature was 550 °C. Gas parameters were cone gas: 0 L/h, desolvation gas: 800 L/h and nebulizer gas: 6.0 bar. Trap collision energy (CE) was 6.0 V, transfer CE was 4.0 V, transfer RF was 150 V. The analyzer was set to V-mode.

*Processing details.* The data was processed using UNIFI™ Scientific Information System software (Waters Corporation). Peaks were detected and integrated based on expected mass for the neutral compound, information regarding adduct type (+H, +Na, $+NH_4$, -e) and expected RT.

## DS_YF

*Instrumental settings.* The samples were analyzed on a Dionex UltiMate™ 3000 UHPLC (Thermo Fisher Scientific™) connected to a Q Exactive Focus Orbitrap HRMS (Thermo Fisher Scientific™).

The analytes were separated using a Raptor AR $C_{18}$, 2.6 µm, 100 × 2.1 mm column (Restek™). Mobile phase A was 0.1% formic acid in water with pH 2.7, and mobile phase B was MeCN with 0.1% formic acid. The gradient program started with 13% B for 0.5 minutes and increased to 50% B over 9.5 minutes, where it was held for 0.75 minutes before increasing to 95% B over 3.5 minutes, and then decreased to 13% B over 0.5 minutes. The column was equilibrated at 13% B for 4 minutes between each injection. The flow rate was 0.25 mL/min, injection volume was 10 µL, sampler temperature was 12 °C and column oven temperature was 30 °C.

All samples were analyzed in positive ESI mode with the following settings: number of microscans were 1, resolution was 70 000, AGC target was $1\times10^6$, maximum IT was set to auto, and the scan range was 70.0000 – 1000.0000 *m/z*. Sheath gas flow rate was 40 AU, aux gas flow rate was 10 AU, sweep gas flow rate was 0 AU, spray voltage was 3.5 kV, spray current was 3.5 µA, capillary temperature was 300 °C, S-lens RF level was 50.0%, and the aux gas heater temperature was 300 °C.

A positive data dependent confirmation was made.

*Processing details.* Data were processed using Xcalibur™ software (v. 2.6, Thermo Fisher Scientific™). The peaks were detected and integrated using the NORMAN processing method provided by the organizers of the trial (no parameters were changed), adjusting the retention time taking into account the internal chromatographic conditions. All peak areas were exported to an Excel file using the Xcalibur™ Quan file function and subsequently processed according to the indications provided in the trial.

## SI 3. REPROCESSING SETTINGS FOR ALL RAW DATA (ANONYMIZED)

The raw data was converted to the *mzML* file format using MSConvert from ProteoWizard,[21] and reprocessed using *patRoon* package v.2.2.0[22] in R v. 4.2.1.[23] The algorithm used for feature extraction was openMS, and suspect screening using a suspect list with *m/z*, SMILES, adduct information and, if applicable, fragments *m/z* for each compound was implemented. Across all datasets, the following parameter settings were used. For finding the features, expected chromatographic FWHM was set to 10 s, and minimum and maximum FWHM was set to 1 s and 30 s, respectively. The features were grouped using retention time alignment, allowing a maximum retention time difference of 40 s. Suspect screening was performed using a *m/z* window of 0.002 and only the matched feature groups were kept. The lists of features were filtered, keeping features with minimum relative replicate abundance: 0.5, maximum standard deviation within a replicate group: 0, blank threshold: 5, retention time range: 50 s – infinity, and *m/z* range: 80 – 1000.

Due to the different instrumentation used in analysis across the datasets, some parameter settings varied across the datasets, namely noise intensity threshold and signal-to-noise ratio (S/N) for finding the features, and minimum absolute intensity in the filtering stage. These parameters were tuned for each dataset using the settings resulting in the maximum number matches to the calibration compounds for each dataset and can be found below. The peak qualities of remaining features were calculated, and those with Gaussian similarity score > 30% were kept. Finally, all peaks were manually inspected with regards to their peak quality and retention time alignment between samples/replicas.

**DS_AF.** The raw data was directly recorded using UNIFI™ Scientific Information System software and was not possible to convert to mzML file format, thus this dataset could not be reprocessed and was not included in the reprocessed results.

**DS_AW.** Noise intensity threshold: 100, S/N: 10, minimum absolute intensity: 250, blank threshold: 5.

**DS_AWW.** Noise intensity threshold: 100, S/N: 5, minimum absolute intensity: 250, blank threshold: 5.

**DS_BIJ.** Noise intensity threshold: 1000, S/N: 5, minimum absolute intensity: 500, blank threshold: 5.

**DS_BQW.** Noise intensity threshold: 300, S/N: 5, minimum absolute intensity: 250, blank threshold: 5.

**DS_DBS.** Noise intensity threshold: 1000, S/N: 5, minimum absolute intensity: 500, blank threshold: 5.

**DS_DID.** Noise intensity threshold: 100, S/N: 5, minimum absolute intensity: 250, blank threshold: 5.

**DS_DP.** Noise intensity threshold: 1000, S/N: 5, minimum absolute intensity: 500, blank threshold: 5.

**DS_DX.** Noise intensity threshold: 1000, S/N: 5, minimum absolute intensity: 500, blank threshold: 5.

**DS_EF.** Noise intensity threshold: 1000, S/N: 5, minimum absolute intensity: 500, blank threshold: 5.

**DS_GJT.** Noise intensity threshold: 100, S/N: 10, minimum absolute intensity: 250, blank threshold: 5.

**DS_GS.** Noise intensity threshold: 1000, S/N: 5, minimum absolute intensity: 500, blank threshold: 5.

**DS_GSB.** Noise intensity threshold: 500, S/N: 5, minimum absolute intensity: 250, blank threshold: 5.

**DS_GSW.** No raw data was provided for this dataset; thus, this dataset was not included in the reprocessed results.

**DS_HT.** Noise intensity threshold: 1000, S/N: 5, minimum absolute intensity: 500, blank threshold: 5.

**DS_ITG.** Noise intensity threshold: 500, S/N: 5, minimum absolute intensity: 250, blank threshold: 5.

**DS_JBB.** Noise intensity threshold: 100, S/N: 5, minimum absolute intensity: 250, blank threshold: 5.

**DS_JBQ.** Noise intensity threshold: 300, S/N: 5, minimum absolute intensity: 250, blank threshold: 5.

**DS_JDF.** Noise intensity threshold: 1000, S/N: 5, minimum absolute intensity: 500, blank threshold: 5.

**DS_JL.** Noise intensity threshold: 1000, S/N: 5, minimum absolute intensity: 500, blank threshold: 5.

**DS_JSG.** Noise intensity threshold: 100, S/N: 5, minimum absolute intensity: 250, blank threshold: 5.

**DS_JWW.** Noise intensity threshold: 1000, S/N: 5, minimum absolute intensity: 500, blank threshold: 5.

**DS_MT.** Due to lower mass accuracy for this dataset, using the narrow *m/z* window of 0.002 resulted in zero detections in the suspect screening, thus the *m/z* window was increased to 0.01. The other varying settings were noise intensity threshold: 100, S/N: 5, minimum absolute intensity: 100, blank threshold: 5.

**DS_Q.** Noise intensity threshold: 1000, S/N: 5, minimum absolute intensity: 500, blank threshold: 5.

**DS_QBD.** Noise intensity threshold: 100, S/N: 5, minimum absolute intensity: 250, blank threshold: 5.

**DS_QDF.** Noise intensity threshold: 500, S/N: 5, minimum absolute intensity: 250, blank threshold: 5.

**DS_QDJ.** Noise intensity threshold: 500, S/N: 10, minimum absolute intensity: 250, blank threshold: 5.

**DS_QJS.** Due to lower mass accuracy for this dataset, using the narrow *m/z* window of 0.002 resulted in zero detections in the suspect screening, thus the *m/z* window was increased to 0.01. The other varying settings were noise intensity threshold: 100, S/N: 5, minimum absolute intensity: 100, blank threshold: 5.

**DS_QQG.** Noise intensity threshold: 1000, S/N: 5, minimum absolute intensity: 500, blank threshold: 5.

**DS_QQT.** Noise intensity threshold: 100, S/N: 5, minimum absolute intensity: 250, blank threshold: 5.

**DS_QSB.** Noise intensity threshold: 100, S/N: 5, minimum absolute intensity: 250, blank threshold: 5.

**DS_QV.** Noise intensity threshold: 1000, S/N: 5, minimum absolute intensity: 500, blank threshold: 5.

**DS_STF.** Noise intensity threshold: 1000, S/N: 5, minimum absolute intensity: 500, blank threshold: 5.

**DS_TBJ.** The raw data was directly recorded using UNIFI™ Scientific Information System software and was not possible to convert to *mzML* file format, thus this dataset could not be reprocessed and was not included in the reprocessed results.

**DS_TDF.** Noise intensity threshold: 500, S/N: 5, minimum absolute intensity: 250, blank threshold: 5.

**DS_TJQ.** Noise intensity threshold: 100, S/N: 5, minimum absolute intensity: 250, blank threshold: 5.

**DS_TL.** Noise intensity threshold: 100, S/N: 5, minimum absolute intensity: 250, blank threshold: 5.

**DS_TSF.** Noise intensity threshold: 100, S/N: 5, minimum absolute intensity: 250, blank threshold: 5.

**DS_TSJ.** Noise intensity threshold: 1000, S/N: 5, minimum absolute intensity: 500, blank threshold: 5.

**DS_VQL.** Noise intensity threshold: 100, S/N: 5, minimum absolute intensity: 250, blank threshold: 5.

**DS_YF.** Noise intensity threshold: 1000, S/N: 5, minimum absolute intensity: 500, blank threshold: 5.

## SI 4. STABILITY

The stability of the compounds was evaluated over 14 weeks, i.e., the time from when samples were prepared until the submission deadline for participating labs. Samples were sent to participants frozen; however, due to long shipping distances and small sample volumes, approximately half of the participants received the samples at ambient temperature. Therefore, stability was investigated by analyzing samples stored under different conditions as stated in materials and methods section "Stability tests". Participants were asked to store the samples at -20 °C until analysis, thus consequently, three compounds (i.e., ampicillin, dazomet, and simvastatin) showing signs of degradation when stored at -20 °C were removed from all datasets. One participant reported that samples were stored in a fridge until measurement, therefore 12 additional compounds (i.e., aspartame, avermectin ($NH_4$ adduct), butylamine, clotrimazole, emamectin, histamine, ivermectin, ketoconazole, nigericin, reserpine, rifaximin, spinosad A, and trichlorfon) showing signs of degradation during storage at 4 °C over the three months were removed from this specific dataset. The stability of compounds stored under different conditions is visualized in Figs. S2 – S5, and the raw data and unstable compounds are given in Table S6.

## SI 5. INSOURCE DEGRADATION OF ATRAZINE TPS

During the reprocessing of the raw data, it was observed that the transformation products of atrazine were problematic: in the majority of datasets, multiple chromatographic peaks were observed for each exact mass of atrazine TPs. Based on this, further degradation or fragmentation of these TPs in the ion source were suspected. Thus, based on the calculated log*P* values of atrazine and its TPs, the retention order in reversed phase LC (which was the predominantly used chromatography) was determined to assign the correct peak to the correct TP. It was assumed that the retention order for these compounds would not differ significantly although different columns, mobile phase compositions, pH, etc., were used in the analyses. This assumption was based on the similar functional groups of the investigated TPs and can be compared to the retention order stability for homologue series. For the dataset using hyphenated reversed phase – HILIC chromatography, only one chromatographic peak was observed for each atrazine TP, therefore, no similar analysis was required for this dataset. It is unknown to what extent this analysis was done in the reported results, and whether it was even picked up by the software used by the participants, but it may provide another reason to the lower prediction errors seen for the reprocessed results.

## SI 6. PEAK QUALITY IN REPROCESSED RESULTS

In the reprocessed results, the top five compounds with the highest fold errors (2-aminobenzothiazole, atrazine-2-hydroxy, atrazine-desisopropyl, benzotriazole-5-carboxylic acid and methidathion) were all from the close eluting approach, and the first four were from the same dataset. For the concerned dataset and approach, the four compounds were all quantified based on the same calibrant, namely saccharin, and no other suspect compounds from this dataset had saccharin as closest eluting calibrant. Reevaluation of the integration and calibration curve for saccharin revealed questionable peak quality for saccharin as well as poor linearity.

As for methidathion in the second dataset, it was quantified using the calibration curve of atrazine. Similarly, it was checked in the concerned dataset in which other suspects were quantified using atrazine's calibration curve in the close eluting approach; however, none of these compounds had particularly high prediction errors. However, the peak area integration for methidathion revealed poor peak shape. This indicates that these compounds maybe should have been removed from the dataset. On the other hand, the reprocessing procedure and consecutive quality assessment of the peaks were, while thorough, deliberately quite generous, in order to keep as many compounds as possible. Therefore, calibration compounds with imperfect curves and/or peaks were kept, as were suspect compounds with imperfect peak shapes.

These examples highlight the instability of the quantification approaches relying on a single surrogate standard.

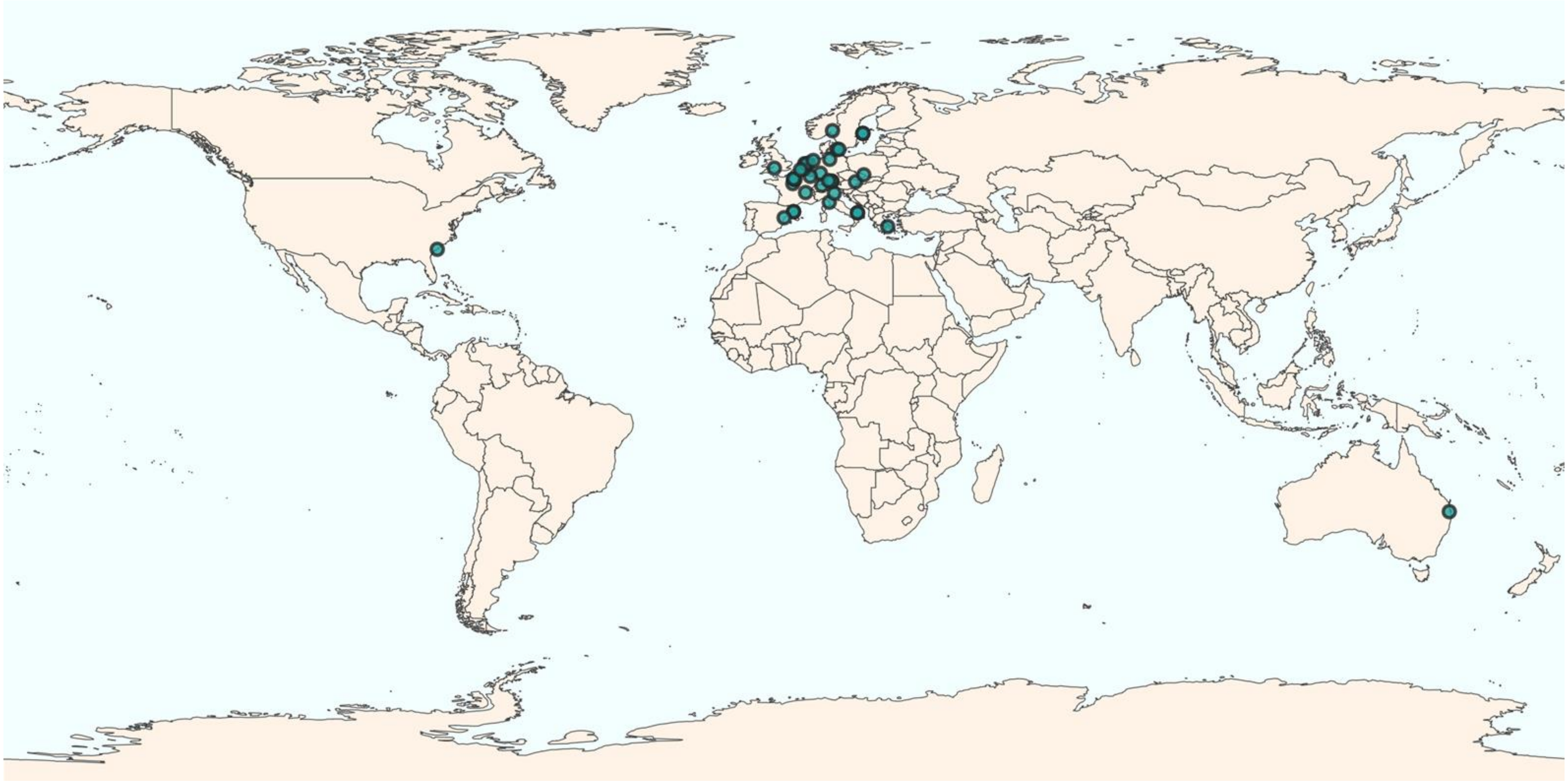

**Fig. S1.** Map showing the geographical position of all participating laboratories in the study.

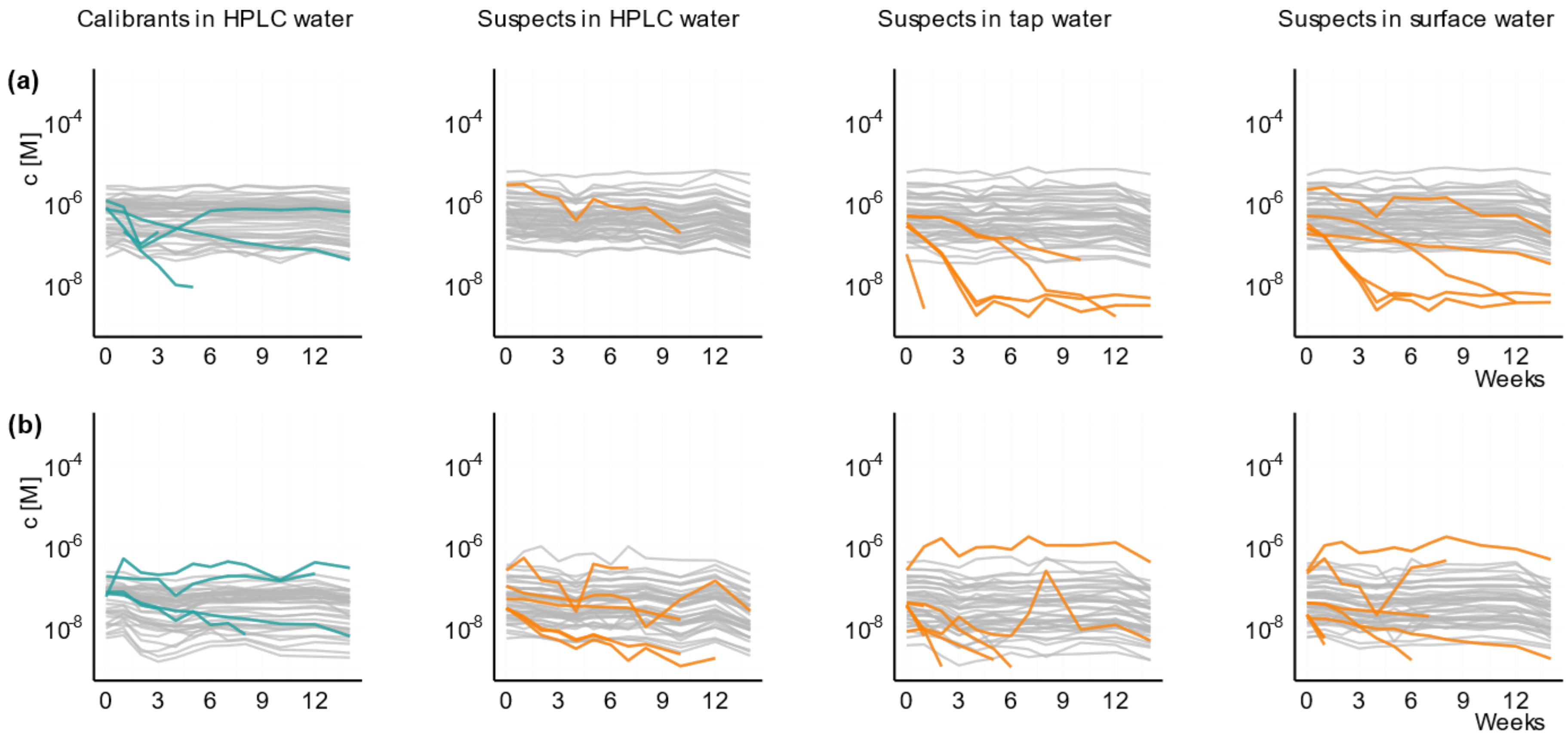


**Fig. S2.** Stability of compounds in freeze-thaw cycles (stored at -20 °C and thawed each week for analysis) in **(a)** high concentrated and **(b)** low concentrated calibration mixes/samples. Degrading calibration compounds (blue lines) are in the high concentrated mix: aspartame, chlormequat, nigericin $H^+$, and trichlorfon, and in the low concentrated mix: aspartame, histamine, L-alanine, and trichlorfon (nigericin $H^+$ n.d.). Suspects showing signs of degradation (orange lines) are, in HPLC water, high concentration: dazomet; in HPLC water, low concentration: chlorpyrifos, dazomet, melamine, simvastatin $H^+$, simvastatin $Na^+$, and simvastatin $NH_4^+$; in tap water, high concentration: ampicillin, chlorpyrifos, omethoate, simvastatin $H^+$, simvastatin $Na^+$, and simvastatin $NH_4^+$ (dazomet n.d.); in tap water, low concentration: atrazine-desethyl-desisopropyl-2-hydroxy, butylamine, chlorpyrifos, omethoate, simvastatin $H^+$, simvastatin $Na^+$, and simvastatin $NH_4^+$ (ampicillin and dazomet n.d.); in surface water, high concentration: ampicillin, dazomet, omethoate, simvastatin $H^+$, simvastatin $Na^+$, and simvastatin $NH_4^+$; and in surface water, low concentration: ampicillin, butylamine, chlorpyrifos, dazomet, omethoate, simvastatin $Na^+$, and simvastatin $NH_4^+$ (simvastatin $H^+$ n.d.).

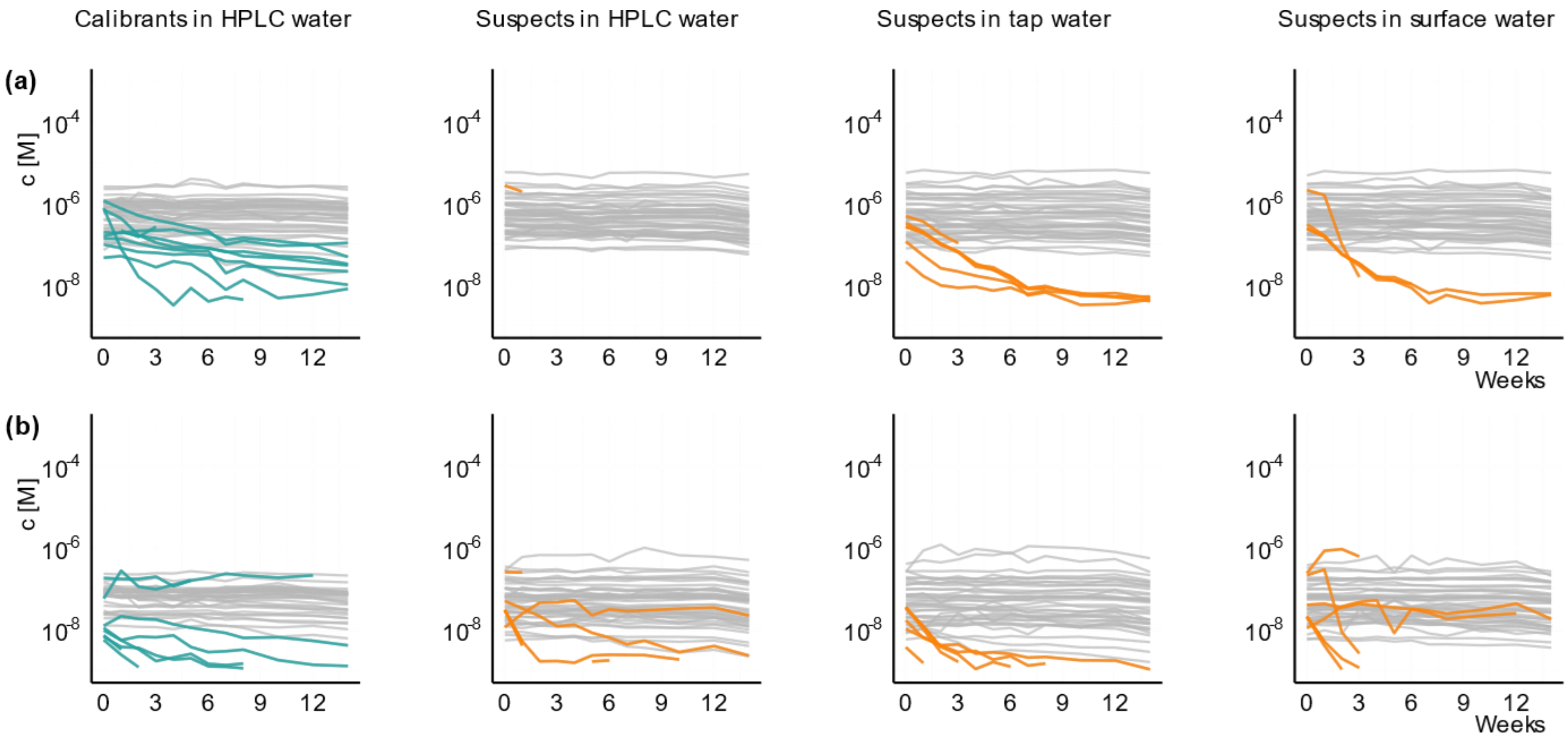


**Fig. S3.** Stability of compounds stored at 4 °C in **(a)** high concentrated and **(b)** low concentrated calibration mixes/samples. Degrading calibration compounds (blue lines) are in the high concentrated mix: aspartame, avermectin B1a $H^+$, avermectin B1a $NH_4^+$, emamectin B1a, ivermectin B1a $NH_4^+$, nigericin $H^+$, octocrylene, rifaximin, and trichlorfon, and in the low concentrated mix: avermectin B1a $NH_4^+$, emamectin B1a, histamine, ivermectin B1a $NH_4^+$, L-alanine, nigericin $NH_4^+$, rifaximin, and spinosad A (avermectin B1a $H^+$, nigericin $H^+$, and octocrylene n.d.). Suspects showing signs of degradation (orange lines) are, in HPLC water, high concentration: dazomet; in HPLC water, low concentration: atrazine-desisopropyl-2-hydroxy, dazomet, reserpine, simvastatin $H^+$, simvastatin $Na^+$, and simvastatin $NH_4^+$; in tap water, high concentration: chlorpyrifos, clotrimazole, simvastatin $H^+$, simvastatin $Na^+$, simvastatin $NH_4^+$, and sudan I (ampicillin and dazomet n.d.); in tap water, low concentration: clotrimazole, ketoconazole, simvastatin $H^+$, simvastatin $Na^+$, simvastatin $NH_4^+$, and sudan I (ampicillin, chlorpyrifos, and dazomet n.d.); in surface water, high concentration: dazomet, simvastatin $H^+$, simvastatin $Na^+$, and simvastatin $NH_4^+$; and in surface water, low concentration: atrazine-desisopropyl-2-hydroxy, butylamine, chlorpyrifos, dazomet, simvastatin $H^+$, simvastatin $Na^+$, and simvastatin $NH_4^+$.

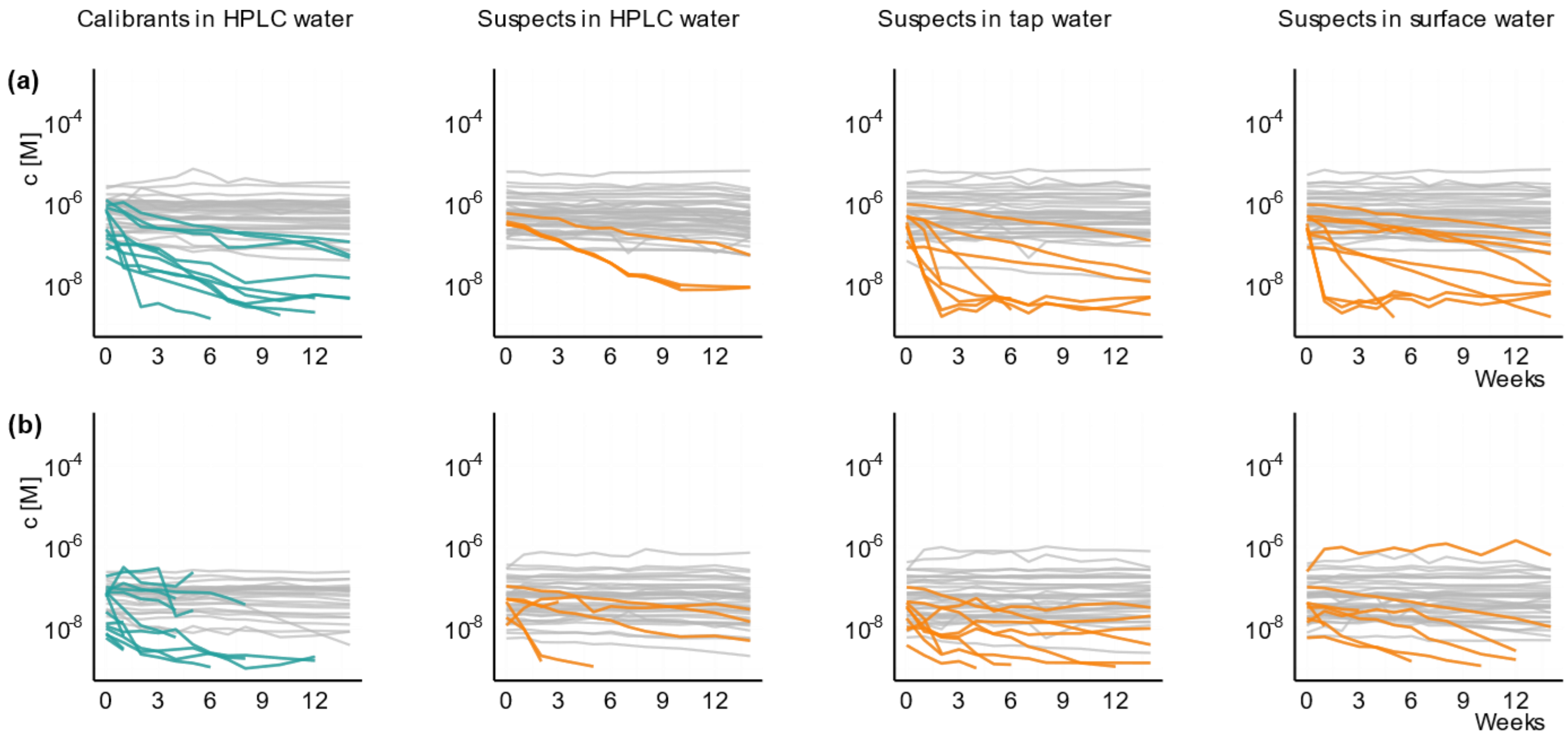


**Fig. S4.** Stability of compounds stored at 20 – 25 °C in **(a)** high concentrated and **(b)** low concentrated calibration mixes/samples. Degrading calibration compounds (blue lines) are in the high concentrated mix: aspartame, avermectin B1a $H^+$, avermectin B1a $NH_4^+$, cefoperazone, dichlorvos, diphenyl phthalate, emamectin B1a, ivermectin B1a $NH_4^+$, nigericin $H^+$, octocrylene, rifaximin, and TCMTB (trichlorfon n.d.), and in the low concentrated mix: aspartame, avermectin $NH_4^+$, cefoperazone, dichlorvos, dimethyl phthalate, diphenyl phthalate, emamectin B1a, histamine, ivermectin B1a $NH_4^+$, L-alanine, nigericin $NH_4^+$, rifaximin, spinosad A, and TCMTB (avermectin B1a $H^+$, nigericin $H^+$, octocrylene, and trichlorfon n.d.). Suspects showing signs of degradation (orange lines) are, in HPLC water, high concentration: chlorpyrifos, simvastatin $H^+$, simvastatin $Na^+$, and simvastatin $NH_4^+$ (dazomet n.d.); in HPLC water, low concentration: ampicillin, atrazine-desisopropyl-2-hydroxy, chlorpyrifos, methidathion, omethoate, reserpine (dazomet, simvastatin $H^+$, simvastatin $Na^+$, and simvastatin $NH_4^+$ n.d.) ; in tap water, high concentration: acephate, chlorpyrifos, methidathion, omethoate, reserpine, simvastatin $H^+$, simvastatin $Na^+$, simvastatin $NH_4^+$, and sudan I (ampicillin and dazomet n.d.); in tap water, low concentration: 5-chlorobenzotriazole, 5-methyl-1H-benzotriazole, atrazine-desisopropyl-2-hydroxy, clotrimazole, ketoconazole, methidathion, omethoate, reserpine, and sudan I (ampicillin, chlorpyrifos, dazomet, simvastatin $H^+$, simvastatin $Na^+$, and simvastatin $NH_4^+$ n.d.); in surface water, high concentration: acephate, adenosine, ampicillin, chlorpyrifos, methidathion, omethoate, reserpine, simvastatin $H^+$, simvastatin $Na^+$, and simvastatin $NH_4^+$ (dazomet n.d.); and in surface water, low concentration: acephate, adenosine, ampicillin, butylamine, chlorpyrifos, methidathion, omethoate, and reserpine (dazomet, simvastatin $H^+$, simvastatin $Na^+$, and simvastatin $NH_4^+$ n.d.).

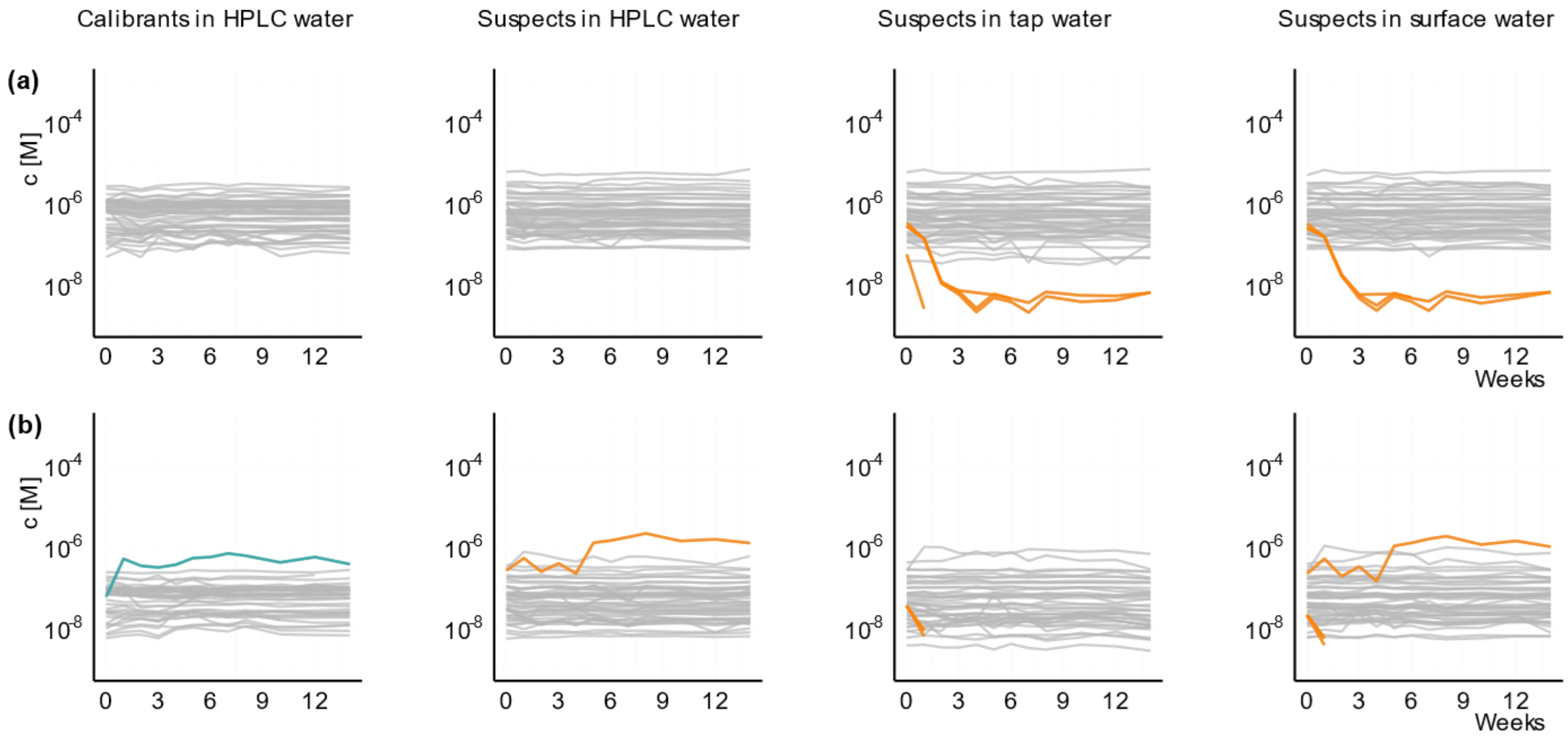


**Fig. S5.** Stability of compounds stored at -20 °C in **(a)** high concentrated and **(b)** low concentrated calibration mixes/samples. Degrading calibration compounds (blue lines) are in the low concentrated mix: histamine. Suspects showing signs of degradation (orange lines) are, in HPLC water, low concentration: dazomet; in tap water, high concentration: ampicillin, simvastatin $H^+$, simvastatin $Na^+$, and simvastatin $NH_4^+$ (dazomet n.d.); in tap water, low concentration: simvastatin $H^+$, simvastatin $Na^+$, and simvastatin $NH_4^+$ (ampicillin and dazomet n.d.); in surface water, high concentration: simvastatin $H^+$, simvastatin $Na^+$, and simvastatin $NH_4^+$; and in surface water, low concentration: dazomet, simvastatin $H^+$, simvastatin $Na^+$, and simvastatin $NH_4^+$.

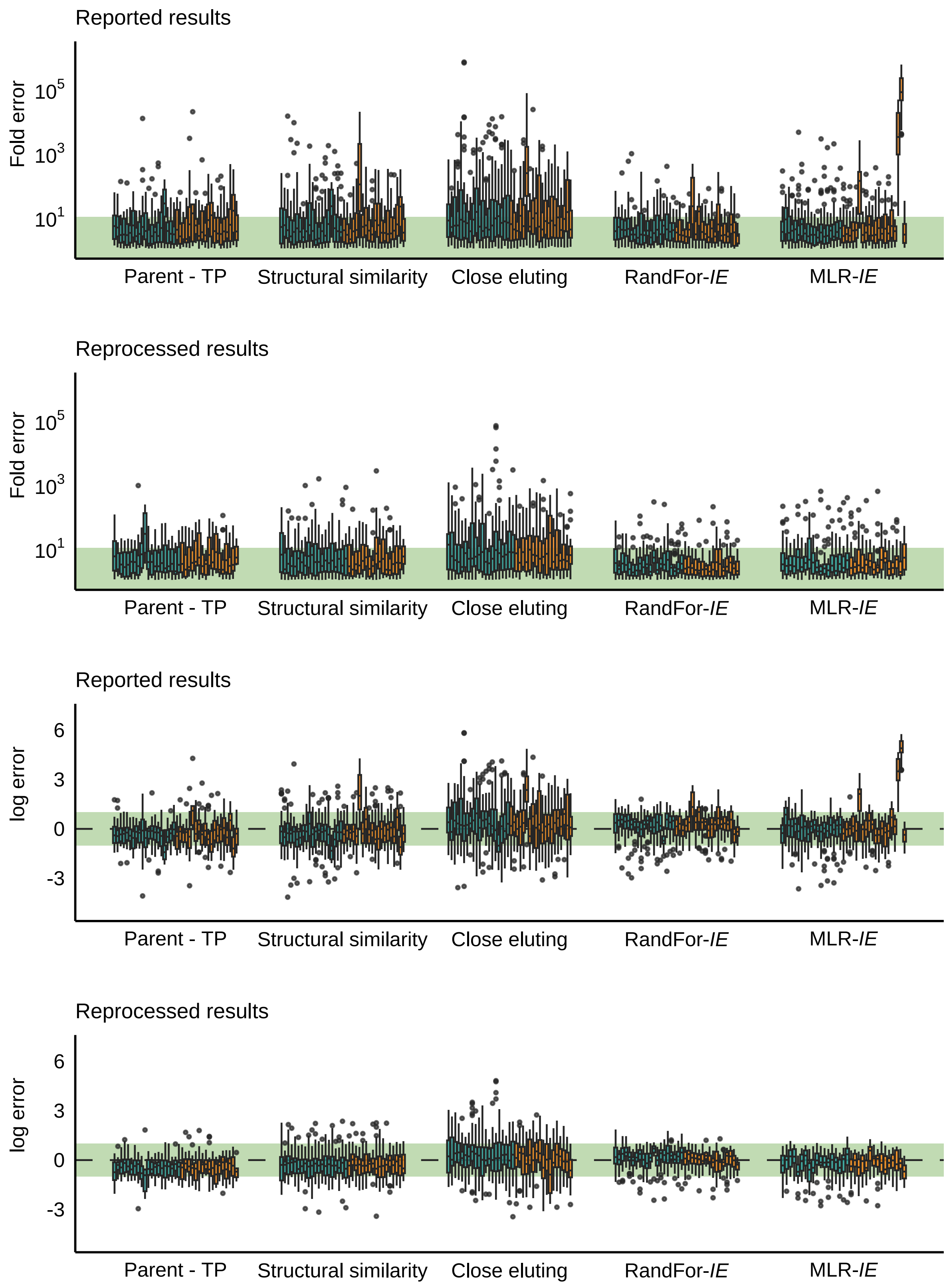


**Fig. S6.** The prediction errors of each quantification approach across the datasets for sample s1b (low concentrated spike in HPLC water). The green area shows the 10× error and the equivalent log error.

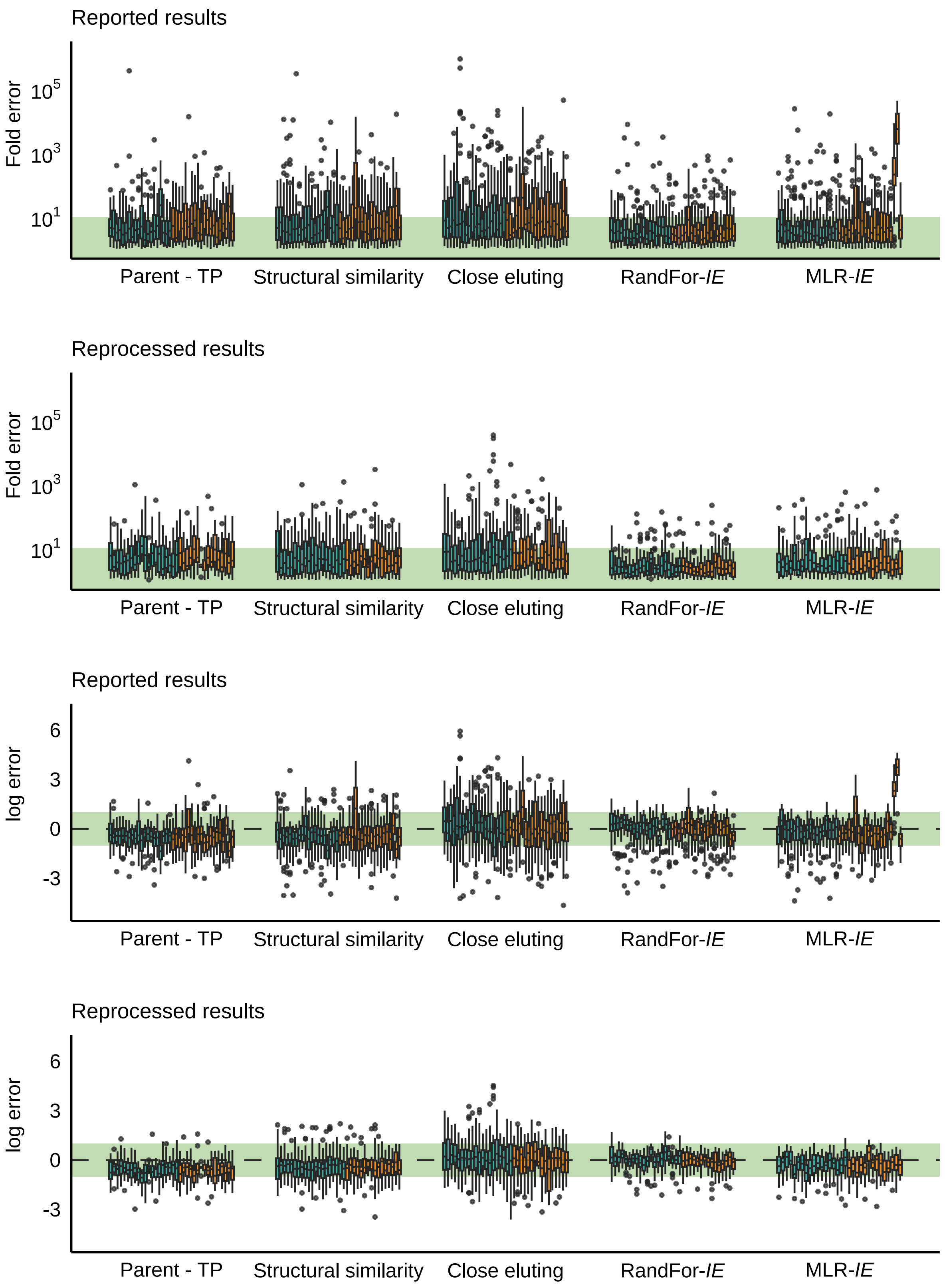


**Fig. S7.** The prediction errors of each quantification approach across the datasets for sample s2a (high concentrated spike in tap water). The green area shows the 10× error and the equivalent log error.

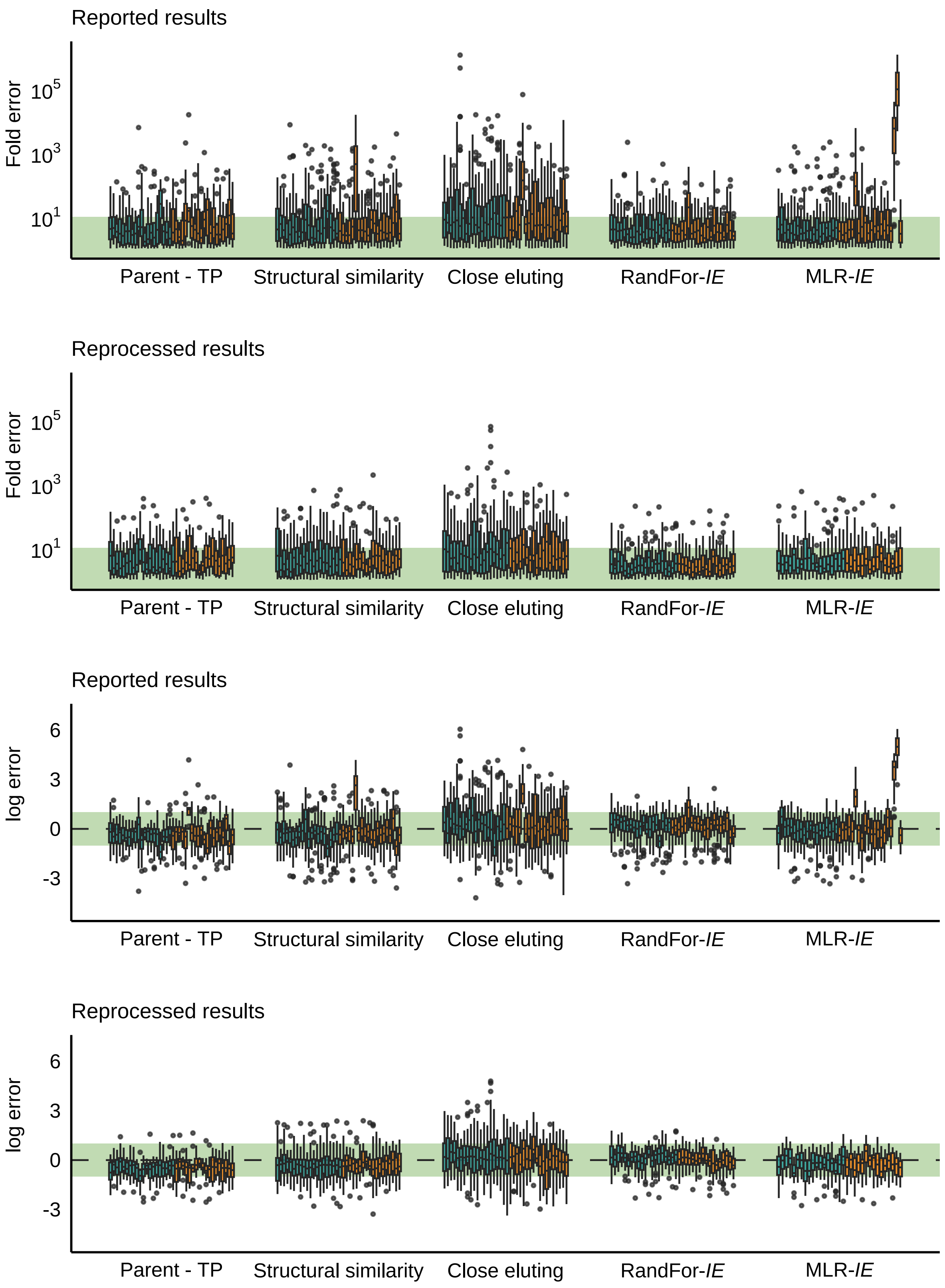


**Fig. S8.** The prediction errors of each quantification approach across the datasets for sample s2b (low concentrated spike in tap water). The green area shows the 10× error and the equivalent log error.

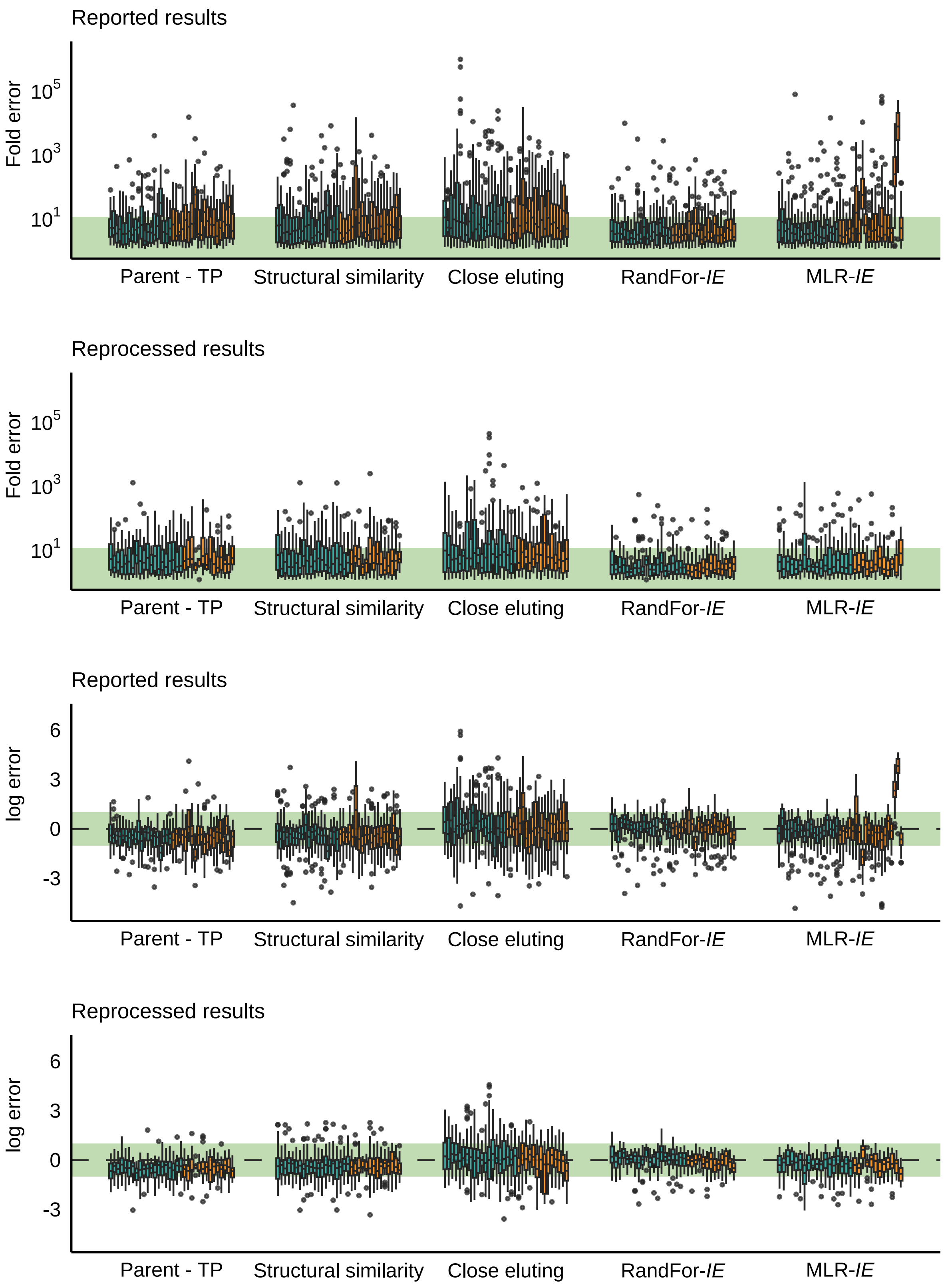


**Fig. S9.** The prediction errors of each quantification approach across the datasets for sample s3a (high concentrated spike in surface water). The green area shows the 10× error and the equivalent log error.

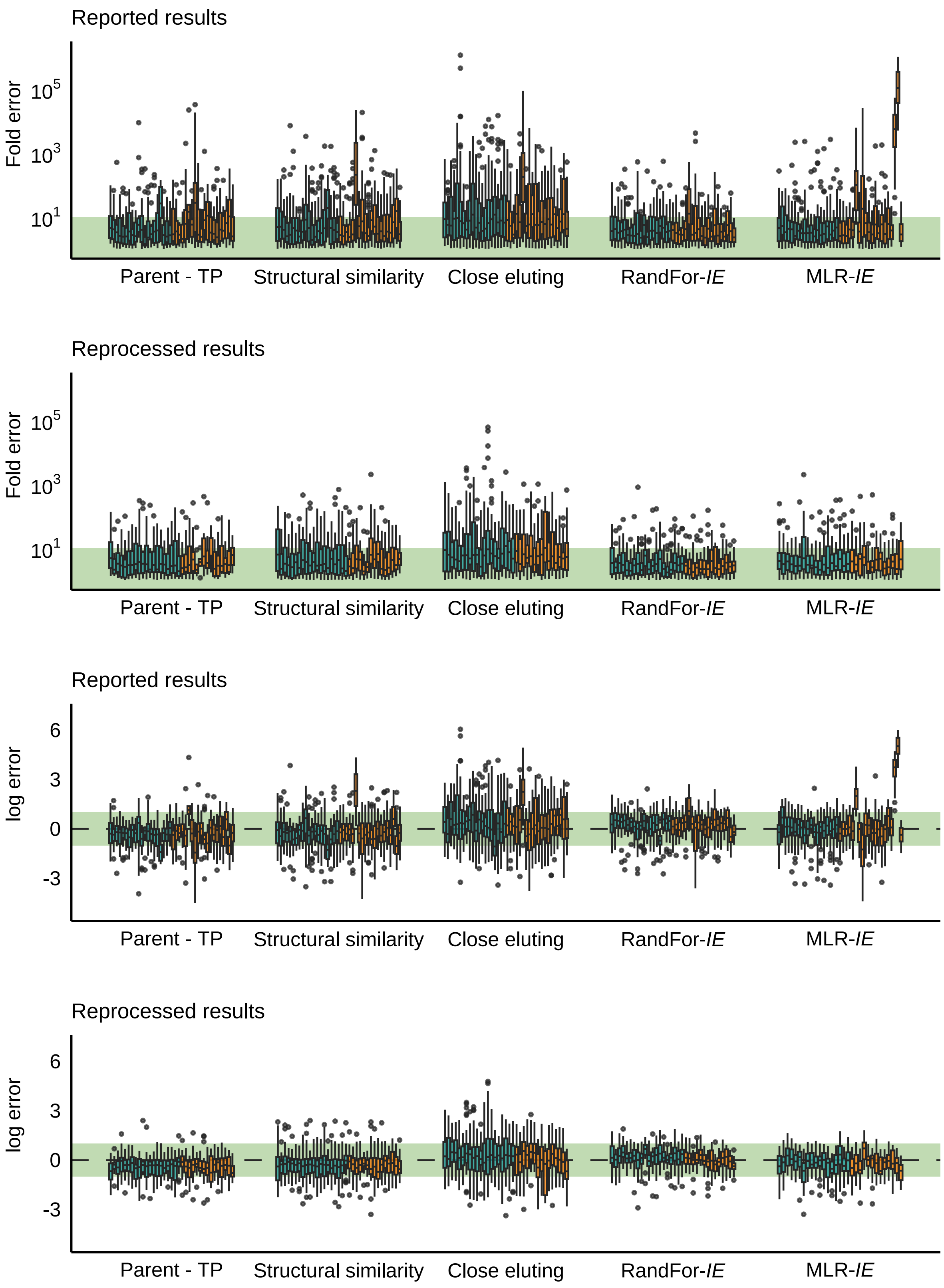


**Fig. S10.** The prediction errors of each quantification approach across the datasets for sample s3b (low concentrated spike in surface water). The green area shows the 10× error and the equivalent log error.

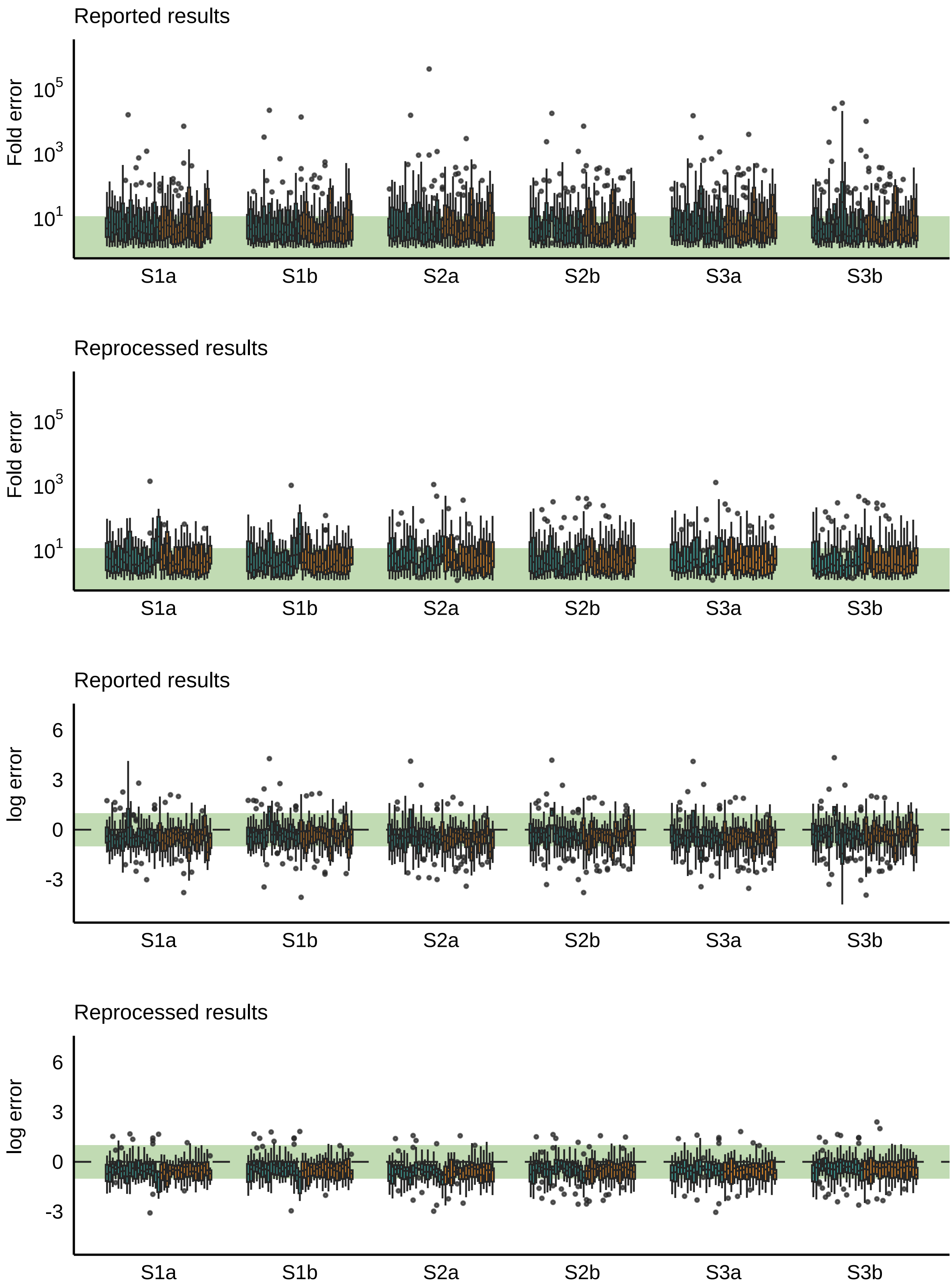


**Fig. S11.** The prediction errors across all samples for the parent-TP approach. The green area shows the 10× error and the equivalent log error.

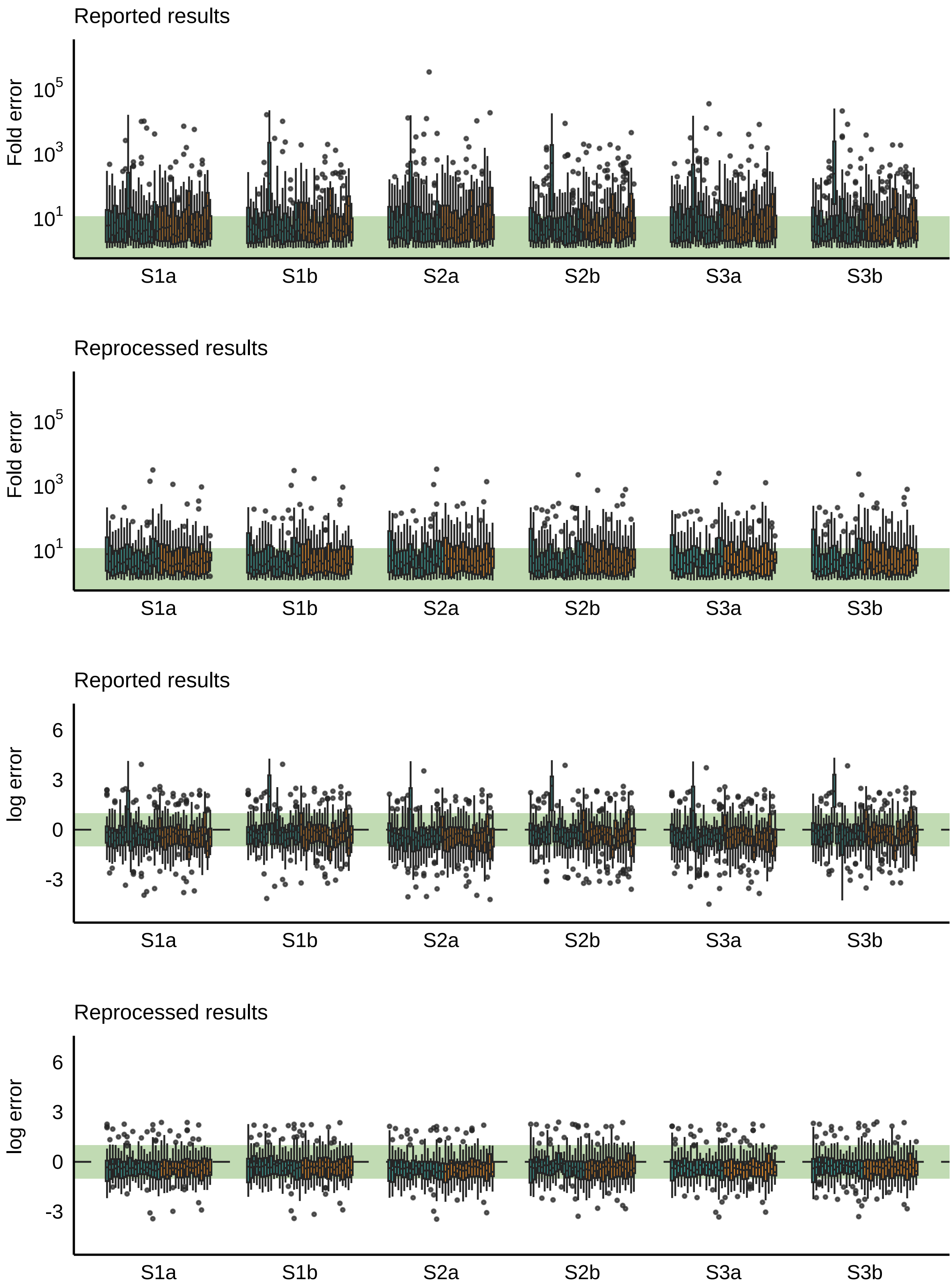


**Fig. S12.** The prediction errors across all samples for the structural similarity approach. The green area shows the 10× error and the equivalent log error.

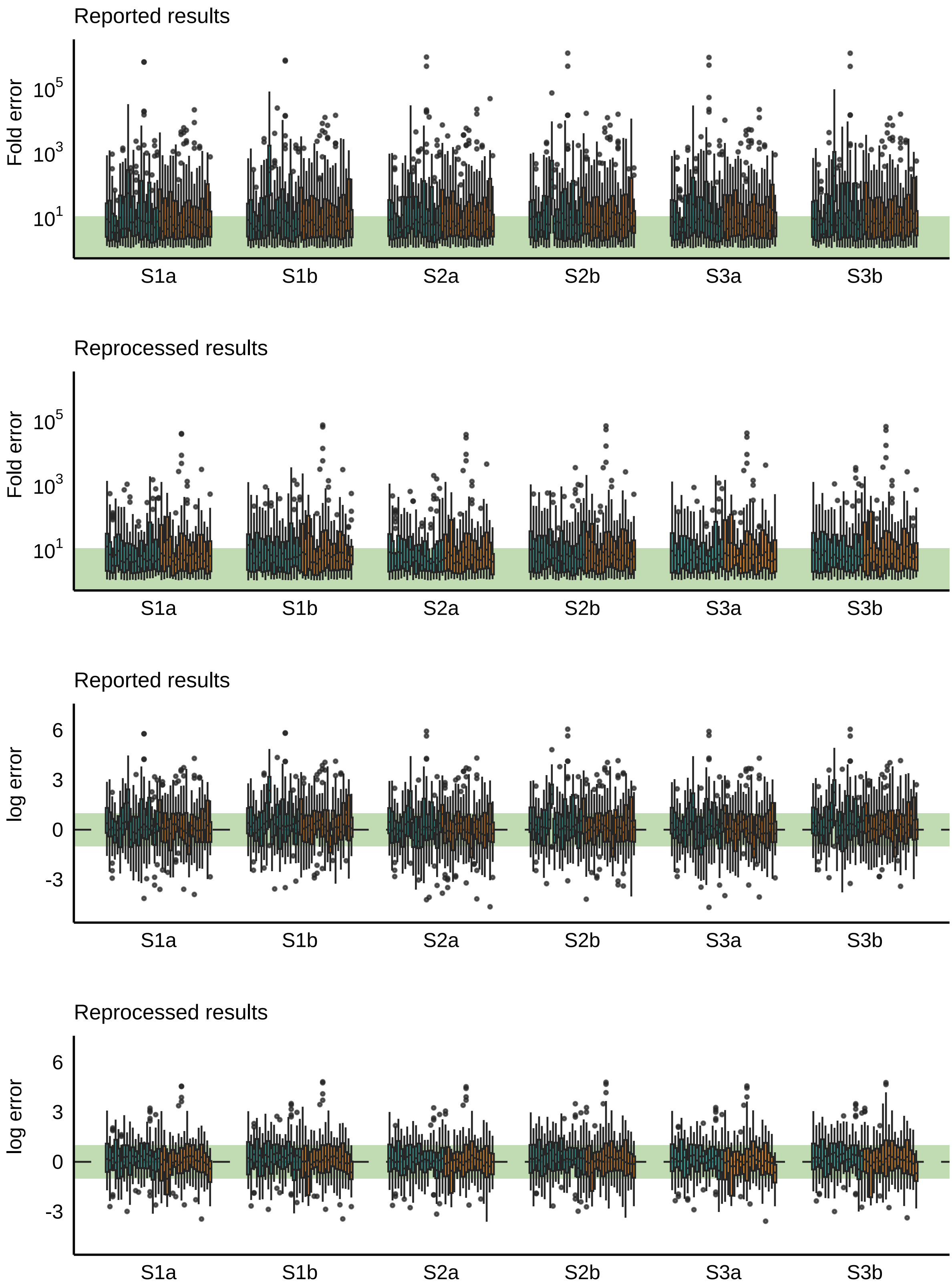


**Fig. S13.** The prediction errors across all samples for the close eluting approach. The green area shows the 10× error and the equivalent log error.

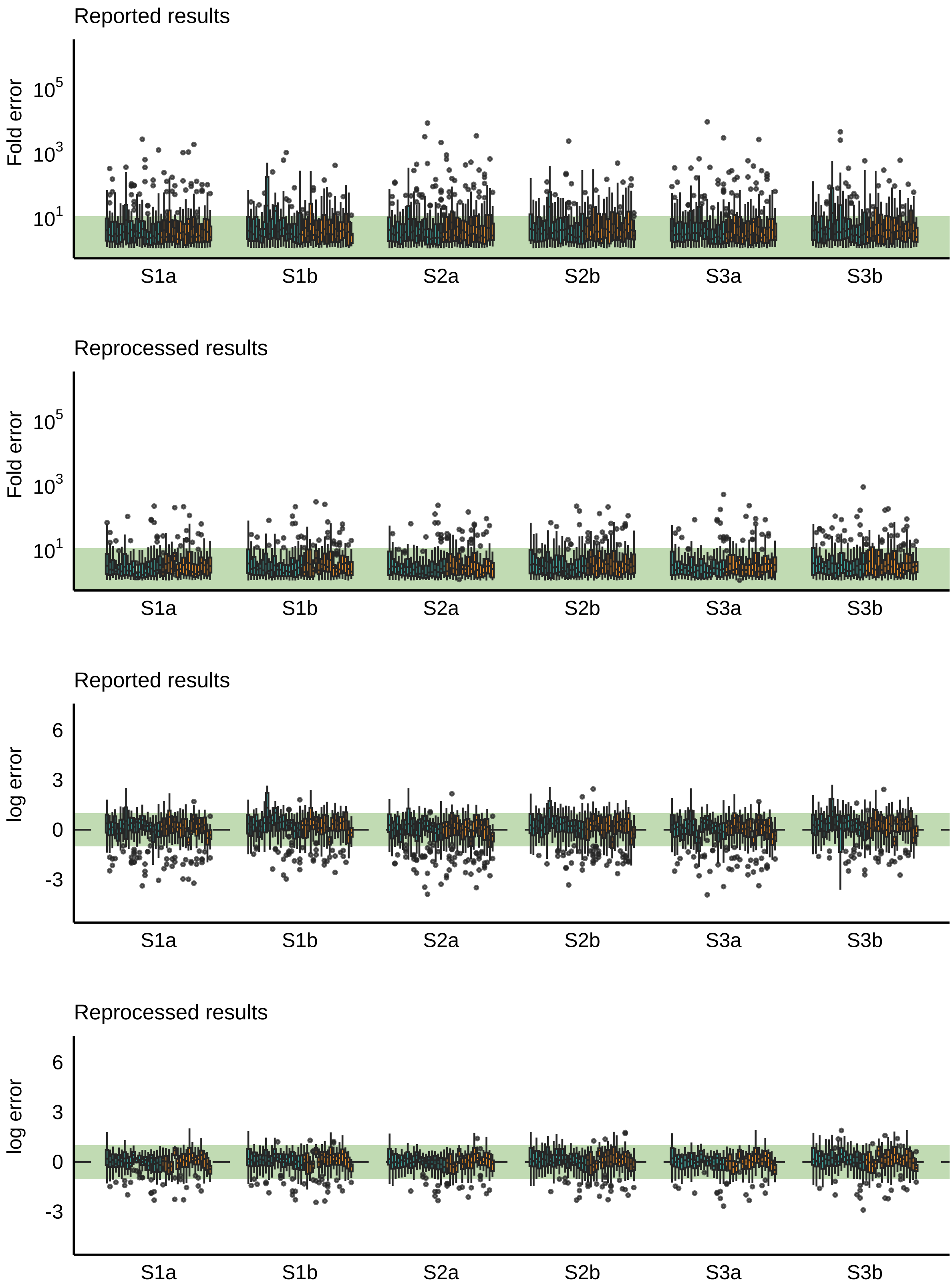


**Fig. S14.** The prediction errors across all samples for the RandFor-*IE* approach. The green area shows the 10× error and the equivalent log error.

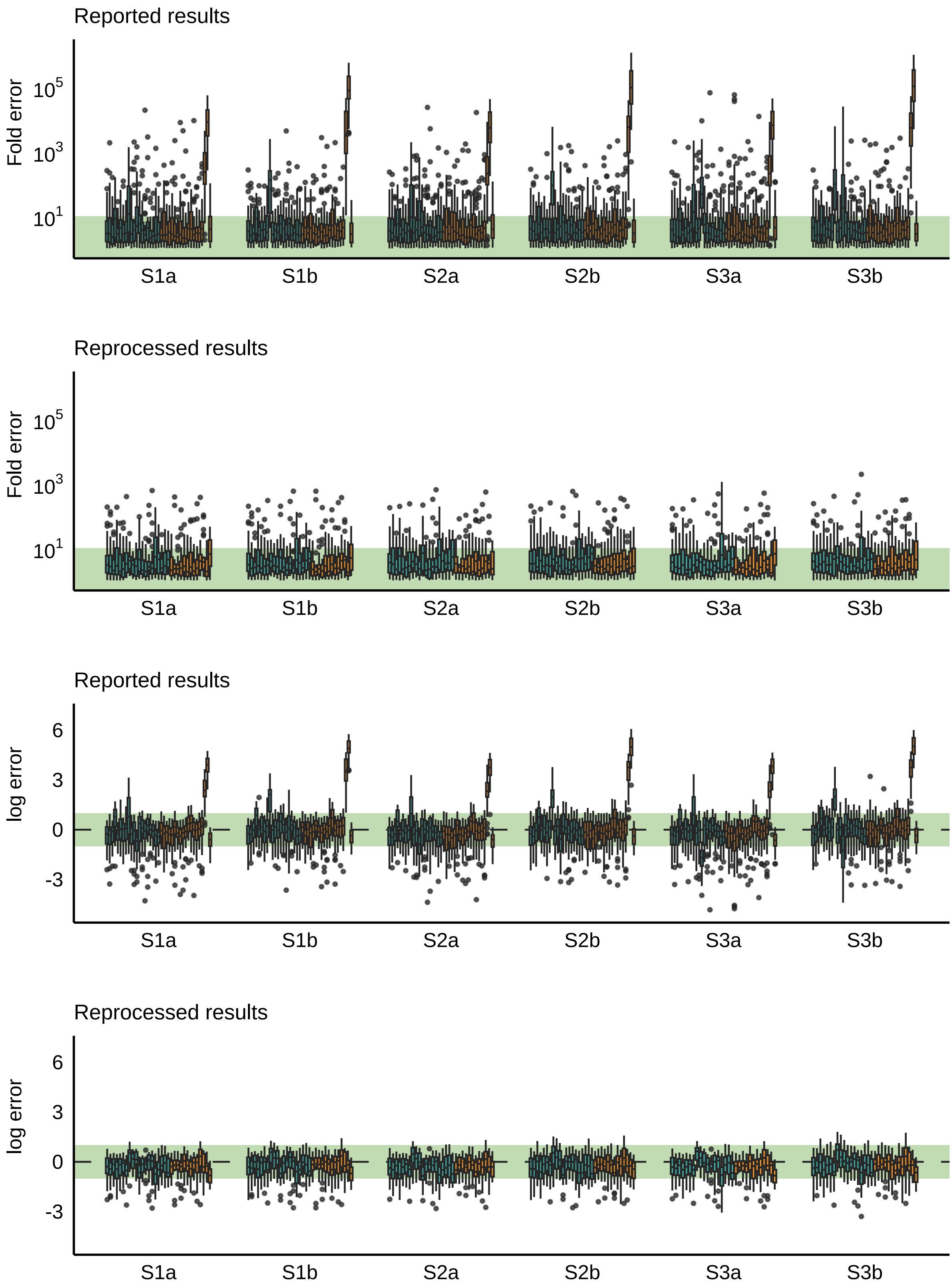


**Fig. S15.** The prediction errors across all samples for the MLR-*IE* approach. The green area shows the 10× error and the equivalent log error.

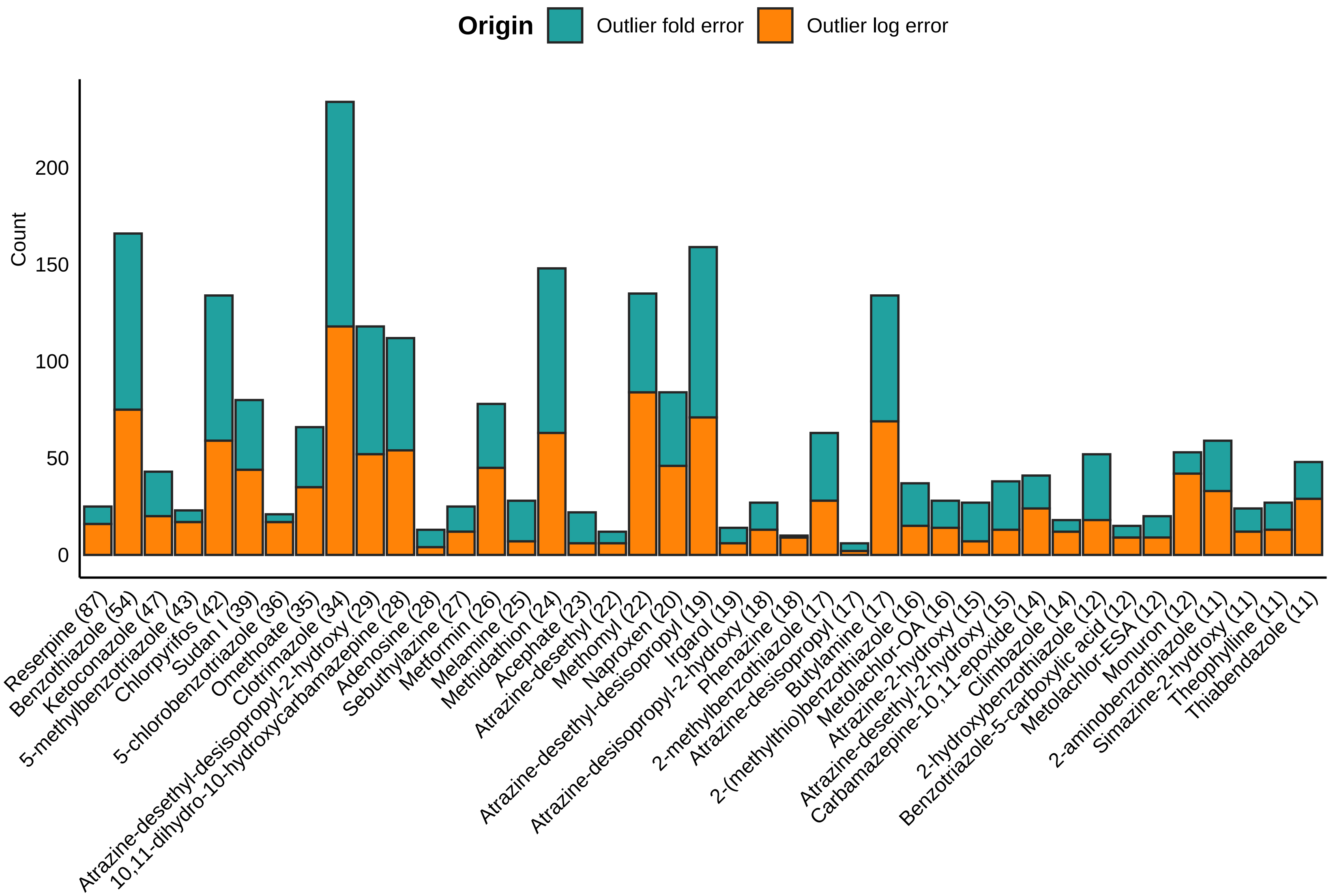


**Fig. S16**. Bar plot with the outliers' occurrences for all compounds, sorted based on their count as an out-of-range peak area ratio (from highest to lowest count, shown in the parenthesis).

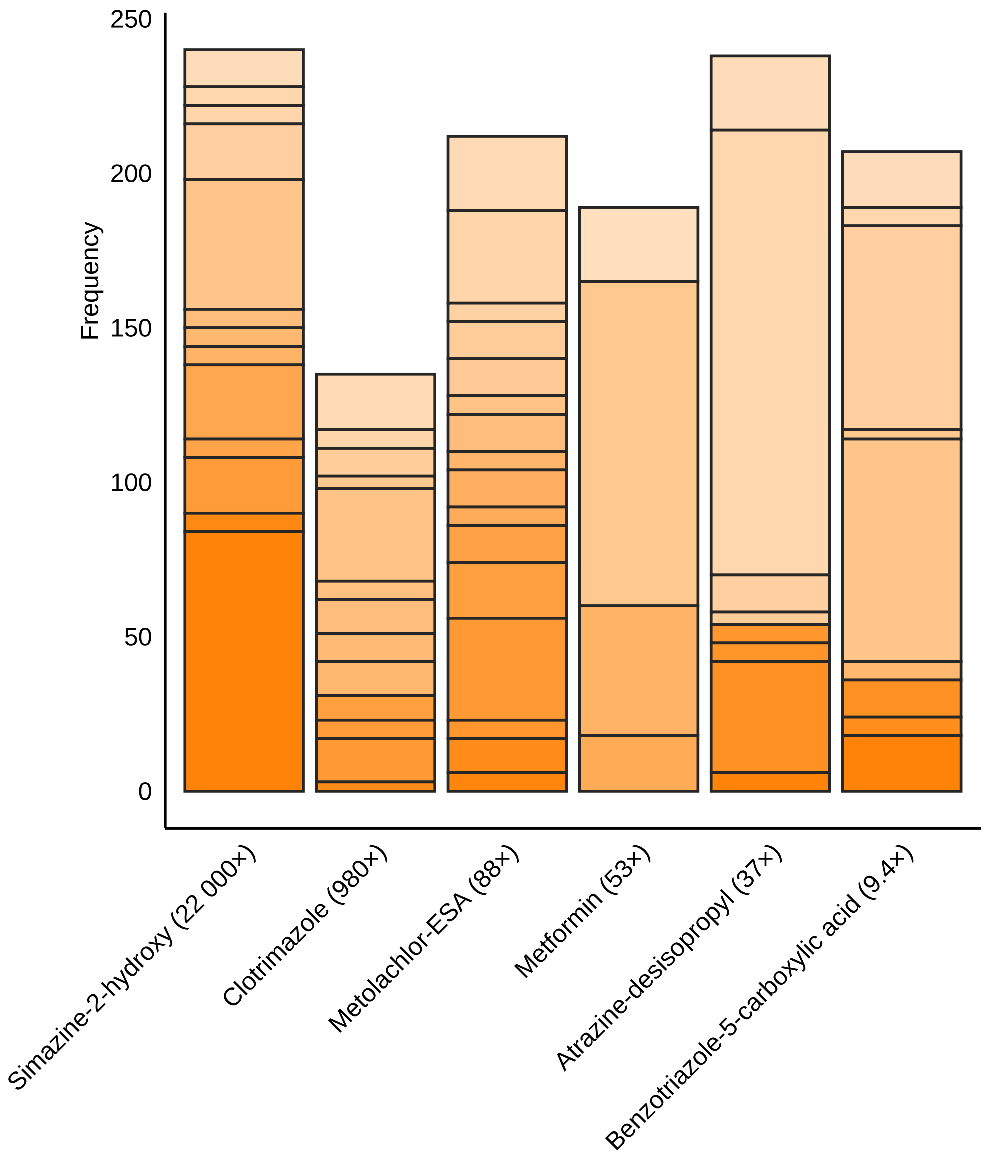


**Fig. S17.** The proportion of closest eluting calibrant assignments across the datasets for the suspect compounds with highest and lowest number of assignments (metolachlor-ESA and metformin), highest and lowest maximum proportion of same close eluting assignment (atrazine-desisopropyl and clotrimazole), and highest and lowest average fold error across the datasets and samples (simazine-2-hydroxy and benzotriazole-5-carboxylic acid). The suspect compounds are sorted from highest to lowest average fold error (shown in parenthesis).

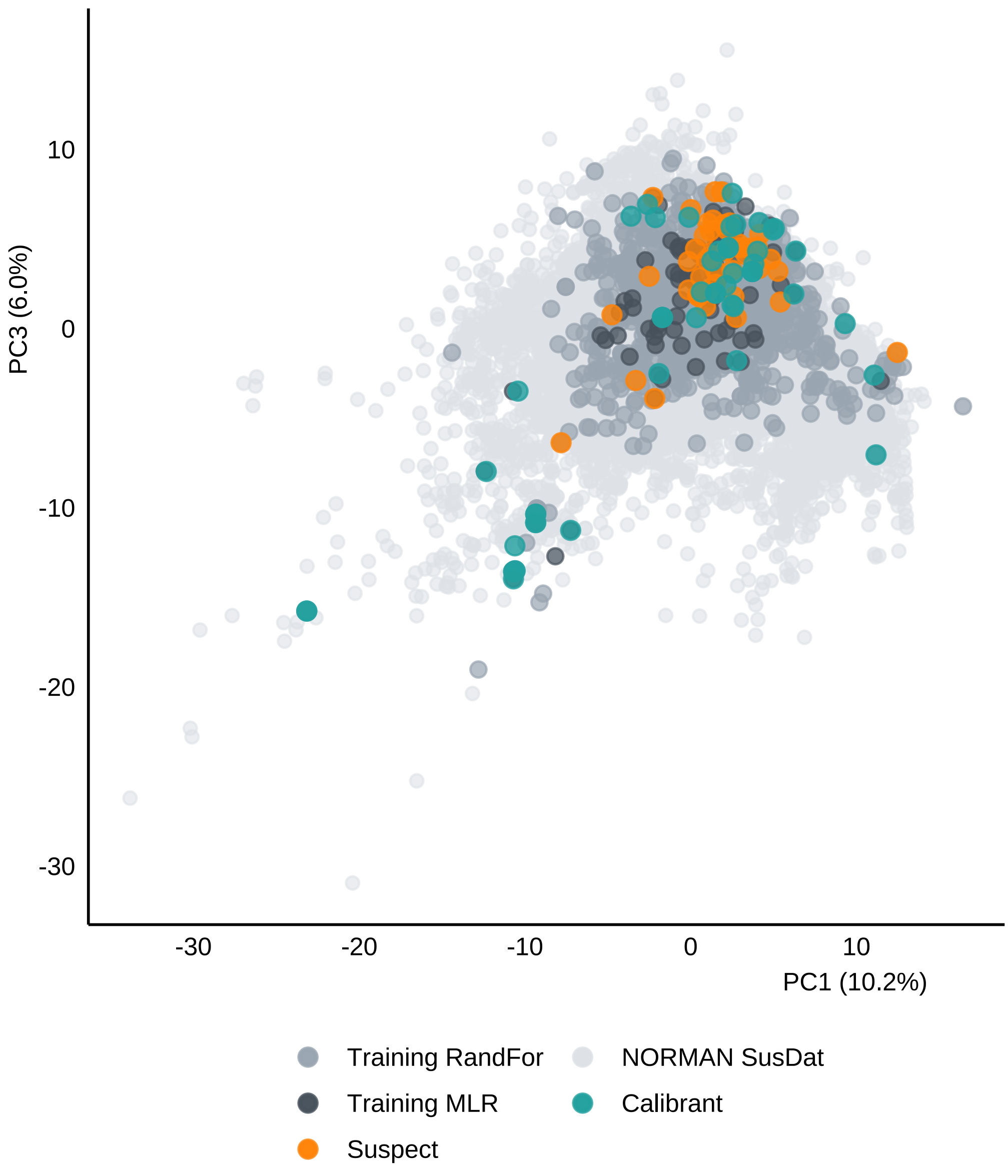


**Fig. S18.** Plot of PC1 vs PC3 for the suspects and calibrants together with the training compounds for the *IE*-based approaches and the LC/ESI(+) amenable compounds from NORMAN SusDat (Prob. RPLC ≥ 0.5, Prob- +ESI ≥ 0.5). PCA based on Mordred descriptors.

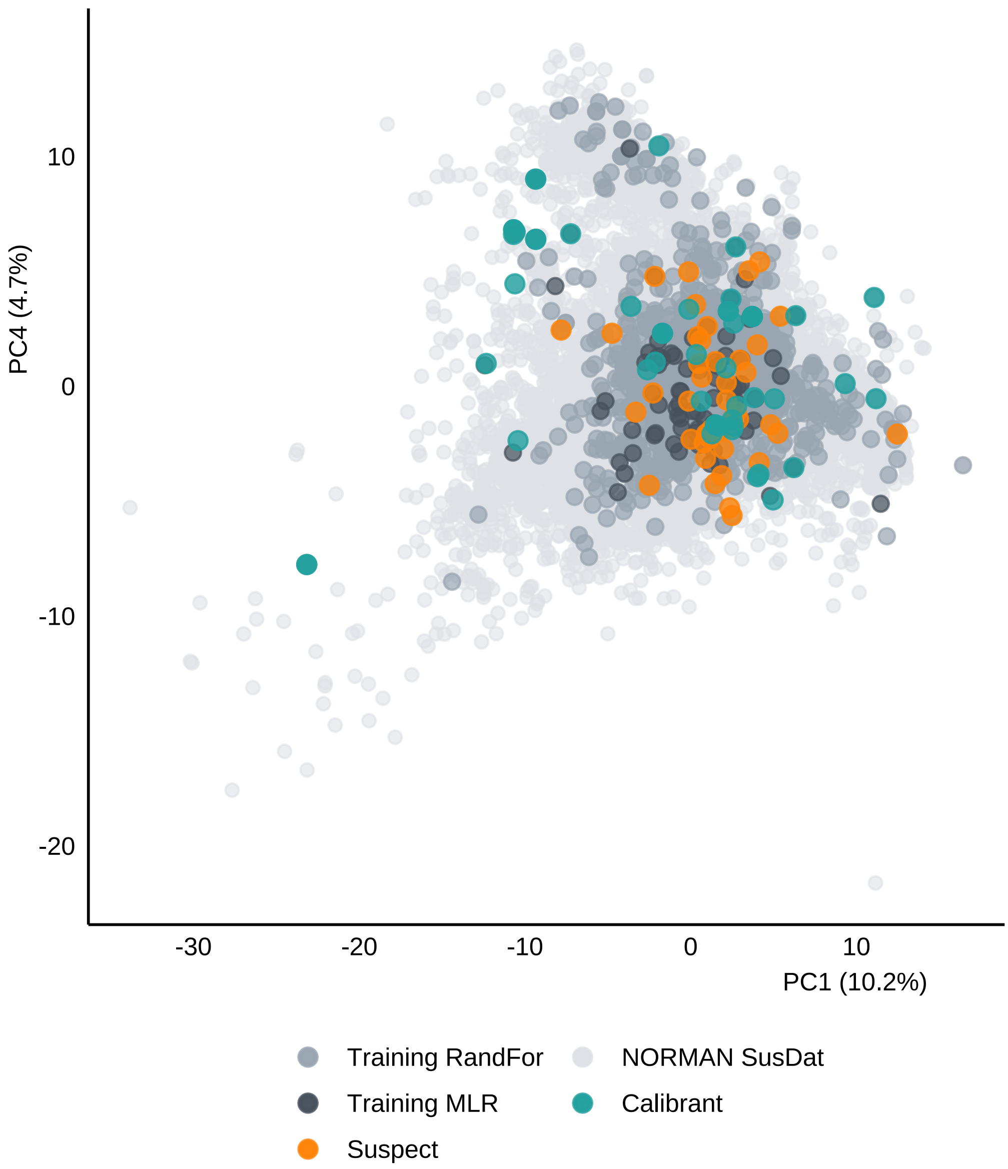


**Fig. S19.** Plot of PC1 vs PC4 for the suspects and calibrants together with the training compounds for the *IE*-based approaches and the LC/ESI(+) amenable compounds from NORMAN SusDat (Prob. RPLC ≥ 0.5, Prob- +ESI ≥ 0.5). PCA based on Mordred descriptors.

## References


(1) Senta, I.; Kostanjevecki, P.; Krizman-Matasic, I.; Terzic, S.; Ahel, M. Occurrence and Behavior of Macrolide Antibiotics in Municipal Wastewater Treatment: Possible Importance of Metabolites, Synthesis Byproducts, and Transformation Products. *Environ. Sci. Technol.* **2019**, *53* (13), 7463–7472. https://doi.org/10.1021/acs.est.9b01420.

(2) Kruve, A.; Kiefer, K.; Hollender, J. Benchmarking of the Quantification Approaches for the Non-Targeted Screening of Micropollutants and Their Transformation Products in Groundwater. *Anal Bioanal Chem* **2021**, *413* (6), 1549–1559. https://doi.org/10.1007/s00216-020-03109-2.

(3) Dahal, U. P.; Jones, J. P.; Davis, J. A.; Rock, D. A. Small Molecule Quantification by Liquid Chromatography-Mass Spectrometry for Metabolites of Drugs and Drug Candidates. *Drug Metab. Dispos.* **2011**, *39* (12), 2355–2360. https://doi.org/10.1124/dmd.111.040865.

(4) Solliec, M.; Roy-Lachapelle, A.; Storck, V.; Callender, K.; Greer, C. W.; Barbeau, B. A Data-Independent Acquisition Approach Based on HRMS to Explore the Biodegradation Process of Organic Micropollutants Involved in a Biological Ion-Exchange Drinking Water Filter. *Chemosphere* **2021**, *277*, 130216. https://doi.org/10.1016/j.chemosphere.2021.130216.

(5) Kalogiouri, N. P.; Aalizadeh, R.; Thomaidis, N. S. Investigating the Organic and Conventional Production Type of Olive Oil with Target and Suspect Screening by LC-QTOF-MS, a Novel Semi-Quantification Method Using Chemical Similarity and Advanced Chemometrics. *Anal Bioanal Chem* **2017**, *409* (23), 5413–5426. https://doi.org/10.1007/s00216-017-0395-6.

(6) Chibwe, L.; Parrott, J. L.; Shires, K.; Khan, H.; Clarence, S.; Lavalle, C.; Sullivan, C.; O'Brien, A. M.; De Silva, A. O.; Muir, D. C. G.; Rochman, C. M. A Deep Dive into the Complex Chemical Mixture and Toxicity of Tire Wear Particle Leachate in Fathead Minnow. *Enviro Toxic and Chemistry* **2022**, *41* (5), 1144–1153. https://doi.org/10.1002/etc.5140.

(7) *Similar compound finder*. http://dsfp.chem.uoa.gr/semiquantification/.

(8) Alygizakis, N.; Galani, A.; Rousis, N. I.; Aalizadeh, R.; Dimopoulos, M.-A.; Thomaidis, N. S. Change in the Chemical Content of Untreated Wastewater of Athens, Greece under COVID-19 Pandemic. *Science of The Total Environment* **2021**, *799*, 149230. https://doi.org/10.1016/j.scitotenv.2021.149230.

(9) NORMAN Network; Aalizadeh, Reza; Alygizakis, Nikiforos; Schymanski, Emma; Slobodnik, Jaroslav; Fischer, Stellan; Cirka, Lubos. S0 | SUSDAT | Merged NORMAN Suspect List: SusDat, 2022. https://doi.org/10.5281/ZENODO.2664077.

(10) Chen, X.; Reynolds, C. H. Performance of Similarity Measures in 2D Fragment-Based Similarity Searching: Comparison of Structural Descriptors and Similarity Coefficients. *J. Chem. Inf. Comput. Sci.* **2002**, *42* (6), 1407–1414. https://doi.org/10.1021/ci025531g.

(11) Kim, Y.; Pike, K. A.; Gray, R.; Sprankle, J. W.; Faust, J. A.; Edmiston, P. L. Non-Targeted Identification and Semi-Quantitation of Emerging per- and Polyfluoroalkyl Substances (PFAS) in US Rainwater. *Environ. Sci.: Processes Impacts* **2023**, 10.1039.D2EM00349J. https://doi.org/10.1039/D2EM00349J.

(12) Zang, L.; Wan, Y.; Zhang, H.; Zhang, Y.; Gao, Y.; He, Y.; Hu, J.; Kang, Y.; Cao, D.; Yang, M. Characterization of Non-Volatile Organic Contaminants in Coking Wastewater Using Non-Target Screening: Dominance of Nitrogen, Sulfur, and Oxygen-Containing Compounds in Biological Effluents. *Sci. Total Environ.* **2022**, *837*, 155768. https://doi.org/10.1016/j.scitotenv.2022.155768.

(13) Pieke, E. N.; Granby, K.; Trier, X.; Smedsgaard, J. A Framework to Estimate Concentrations of Potentially Unknown Substances by Semi-Quantification in Liquid Chromatography Electrospray Ionization Mass Spectrometry. *Analytica Chimica Acta* **2017**, *975*, 30–41. https://doi.org/10.1016/j.aca.2017.03.054.

(14) Liigand, J.; Wang, T.; Kellogg, J. J.; Smedsgaard, J.; Cech, N. B. Quantification for Non-Targeted LC/MS Screening without Standard Substances: SI. **2020**, 53.

(15) Wang, T.; Liigand, J.; Frandsen, H. L.; Smedsgaard, J.; Kruve, A. Standard Substances Free Quantification Makes LC/ESI/MS Non-Targeted Screening of Pesticides in Cereals Comparable between Labs. *Food Chem.* **2020**, *318*, 126460. https://doi.org/10.1016/j.foodchem.2020.126460.

(16) Been, F.; Kruve, A.; Vughs, D.; Meekel, N.; Reus, A.; Zwartsen, A.; Wessel, A.; Fischer, A.; ter Laak, T.; Brunner, A. M. Risk-Based Prioritization of Suspects Detected in Riverine Water Using Complementary Chromatographic Techniques. *Water Res.* **2021**, *204*, 117612. https://doi.org/10.1016/j.watres.2021.117612.

(17) Nanusha, M. Y.; Frøkjær, E. E.; Liigand, J.; Christensen, M. R.; Hansen, H. R.; Hansen, M. Unravelling the Occurrence of Trace Contaminants in Surface Waters Using Semi-Quantitative Suspected Non-Target Screening Analyses. *Environ. Pollut.* **2022**, *315*, 120346. https://doi.org/10.1016/j.envpol.2022.120346.

(18) Tadić, Đ.; Manasfi, R.; Bertrand, M.; Sauvêtre, A.; Chiron, S. Use of Passive and Grab Sampling and High-Resolution Mass Spectrometry for Non-Targeted Analysis of Emerging Contaminants and Their Semi-Quantification in Water. *Molecules* **2022**, *27* (10), 3167. https://doi.org/10.3390/molecules27103167.

(19) Lauria, M. Z.; Sepman, H.; Ledbetter, T.; Plassmann, M.; Roos, A. M.; Simon, M.; Benskin, J. P.; Kruve, A. Closing the Organofluorine Mass Balance in Marine Mammals Using Suspect Screening and Machine Learning-Based Quantification. *Environ. Sci. Technol.* **2024**, *58* (5), 2458–2467. https://doi.org/10.1021/acs.est.3c07220.
(20) Aalizadeh, R.; Nikolopoulou, V.; Alygizakis, N.; Slobodnik, J.; Thomaidis, N. S. A Novel Workflow for Semi-Quantification of Emerging Contaminants in Environmental Samples Analyzed by LC-HRMS. *Anal Bioanal Chem* **2022**, *414* (25), 7435–7450. https://doi.org/10.1007/s00216-022-04084-6.
(21) Chambers, M. C.; Maclean, B.; Burke, R.; Amodei, D.; Ruderman, D. L.; Neumann, S.; Gatto, L.; Fischer, B.; Pratt, B.; Egertson, J.; Hoff, K.; Kessner, D.; Tasman, N.; Shulman, N.; Frewen, B.; Baker, T. A.; Brusniak, M.-Y.; Paulse, C.; Creasy, D.; Flashner, L.; Kani, K.; Moulding, C.; Seymour, S. L.; Nuwaysir, L. M.; Lefebvre, B.; Kuhlmann, F.; Roark, J.; Rainer, P.; Detlev, S.; Hemenway, T.; Huhmer, A.; Langridge, J.; Connolly, B.; Chadick, T.; Holly, K.; Eckels, J.; Deutsch, E. W.; Moritz, R. L.; Katz, J. E.; Agus, D. B.; MacCoss, M.; Tabb, D. L.; Mallick, P. A Cross-Platform Toolkit for Mass Spectrometry and Proteomics. *Nat. Biotechnol.* **2012**, *30* (10), 918–920. https://doi.org/10.1038/nbt.2377.
(22) Helmus, R.; Van De Velde, B.; Brunner, A. M.; Ter Laak, T. L.; Van Wezel, A. P.; Schymanski, E. L. patRoon 2.0: Improved Non-Target Analysis Workflowsincluding Automated Transformation Product Screening. *JOSS* **2022**, 7 (71), 4029. https://doi.org/10.21105/joss.04029.
(23) R Core Team. R: A Language and Environment for Statistical Computing, 2021. https://www.R-project.org/ (accessed 2022-03-10).
